# Dactylfungins and Tetralones: Bioactive Metabolites from a Nematode-Associated *Laburnicola nematophila*

**DOI:** 10.1021/acs.jnatprod.4c00623

**Published:** 2024-07-16

**Authors:** Jan-Peer Wennrich, Caren Holzenkamp, Miroslav Kolařík, Wolfgang Maier, Attila Mándi, Tibor Kurtán, Samad Ashrafi, Sherif S. Ebada, Marc Stadler

**Affiliations:** † Department of Microbial Drugs, Helmholtz Centre for Infection Research (HZI) and German Centre for Infection Research, Inhoffenstrasse 7, 38124 Braunschweig, Germany; ‡ Institute of Microbiology, Technische Universität Braunschweig, Spielmannstraße 7, 38106 Braunschweig, Germany; § Institute of Microbiology, Czech Academy of Science, Vídeňská 1083, 14220 Prague, Czech Republic; ⊥ Institute for Epidemiology and Pathogen Diagonstics, Julius Kühn Institut (JKI) - Federal Research Centre for Cultivated Plants, Messeweg 11-12, 38104 Braunschweig, Germany; ¶ Department of Organic Chemistry, University of Debrecen, P.O. Box 400, 4002 Debrecen, Hungary; ∇ Institute for Crop and Soil Science, Julius Kühn Institute (JKI) − Federal Research Centre for Cultivated Plants, Bundesallee 58, 38116 Braunschweig, Germany; Δ Department of Pharmacognosy, Faculty of Pharmacy, Ain Shams University, Cairo 11566, Egypt

## Abstract

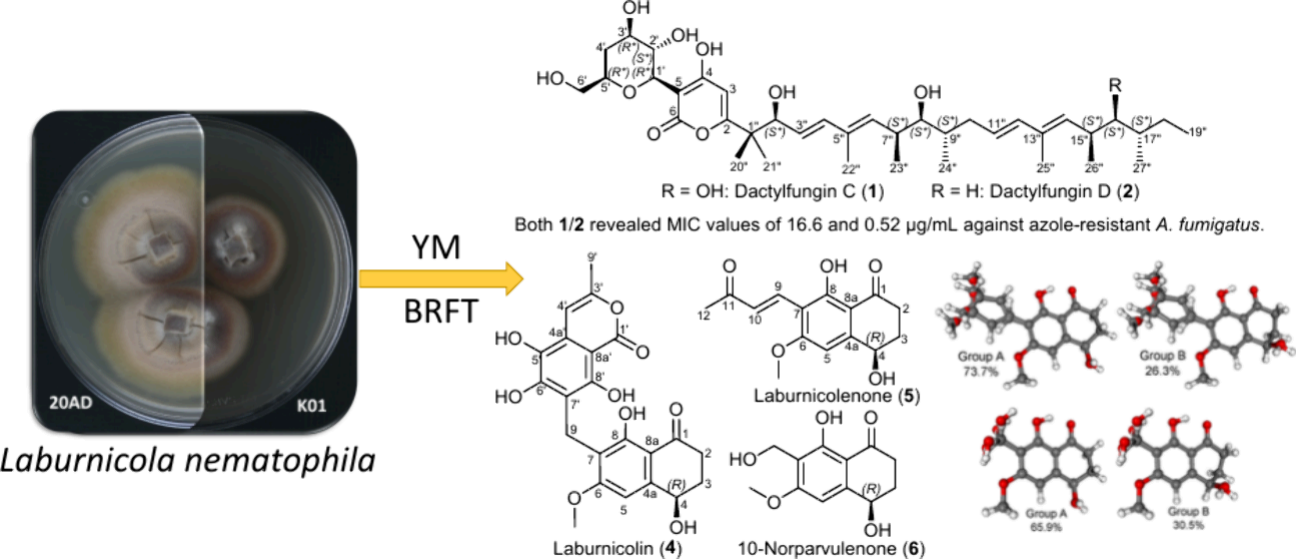

A chemical investigation of *Laburnicola nematophila*, isolated from cysts of the plant parasitic nematode *Heterodera filipjevi*, affored three dactylfungin derivatives (**1**–**3**) and three tetralone congeners (**4**–**6**). Dactylfungin C (**1**), laburnicolin (**4**), and laburnicolenone (**5**) are previously undescribed natural products. Chemical structures of the isolated compounds were determined based on 1D and 2D NMR spectroscopic analyses together with HR-ESI-MS spectrometry and comparison with data reported in the literature. The relative configurations of compounds **1**, **2**, and **4**–**6** were determined based on their ROESY data and analysis of their coupling constants (*J* values). The absolute configurations of **4**–**6** were determined through the comparison of their measured and calculated TDDFT-ECD spectra. Compounds **1**–**3** were active against azole-resistant *Aspergillus fumigatus*.

Fungal natural products have emerged as a prolific source of novel antimicrobial drugs and therapeutic agents.^1−3^ While the challenge of multiresistant bacteria has been extensively acknowledged, the issue of multidrug-resistant pathogenic fungi is frequently overlooked.^4^ This oversight is particularly noteworthy considering the limited availability of compound classes suitable for treating invasive fungal infections.^3^ Recognizing the gravity of the situation, the World Health Organization (WHO) has designated *Cryptococcus neoformans* and *Aspergillus fumigatus* alongside *Candida* spp. in the critical priority group.^5^ Cryptococcosis caused by *C. neoformans*, in particular, is a crucial concern for immunocompromised individuals such as those with an HIV infection.^6^ Similarly, *A. fumigatus* is also a major threat for immunosuppressed patients.^7,8^ The emergence of azole-resistant strains, in particular those belonging to *A. fumigatus*, has become a real threat and underscores the critical need to identify novel drug targets.^6,9^

The genus *Laburnicola* was introduced by Wanasinghe et al.^10^ and belongs to the Pleosporales, the largest within the Dothideomycetes.^11^ The genus shows a broad spectrum of potential hosts and is described as an endophyte and a nematode antagonist.^12^ This bifunctional lifestyle was also reported for *Polyphilus sieberi* and *Polydomus karssenii*, both initially described from infected nematode eggs, which proved to be prolific producers of biologically active secondary metabolites.^13−15^

In this report, we chemically explored brown rice based solid-state and liquid YM 6.3 cultures of *Laburnicola nematophila* 20AD (DSM 112866) and K01 (DSM 112867) strains, isolated from eggs of the cereal cyst nematode *Heterodera filipjevi*.^12^ The present study deals with the evaluation of the secondary metabolites of *L. nematophila*, some of which revealed significant antifungal activity against human pathogenic azole-resistant strains.

## Results and Discussion

### Isolation and Identification of Compounds **1**–**6**

**Figure 1:**
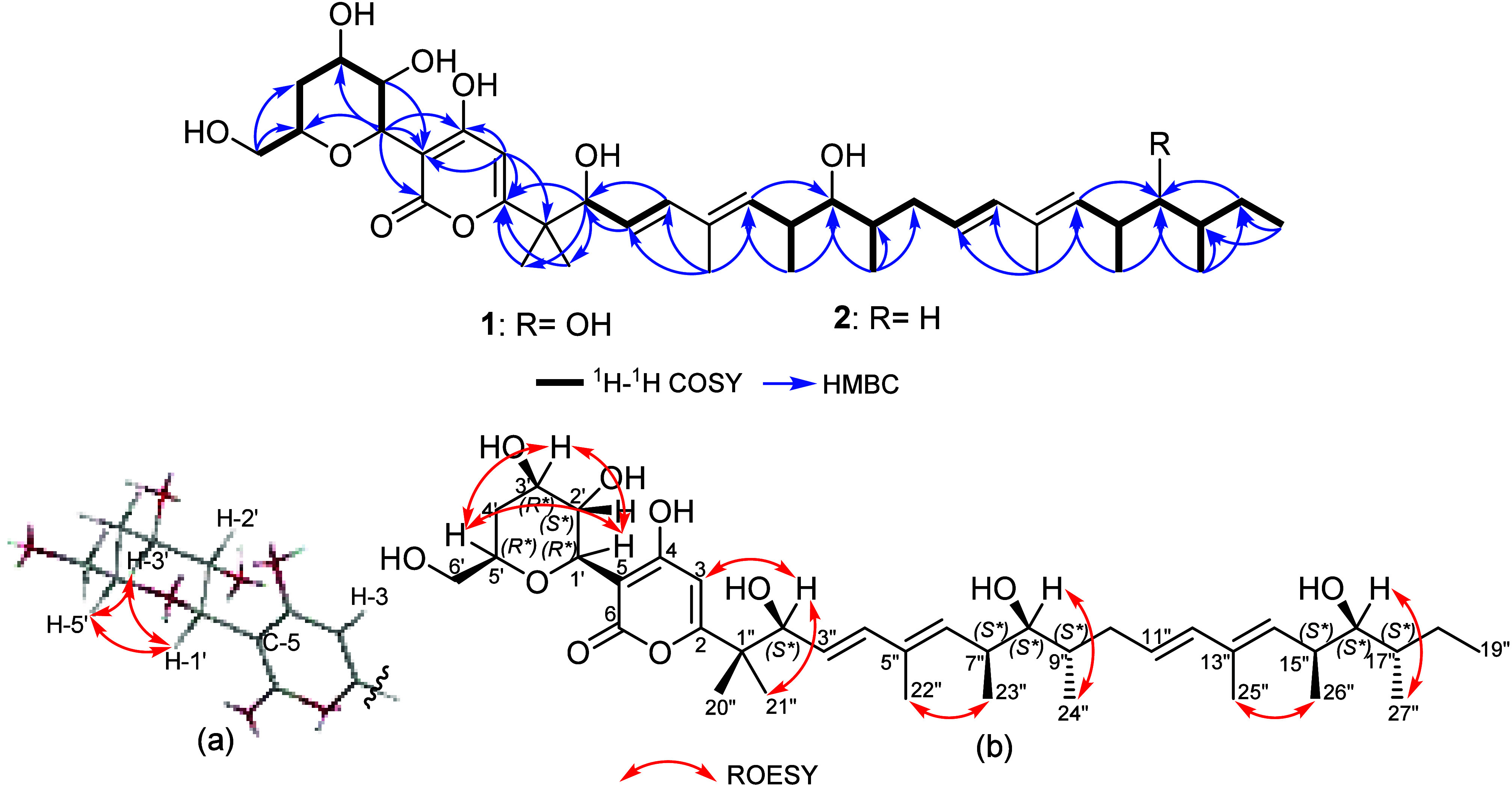
Key ^1^H–^1^H COSY, HMBC, and ROESY correlations of **1** and **2**.

Compound **1** was isolated as a white amorphous solid. HR-ESI-MS determined its molecular formula as C_38_H_60_O_10_, indicating nine degrees of unsaturation. The ^1^H and ^13^C NMR data of **1** (Table 1) revealed a comparable pattern to those values reported for dactylfungin A, an α-pyrone-containing antifungal agent first reported from *Dactylaria parvispora*,^16^ and YM-202204 (**3**), an antifungal antibiotic first reported from the marine-derived fungus *Phoma* sp.^17^ A detailed comparison of their spectral data revealed that, as in dactylfungin A, compound **1** features a polyalcohol residue and a long aliphatic side-chain. The α-pyrone was confirmed via the HMBC spectrum (Figure 1), which revealed key correlations from a singlet olefinic proton at δ_H_ 5.89 (s, H-3; δ_C_ 108.0) to four unprotonated carbon atoms at δ_C_ 44.6 (C-1″), δ_C_ 170.2 (C-2), δ_C_ 180.1 (C-4), and δ_C_ 97.5 (C-5), confirming the depicted structural arrangement for **1** with the long side-chain and the polyalcohol moiety to be attached at C-2 and C-5 of the α-pyrone functionality, respectively. The long side-chain in **1** featured two pairs of olefinic protons at δ_H_ 5.58 (H-3″)/δ_H_ 6.29 (H-4″) and δ_H_ 5.55 (H-11″)/δ_H_ 6.04 (H-12″), as elucidated from the ^1^H NMR (Table 1) and ^1^H–^1^H COSY spectra (Figure 1), which were deduced to be in *trans* configuration based on their large coupling constants (*J* values ≥ 15.0–16.0 Hz).
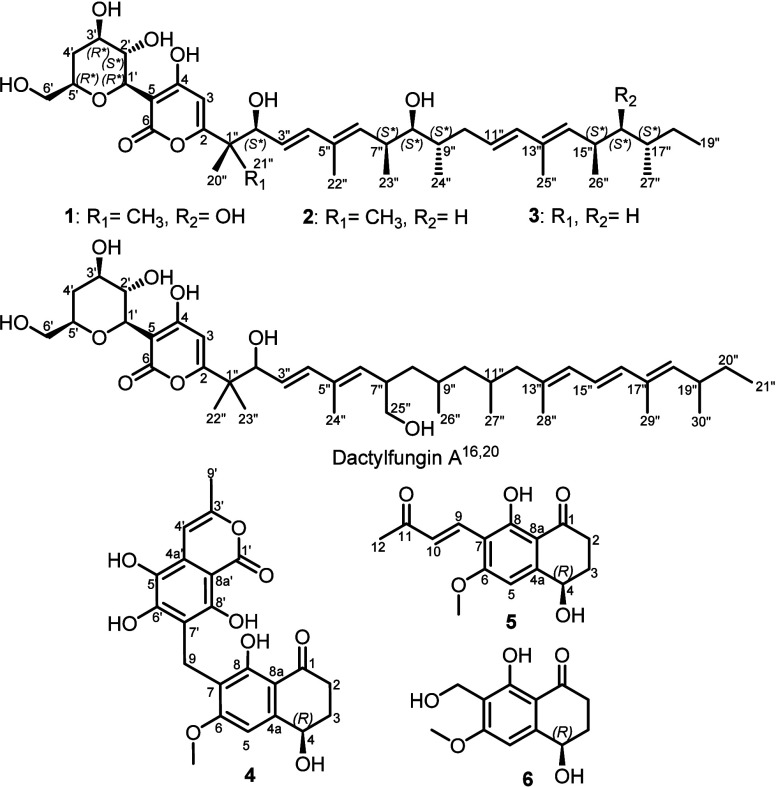


**Table 1:** 1D (^1^H and ^13^C) NMR Data of **1** and **2**

	**1**		**2**	
**pos**.	**δ**_**C**_,^a^^,^^c^**type**	**δ**_**H**_^b^**multi (*****J*****[Hz])**	**δ**_**C**_,^a^^,^^c^**type**	**δ**_**H**_^b^**multi (*****J*****[Hz])**
2	170.2, C		171.3, C	
3	108.0, CH	5.89 s	102.7, CH	6.03 s
4	180.1, CO		173.5, CO	
5	97.5, C		99.7, C	
6	170.0, C		167.8, C	
1′	76.5, CH	4.48 d (9.7)	75.9, CH	4.49 d (9.8)
2′	73.1, CH	4.13 dd (9.7, 8.8)	73.5, CH	4.04 dd (9.8, 8.7)
3′	74.6, CH	3.62 m (overlapped)	74.3, CH	3.62 m (overlapped)
4′	36.1, CH_2_	α 1.68 q (12.9); β 1.91 ddd (12.9, 5.4, 2.0)	36.5, CH_2_	α 1.60 q (12.5); β 1.95 ddd (12.5, 5.1, 1.9)
5′	77.6, CH	3.62 m (overlapped)	78.26, CH	3.62 m (overlapped)
6′	65.5, CH_2_	α 3.55 dd (11.2, 5.4, 4.0); β 3.58 dd (11.2, 5.0)	66.8, CH_2_	α 3.55 dd (11.6, 5.3); β 3.59 dd (11.6, 3.8)
1″	44.6, C		45.3, C	
2″	77.9, CH	4.36 dd (7.4, 1.0)	78.30, CH	4.34 d (7.5)
3″	127.2, CH	5.58 dd (15.7, 7.4)	126.0, CH	5.58 dd (15.6, 7.5)
4″	138.7, CH	6.29 d (15.7)	139.3, CH	6.30 d (15.6)
5″	134.0, C		134.0, C	
6″	135.2, CH	5.56 d (10.0)	135.8, CH	5.58 d (10.5)
7″	36.6, CH	2.75 ddt (10.0, 6.9, 3.7)	36.7, CH	2.76 dqd (10.5, 6.9, 3.7)
8″	80.3, CH	3.18 dd (8.0, 3.7)	80.4, CH	3.19 dd (8.0, 3.7)
9″	38.5, CH	1.51 ddt (11.1, 8.6, 2.9)	38.7, CH	1.51 ddt (11.2, 8.3, 3.1)
10″	36.8, CH_2_	α 2.46 ddd (12.8, 5.3, 3.1); β 1.91 ddd (12.9, 5.4, 2.0)	37.0, CH_2_	α 2.46 ddd (13.6, 5.4, 3.3); β 1.90 dt (13.6, 8.6)
11″	126.3, CH	5.55 dt (15.0, 6.9)	126.8, CH	5.52 ddd (15.4, 8.1, 6.5)
12″	137.6, CH	6.04 d (15.4)	137.9, CH	6.03 d (15.4)
13″	133.5, C		133.3, C	
14″	134.5, CH	5.11 d (10.0)	138.2, CH	5.06 d (9.7)
15″	37.5, CH	2.61 m	31.27, CH	2.60 dtdd (9.7, 6.6, 5.3, 4.7, 2.2)
16″	79.0, CH	3.22 dd (8.8, 2.5)	31.31, CH_2_	α 1.08 tt (11.0, 5.1); β 1.29 ddd (11.0, 8.8, 4.7)
17″	38.8, CH	1.41 m	33.6, CH	1.26 m (overlapped)
18″	28.2, CH_2_	α 1.26 m (overlapped); β 1.42 m (overlapped)	31.31, CH_2_	α 1.15 m (overlapped); β 1.29 m (overlapped)
19″	12.1, CH_3_	0.89 t (7.3)	11.7, CH_3_	0.86 t (7.3)
20″	21.0, CH_3_	1.21 s	20.7, CH_3_	1.22 s
21″	22.4, CH_3_	1.14 s	22.9, CH_3_	1.16 s
22″	12.9, CH_3_	1.76 d (1.3)	13.02, CH_3_	1.77 d (1.3)
23″	18.0, CH_3_	1.02 d (6.6)	18.8, CH_3_	1.02 d (6.9)
24″	16.3, CH_3_	0.81 d (6.8)	16.4, CH_3_	0.81 d (6.8)
25″	12.9, CH_3_	1.75 d (1.2)	12.99, CH_3_	1.73 d (1.2)
26″	18.6, CH_3_	1.01 d (6.9)	22.1, CH_3_	0.93 d (6.6)
27″	12.8, CH_3_	0.80 d (6.6)	19.5, CH_3_	0.84 d (6.3)

a Measured in methanol-*d*_4_ at 175 (for ^13^C) MHz.

b Measured in methanol-*d*_4_ at 700 (for ^1^H) MHz.

c Assignment confirmed by HMBC and HSQC spectra.

The ^1^H–^1^H COSY correlations of **1** (Figure 1) revealed an extended spin system over H-6″/H-7″/H-8″/H-9″/H_2_-10″/H-11″/H-12″ in addition to a second spin system on the side-chain over H-14″/H-15″/H-16″/H-17″/H_2_-18″/H_3_-19″. The ^13^C NMR and HSQC spectral data of **1** (Table 1, Figure S7) revealed the presence of seven methine sp^3^ carbon atoms, four belonging to the polyalcohol moiety at δ_C_ 77.6 (C-5′), δ_C_ 76.5 (C-1′), δ_C_ 74.6 (C-3′), and δ_C_ 73.1 (C-2′), resembling those in dactylfungin A,^16^ and the remaining three methine carbon atoms at δ_C_ 80.3, δ_C_ 79.0, and δ_C_ 77.9 ascribed to C-8″, C-16″, and C-2″ on the long side-chain, respectively. Further confirmation of the suggested positions of the three methines on the long side-chain in **1** was afforded by its HMBC and HSQC spectra (Figures S6–S7). The HMBC correlation of **1** (Figure 1) revealed key correlations from a methine proton at δ_H_ 4.36 (dd, *J* = 7.3, 1.0 Hz; δ_C_ 77.9) to δ_C_ 170.2 (C-2), δ_C_ 127.2 (C-3″), δ_C_ 138.7 (C-4″), and diastereotopic methyl groups at δ_C_ 21.0 (C-20″)/δ_C_ 22.4 (C-21″). These correlations confirmed its position to be at C-2″. The second methine sp^3^ carbon at δ_C_ 80.3 was correlated via key HMBC correlations (Figure 1) to two doublet methyl groups at δ_H_ 1.02 (d, *J* = 6.6 Hz; δ_C_ 18.0) and δ_H_ 0.81 (d, *J* = 6.8 Hz; δ_C_ 16.3) assigned to H_3_-23″ and H_3_-24″, respectively, indicating the presence of this methine carbon at C-8″. Similarly, the last methine sp^3^ carbon at δ_C_ 79.0 (δ_H_ 3.22, dd, *J* = 8.8, 2.5 Hz) was assigned to be at C-16″ based on the HMBC spectrum (Figures 1, S6), which revealed key correlations to C-16″ from two doublet methyl groups at δ_H_ 1.01 (d, *J* = 6.9 Hz, H_3_-26″; δ_C_ 18.6) and δ_H_ 0.80 (d, *J* = 6.6 Hz, H_3_-27″; δ_C_ 12.8) in addition to diastereotopic methylene protons at δ_H_ 1.26/δ_H_ 1.42 (H_2_-18″; δ_C_ 28.2). The latter was correlated via the ^1^H–^1^H COSY spectrum (Figure 1) to a terminal triplet methyl group at δ_H_ 0.89 (t, *J* = 7.3 Hz, H_3_-19″).

The relative configuration of the polyalcohol substructure in **1** (Figure 1a) was determined by the analysis of the coupling constants (*J* values) of H-1′ at δ_H_ 4.48 (d, *J* = 9.7 Hz) and H-2′ at δ_H_ 4.13 (dd, *J* = 9.7, 8.8 Hz), which assigned both in axial orientation. The ROE correlations revealed by the polyalcohol substructure (Figures 1, S8) from H-1′ to H-3′ and H-5′ indicated that they are cofacial and hence similarly adopting axial orientations. The relative configuration at C-2″, C-8″, and C-16″ could be deduced based on the analysis of the coupling constant values of H-2″ at δ_H_ 4.36 (dd, *J* = 7.3, 1.0 Hz), H-8″ at δ_H_ 3.18 (dd, *J* = 8.0, 3.7 Hz), and H-16″ at δ_H_ 3.22 (dd, *J* = 8.8, 2.5 Hz) and by the ROESY correlations between H-2″/H_3_-21″, H-8″/H_3_-24″, and H-16″/H_3_-27″, indicating their orientation toward the same face of the molecule, whereas H_3_-20″, H_3_-23″, and H_3_-26″ are directed toward the opposite side. These results were further compared to the reported literature of super-carbon-chain-compounds (SCCCs) such as gibbols A/B^18^ and benthol A.^19^

These results suggested the relative configuration of **1** as (1′R*,2′S*,3′R*,5′R*,2″S*,7″S*,8″S*,9″S*,15″S*,16″S*,17″S*). To unambiguously determine the absolute configuration of the chiral centers on the side-chain in **1**, it necessitates its cleavage into shorter fragments by ozonolysis; the fragments would then be subjected to Mosher’s ester derivatization as previously described for other SCCCs.^18,19^ Due to the low amount of **1** (0.8 mg) obtained, this scheme was not possible to implement, and hence its absolute configuration was not determined. Based on the results obtained and by comparison with the reported spectral data of dactylfungins,^16,20^ compound **1** was identified as a previously undescribed derivative that was trivially named dactylfungin C.

Compound **2** was obtained as a yellow amorphous solid. Its molecular formula was determined to be C_38_H_60_O_9_ based on its HR-ESI-MS data, indicating nine degrees of unsaturation similar to **1**. Based on the molecular formulas, compound **2** was suggested to be a deoxygenated derivative of **1**. Expectedly, both ^1^H and ^13^C NMR data of **1** and **2** (Table 1) revealed a close coherence apart from one main difference due to the disappearance of one methine sp^3^ carbon at δ_C_ 79.0 (C-16″) and the presence of a secondary sp^3^ carbon at δ_C_ 31.31 (C-16″) that was correlated via the HSQC spectrum (Figure S15) to diastereotopic methylene protons at δ_H_ 1.08/δ_H_ 1.29 ppm. In the ^1^H–^1^H COSY spectrum (Figures 1, S13), the previous diastereotopic methylene protons featured a spin-system extending over one aliphatic methine proton at δ_H_ 1.26 (m, H-17″) to a second diastereotopic methylene group at δ_H_ 1.15/δ_H_ 1.29 (H_2_-18″) ending with a terminal triplet methyl group at δ_H_ 0.86 (t, *J* = 7.3 Hz, H_3_-19″). The results obtained suggest that compound **2** is a 16-deoxy derivative of **1**. A literature search of **2** revealed that a related derivative was patented in 2005 as a synthetic anti-inflammatory lactone.^21^ Further confirmation of the structure of **2** was obtained via 2D NMR spectra including ^1^H–^1^H COSY and HMBC (Figure 1) spectra, which revealed similar correlations to those shown for **1**. Similar to **1**, the relative configuration at the chiral centers in **2** on both the polyalcohol residue (Figure 1a) and the aliphatic side-chain was determined based on the analysis of the coupling constants (*J* values) and the ROESY spectrum (Figures 1, S16) taking into consideration a common biosynthetic origin. Accordingly, the relative configuration of **2** was suggested to be (1′R*,2′S*,3′R*,5′R*,2″S*,7″S*,8″S*,9″S*,15″R*,17″S*). The absolute configuration of **2** necessitates applying the scheme previously described,^18,19^ which was not possible due to the limited amount obtained (1.3 mg). According to the obtained spectral data of **2**, it was identified as a 16-deoxy derivative of **1** and it was given the trivial name dactylfungin D.

**Figure 2:**
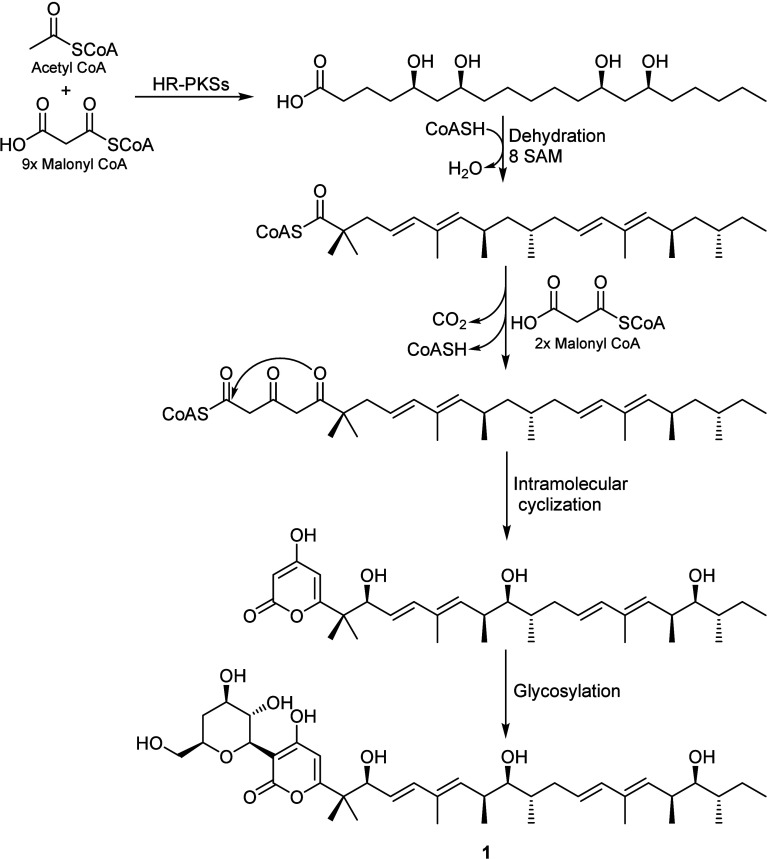
A plausible biosynthetic pathway of **1**.

Chemically, dactylfungins feature a 4-hydroxy-α-pyrone core structure with an elongated side-chain and C-glycosidic substituents at C-2 and C-5, respectively. Accordingly, their biosynthesis (Figure 2) was proposed to be through an acetate malonate pathway catalyzed by highly reducing polyketide synthases (HR-PKSs) starting with an acetyl-CoA with 11 malonyl-CoA units in two phases including dehydration and methylation steps. Thereafter, the 4-hydroxy-α-pyrone moiety was biosynthesized through an intramolecular cyclization followed by a glycosylation at C-5 that ultimately afforded dactylfungin C (**1**).

**Figure 3:**
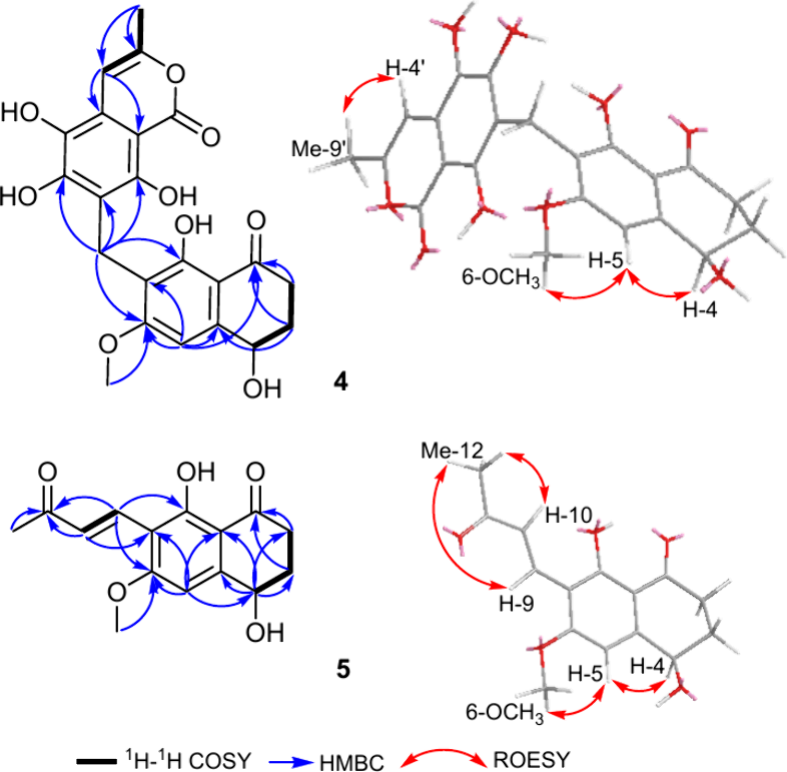
Key ^1^H–^1^H COSY, HMBC, and ROESY correlations of **4** and **5**.

Compound **4** was purified as a brown amorphous solid. HR-ESI-MS data established a molecular formula of C_22_H_20_O_9_, thus indicating 13 degrees of unsaturation. The ^13^C NMR spectral data of **4** (Table 2, Figure S26) revealed 22 carbon resonances that were differentiated into 14 unprotonated sp^2^ carbon atoms including one ketocarbonyl (δ_C_ 203.3), one carbonyl (δ_C_ 166.5), six oxygenated olefinic carbon atoms (δ_C_ 163.8, 161.5, 154.9, 154.4, 151.6, 151.5), and six olefinic carbon atoms (δ_C_ 148.3, 129.9, 113.3, 113.1, 109.3, 95.6). In addition, the ^13^C NMR and HSQC spectra of **4** (Table 2, Figure S29) exhibited the presence of two methine sp^2^ carbon atoms at δ_C_ 100.8 and 99.3 that were directly correlated to two olefinic protons at δ_H_ 6.71 (d, *J* = 0.9 Hz) and 6.58 (d, *J* = 1.2 Hz) along with one oxygenated methine sp^3^ carbon at δ_C_ 66.4 directly correlated to a proton resonance at δ_H_ 4.68 (dd, *J* = 9.2, 4.3 Hz). Three secondary methylene carbon atoms (δ_C_ 35.1, 31.7, 16.7) and two primary methyl carbon atoms, one oxygenated at δ_C_ 55.8 (δ_H_ 3.77, s) and one olefinic at δ_C_ 19.0 (δ_H_ 2.20, d, *J* = 1.2 Hz), were also recognized. A literature search of **4** suggested its identity as a heterodimeric structure comprising a tetralone moiety similar to 10-norparvulenone (**6**)^22^ together with a 5,6,8-trihydroxyisocoumarin as in peniisocoumarins.^23−25^ The ^1^H–^1^H COSY spectrum of **4** (Figure 3) revealed a spin system similar to that from the 10-norparvulenone moiety extending from an oxygenated methine at δ_H_ 4.68 (dd, *J* = 9.2, 4.3 Hz, H-4) to a diastereotopic methylene group at δ_H_ 1.90/δ_H_ 2.14 (H_2_-3) and to a second methylene group at δ_H_ 2.66 (t, *J* = 7.4 Hz, H_2_-2). Moreover, a long-range ^4^*J*_HH_ coupling was noticed in the isocoumarin subunit between an olefinic proton at δ_H_ 6.58 (d, *J* = 1.2 Hz, H-4′) and an olefinic methyl group at δ_H_ 2.20 (d, *J* = 1.2 Hz, H_3_-9′), indicating the presence of the olefinic methyl group at C-3′. Further confirmation of the depicted structure of **4** was provided by the HMBC spectrum (Figures 3, S28), which revealed key correlations from a deshielded methylene group at δ_H_ 3.88 (δ_C_ 16.7, C-9) to five different carbon atoms at δ_C_ 163.8 (C-6), 161.5 (C-8), 154.9 (C-8′), 154.4 (C-6′), and 113.1 (C-7′), assigning it as a bridging moiety between the two building units of the molecule. In addition, the HMBC spectrum (Figures 3, S28) featured a key correlation from a methoxy group at δ_H_ 3.77 (s) to C-6 (δ_C_ 163.8), where it is positioned.

**Table 2:** ^1^H and ^13^C NMR Data of **4** and **5**

	**4**		**5**	
**pos**.	**δ**_**C**_,^a^^,^^c^**type**	**δ**_**H**_^b^**multi (*****J*****[Hz])**	**δ**_**C**_,^c^^,^^d^**type**	**δ**_**H**_^e^**multi (*****J*****[Hz])**
1	203.3, CO		204.7, CO	
2	35.1, CH_2_	2.66 t (7.4)	36.2, CH_2_	α 2.71 ddd (17.9, 10.7, 4.9); β 2.83 ddd (17.9, 5.8, 4.6)
3	31.7, CH_2_	α 1.90 dtd (12.5, 9.2, 6.2); β 2.14 dd (12.5, 4.3)	32.5, CH_2_	α 2.06 dddd (12.9, 10.8, 9.4, 4.6); β 2.31 dd (12.9, 5.1)
4	66.4, CH	4.68 dd (9.2, 4.3)	68.6, CH	4.83 dd (9.4, 4.1)
4a	148.3, C		153.6, C	
5	100.8, CH	6.71 d (0.9)	101.9, CH	6.89 s
6	163.8, C		166.5, C	
7	113.3, C		110.7, C	
8	161.5, C		165.6, C	
8a	109.3, C		111.0, C	
9	16.7, CH_2_	3.88 s	135.1, CH	7.98 d (16.5)
10			130.6, CH	7.25 d (16.5)
11			202.8, CO	
12			27.3, CH_3_	2.35 s
6-OCH_3_	55.8, CH_3_	3.77 s	56.9, CH_3_	4.04 s
4-OH		5.59 br s		
8-OH		13.02 br s		
1′	166.5, CO			
3′	151.5, C			
4′	99.3, CH	6.58 d (1.2)		
4a′	129.9, C			
5′	151.6, C			
6′	154.4, C			
7′	113.1, C			
8′	154.9, C			
8a′	95.6, C			
9′	19.0, CH_3_	2.20 d (1.2)		
8′-OH		10.97 s		

a Measured in DMSO-*d*_6_ at 125 (for ^13^C) MHz.

b Measured in DMSO-*d*_6_ at 500 (for ^1^H) MHz.

c Assignment confirmed by HMBC and HSQC spectra.

d Measured in methanol-*d*_4_ at 125 MHz

e Measured in methanol-*d*_4_ at 500 MHz.

The ROESY spectrum of **4** (Figures 3, S30) further confirmed the depicted structure by revealing key ROE correlations from both H-4 and OCH_3_-6 to the aromatic proton at δ_H_ 6.71 (d, *J* = 0.9 Hz) assigned to H-5. The absolute configuration of the carbinol group at C-4 was predicted to be (*R*) based on biosynthetic considerations and the assigned absolute configurations of the co-isolated compounds **5** and **6**. When comparing the experimental ECD spectra of **4**, **5**, and **6**, it was evident that **4** has a much smaller intensity due to the presence of two interacting similar aromatic chromophores connected with a methylene linker, where the different conformer groups with comparable populations can cancel the ECD contributions of one another.^26^ The Boltzmann distribution has a fundamental effect on the spectrum that can be challenging to estimate precisely due to the known limitations of DFT functionals.^27^ According to the obtained results and by comparison to the reported literature,^22−25^ compound **4** was identified as a previously undescribed heterodimer compound and was named laburnicolin.

**Figure 4:**
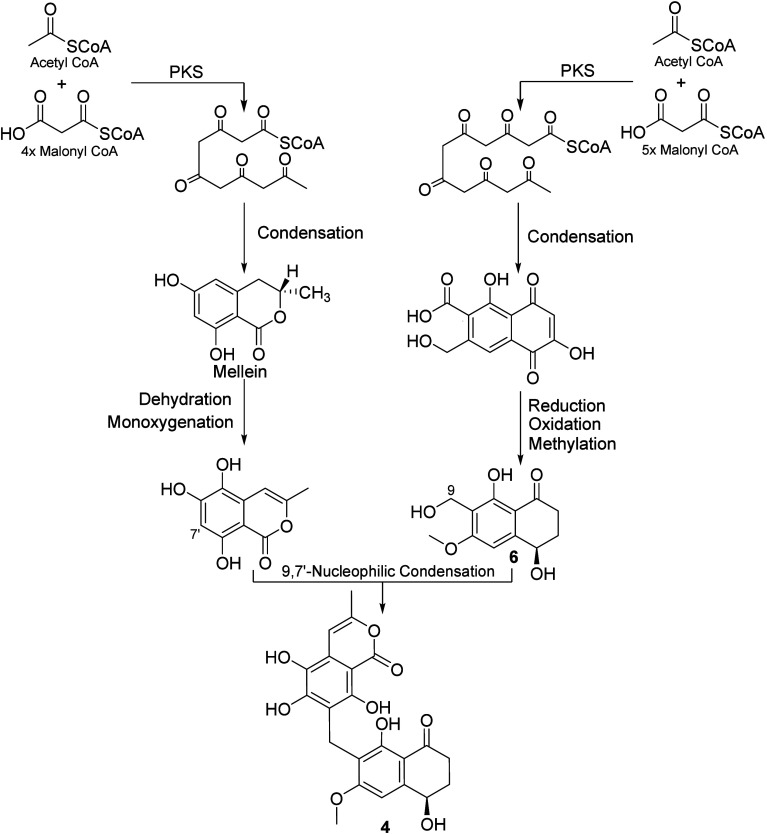
A plausible biosynthetic pathway of **4** and **6**.

Being a heterodimer composed of an isocoumarin and a tetralone subunit, compound **4** was probably biosynthesized through an acetate–malonate pathway influenced by polyketide synthase (PKS). A plausible biosynthetic scheme of laburnicolin (**4**) (Figure 4) suggests that its biosynthesis is via two parallel phases of a PKS pathway to afford the building subunits 10-norparvulenone (**6**) and 2-methyl-5,6,8-trihydroxyisocoumarin, which would be finally combined via nucleophilic condensation to yield laburnicolin (**4**).

Compound **5** was isolated as a white amorphous solid. Its molecular formula was determined to be C_15_H_16_O_5_ based on its HR-ESI-MS data, indicating eight degrees of unsaturation. The ^1^H and ^13^C NMR spectral data of **5** (Table 2) revealed comparable resonance values to those measured and reported for 10-norparvulenone (**6**).^22^ A detailed investigation of the ^1^H and ^13^C NMR data of **5** (Table 2) revealed the presence of a second ketocarbonyl carbon at δ_C_ 202.8 (C-11) and two olefinic carbon atoms at δ_C_ 135.1 (C-9) and δ_C_ 130.6 (C-10) directly correlated via HSQC to two olefinic protons at δ_H_ 7.98 and δ_H_ 7.25 appearing as doublet signals with a coupling constant of 16.5 Hz, indicating a *trans* configuration. In addition, the ^1^H NMR spectrum of **1** (Table 2) revealed a singlet methyl group at δ_H_ 2.35 (H_3_-12) that exhibited together with H-9 and H-10 common key HMBC correlations (Figure 3) to the ketocarbonyl carbon assigned to C-11. By comparing the obtained ^1^H/^13^C NMR data of **5** (Table 2) and 10-norparvulenone (**6**) (Li et al., 2010),^22^ it was concluded that **5** features an α,β-unsaturated ketone moiety replacing the hydroxymethylene functionality in 10-norparvulenone (**6**).

**Figure 5:**
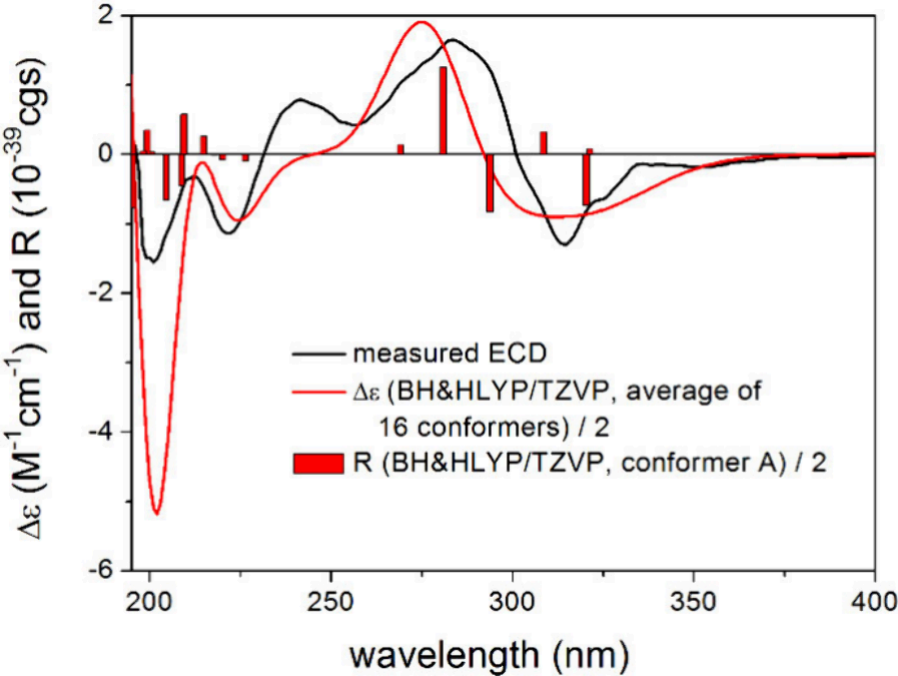
Experimental ECD spectrum of **5** in MeOH compared with the BH&HLYP/TZVP PCM/MeOH ECD spectrum of (*R*)-**5** computed for the low-energy ωB97X/TZVP PCM/MeOH conformers. The bars represent the rotational strength values of the lowest-energy conformer.

To confirm the position of the α,β-unsaturated ketone moiety on the tetralone core, its HMBC spectrum (Figure 3) revealed further key correlations from H-10 and H-5 (δ_H_ 6.89, s) to an unprotonated olefinic carbon at δ_C_ 110.7 (C-7), whereas other key HMBC correlations were recognized from H-9 to two oxygenated aromatic carbons at δ_C_ 165.6 (C-8) and 166.5 (C-6). These key HMBC correlations confirmed the binding of the α,β-unsaturated ketone moiety at C-7 in **5**. The ROESY spectrum of **5** (Figure 3) revealed similar key ROE correlations to those exhibited by **4** from both H-4 (δ_H_ 4.83, dd, *J* = 9.4, 4.1 Hz) and OCH_3_-6 (δ_H_ 4.04, s) to the aromatic proton at δ_H_ 6.89 assigned to H-5. To determine the absolute configuration, the TDDFT-ECD method was applied on the (*R*) enantiomer of **5**.^28,29^ The Merck molecular force field (MMFF) conformational search yielded 57 conformer clusters in a 21 kJ mol^–1^ energy window, the ωB97X/TZVP PCM/MeOH reoptimization of which resulted in 16 low-energy conformers over 1% Boltzmann population. The computed ECD spectra at various levels of theory gave a good agreement with the experimental ECD spectrum, underestimating only the positive shoulder at 241 nm, and it suggested the (*R*) absolute configuration (Figure 5).

**Figure 6:**
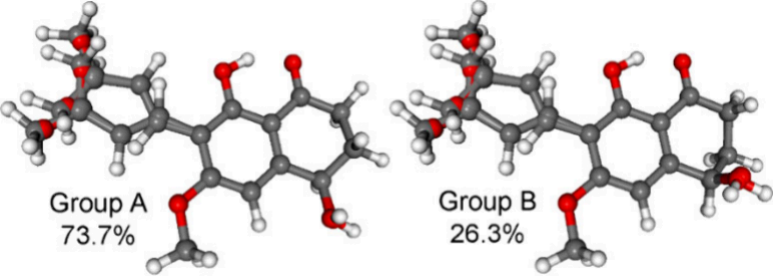
Overlapped geometries for two groups of the low-energy ωB97X/TZVP PCM/MeOH conformers of (*R*)-**5**: group A with *M* helicity and an *equatorial* hydroxy group represented by conformers A–H, and group B with *P* helicity and an *axial* hydroxy group represented by conformers I–P.

For tetralones, a semiempirical ECD helicity rule^30,31^ may be applied to correlate the n−π* transition of the conjugating carbonyl group above 300 nm with the helicity of the carbocyclic ring and hence the absolute configuration. According to the literature data, some substituted tetralones with *M* helicity of the fused carbocyclic ring produced a positive n−π* CE (Cotton effect),^32−35^ while others with *P* helicity also showed positive n−π* CEs.^36,37^ The computed conformers of (*R*)-**5** were classified into two groups (Figure 6).

On the basis of the *P* and *M* helicity of the carbocyclic ring, which gave nearly opposite ECD spectra (Figure S53) and as suggested by the Kohn–Sham orbitals, the carbonyl n−π* CE was identified as the fourth transition with negative sign, which was derived from *M* helicity of the high population conformers (Figure S39). Furthermore, in the experimental ECD spectrum there is only a slightly visible shoulder at 303 nm corresponding to this transition that makes application of the helicity rule ambiguous for similar derivatives. Based on the obtained results, compound **5** was identified as a previously undescribed tetralone derivative, and it was given the trivial name laburnicolenone.

**Figure 7:**
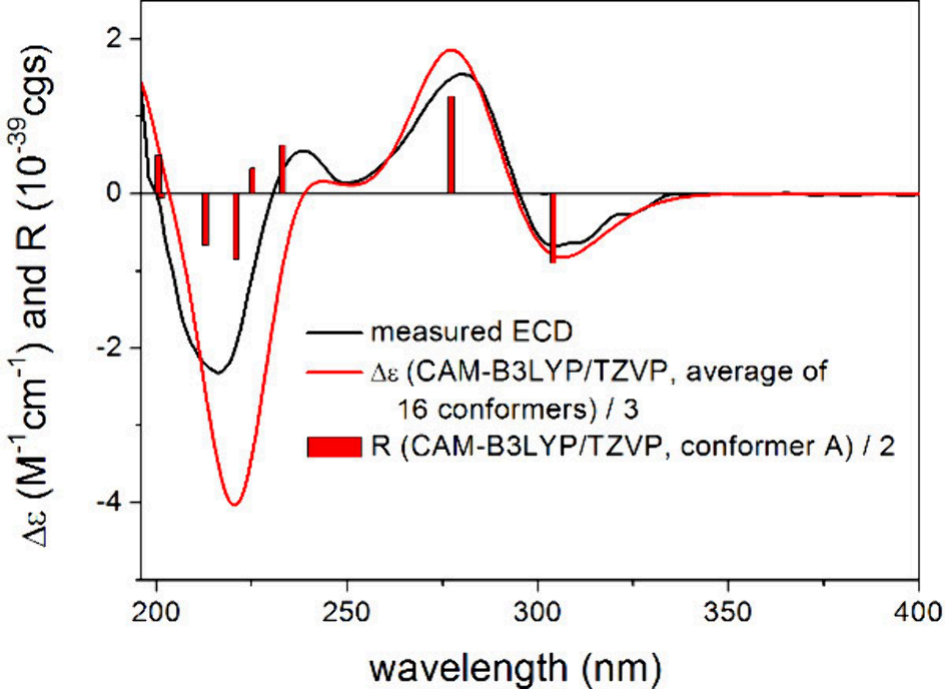
Experimental ECD spectrum of **6** measured in MeOH compared with the CAM-B3LYP/TZVP PCM/MeOH ECD spectrum of (*R*)-**6** computed for the low-energy ωB97X/TZVP PCM/MeOH conformers. The bars represent the rotational strength values of the lowest-energy conformer.

**Figure 8:**
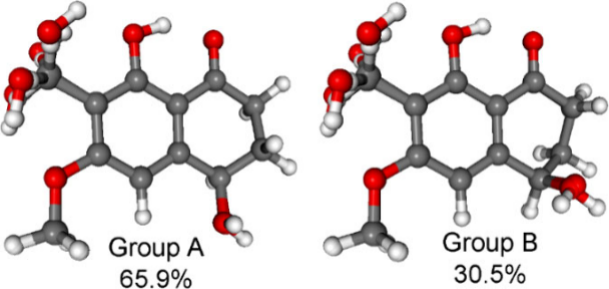
Two groups of the low-energy ωB97X/TZVP PCM/MeOH conformers of (*R*)-**6**: group A with *M* helicity and an *equatorial* hydroxy group represented by conformers A–H, and group B with *P* helicity and an *axial* hydroxy group represented by conformers I–P.

In the literature, the (*R*) absolute configuration was reported for **6** on the basis of the opposite specific optical rotation to that of *O*-methylasparvenone.^38^ Although structurally related derivatives often exhibit the same sign of optical rotation, it is known that even slight structural modifications in the substitution pattern may invert the sign of the specific optical rotation.^39,40^ In order to elucidate the absolute configuration of **6** independently, ECD calculations were carried out. DFT reoptimization of the 33 initial MMFF conformers of (*R*)-**6** resulted in 16 low-energy conformers, the ECD calculations of which were performed at various levels of theory, affording excellent agreement with the experimental ECD spectrum (Figure 7). Similarly to **5**, the low-energy computed conformers of **6** could be classified into two groups with nearly mirror-image ECD spectra and opposite helicity, where the first group with *M* helicity was more abundant than the second with *P* helicity (Figures 8 and S54).

By checking the Kohn–Sham orbitals (Figure S52), the n−π* transition was identified as the second ECD transition with a positive sign, which appeared in the experimental ECD spectrum (Figure 7) below 300 nm as the second CE. This can be a source of confusion when the semiempirical tetralone helicity rule would be applied for similar scaffolds, and thus ECD calculations are recommended. The TDDFT-ECD calculations confirmed that the carbocyclic ring adopted preferably *M* helicity in both (*R*)-**5** and (*R*)-**6** with an equatorial 4-OH group, while their n−π* CE was found to have opposite signs: negative for **5** and positive for **6**.

### Biological Assays

The effects of all isolated metabolites are shown in Table 3 (complete data set in Table S1). Besides the assessment of antimicrobial and cytotoxic activities, dactylfungins (**1**–**3**) were evaluated in an in-depth assay for their antifungal activity against different strains of *Aspergillus fumigatus* including azole-resistant strains, as well as *Cryptococcus neoformans* and *Mucor plumbeus*. The evaluation demonstrated significant antifungal activity for all three substances (**1**–**3**). Dactylfungin D (**2**) showed overall the highest potency against the tested organisms, especially against *A. fumigatus* as well as azole-resistant strains and moderate activity against *C. neoformans* and *Rhodotorula glutinis*. YM-202204 (**3**) revealed activity across multiple fungal species including higher activity against *A. fumigatus* (azole-resistant) and moderate activities against *C. albicans* and *A. fumigatus*, while its highest activity was against *M. plumbeus*. Dactylfungin C (**1**) displayed high impact only on the growth of *A. fumigatus* and weak activity on its azole-resistant strain. Apart from their weak cytotoxic effects, compounds **4** and **5** revealed no other biological activity in the conducted assays, whereas 10-norparvulelone (**6**) illustrated no activity against the tested organisms or cell lines. None of the tested compounds (**1**, **2**, **4**–**6**) exhibited significant activity against *Caenorhabditis elegans* (Table S2). In this study, dactylfungin derivatives (**1**–**3**), featuring an α-pyrone motif substituted with a polyacohol and a long aliphatic side-chain, were interesting with regard to the impact of side-chain substitution pattern on their antifungal activities. In particular, the higher hydrophilicity attained by introducing an additional hydroxy group in **1** compared to **2** seems to negatively affect its antifungal activity especially against azole-resistant *A. fumigatus* strains. This observation is supported by the study of Charria-Girón et al.,^20^ which showed a decreased efficacy of hydroxylated derivative 21″-hydroxy-dactylfungin A compared to dactylfungin A produced by *Amesia hispanica*.

**Table 3:** Antimicrobial Activity (MIC) of **1**–**6**^a^

**Test Microorganism**		**MIC (μg/mL)**						**Positive Control (μg/mL)**
*Staphylococcus aureus*	DSM 346	–	66.6	n.d.	–	–	–	0.21^G^
*Aspergillus fumigatus*	ATCC 204305	0.26	0.52	8.32	n.d.	n.d.	n.d.	0.31^A^
*A. fumigatus*	CCF 3522	n.d.	0.52	n.d.	n.d.	n.d.	n.d.	0.31^A^
*A. fumigatus* (azole-resistant)	CCF 6651	–	2.08	0.52	n.d.	n.d.	n.d.	0.16^A^
*A. fumigatus* (azole-resistant)	CCF 6674	16.6	0.52	0.52	n.d.	n.d.	n.d.	2.5^A^
*Candida albicans*	CCM 8215	–	–	8.3	n.d.	n.d.	n.d.	1.25^A^
*Cryptococcus neoformans*	CCF 1081	–	4.15	4.15	n.d.	n.d.	n.d.	2.5^A^
*Mucor plumbeus*	CCF 2612	–	–	1.04	n.d.	n.d.	n.d.	0.31^A^
*Rhodotorula glutinis*	DSM 10134	–	8.3	n.d.	–	–	–	4.20^N^

a (−): no inhibition up to 67 μg mL^–1^. n.d.: not determined. A: Amphotericin B; G: Gentamicin; N: Nystatin.

*Laburnicola nematophila*, a member of the family Didymosphaeriaceae within the Massarineae, exhibits a fascinating parasitic interaction with nematode eggs.^12,41^ This study identified *L. nematophila* as a novel producer of dactylfungins, potent antifungal agents that strongly inhibit the growth of *A. fumigatus* including azole-resistant strains. The hydroxylation pattern of the hydrophobic aliphatic side-chain significantly affected their activities. The described tetralones showed no biological activity other than their weak cytotoxic effects. Further research investigating their metabolism and interaction in their ecosystems could improve the knowledge of their corresponding ecological roles and would allow applications as an antibiotic producer or as a biocontrol agent.

## Experimental Section

### General Experimental Procedures

For HPLC-DAD-MS analysis, an Amazon Speed ETD ion trap mass spectrometer (Bruker Daltonics, Bremen, Germany) in positive and negative ionization modes was used. As a stationary phase, a C_18_ Acquity UPLC BEH column (50 × 2.1 mm, 1.7 μm Waters, MA, USA) connected to the HPLC system (Dionex UltiMate 3000 UHPLC, Thermo Scientific Inc., Waltham, MA, USA) was utilized. Analysis was performed applying the following conditions: solvent A (deionized H_2_O + 0.1% formic acid (FA)), solvent B (acetonitrile (MeCN) + 0.1% FA), gradient: starting at 5% B for 0.5 min increasing to 100% B in 19.5 min then holding at 100% B for 5 min, flow rate 0.6 mL min^–1^, UV–vis detection 190–600 nm. HR-ESI-MS analyses were performed on a maXis ESI-TOF (Time-Of-Flight) mass spectrometer (Bruker Daltonics, Bremen, Germany) combined with an Agilent 1200 Infinity Series HPLC-UV system (Agilent Technologies, Santa Clara, CA, USA) using the same column and separation gradient as for the HPLC-DAD-MS analysis. Additional parameters: scan range 100–2500 *m*/*z*, rate 2 Hz, capillary voltage 4500 V, dry temperature 200 °C. Compounds were dissolved in deuterated methanol-*d*_4_ or DMSO-*d*_6_, and NMR spectra were recorded on a Bruker Avance III 500 MHz spectrometer equipped with a BBGO (Plus) Smartprobe (^1^H: 500 MHz; ^13^C: 125 MHz) and a Bruker Avance III 700 MHz spectrometer utilizing a 5 mm TCI cryoprobe (^1^H: 700 MHz; ^13^C: 175 MHz). UV–vis spectra were recorded with a Shimadzu UV–vis UV-2450 spectrophotometer (Shimadzu, Kyoto, Japan). Optical rotation and ECD spectra were measured using an Anton Paar MCP-150 polarimeter (Anton Paar, Graz, Austria) at 20 °C and Jasco J-815 spectropolarimeter (Jasco, Pfungstadt, Germany), respectively.

### Fungal Material and Identification

The strains of *Laburnicola nematophila* 20AD (DSM 112866) and K01 (DSM 112867) were isolated from the eggs of the cereal cyst nematode *Heterodera filipjevi* collected in agricultural fields in Yozgat, Turkey.^12^ Molecular phylogenies using combined sequence data were conducted. The acquired sequences were registered under the following GenBank accession numbers: ITS = ON870561, *LSU* = ON870570, *SSU* = ON876674, and *TEF1 =* ON892836 for *L. nematophila* 20AD.^12^ The sequences of *L. nematophila* K01 were registered under the following GenBank accession numbers: ITS = ON870562, *LSU* = ON870571, *SSU* = ON876675, and *TEF1 =* ON892837.^12^ The isolates were maintained on YM 6.3 agar (d-glucose 4 g L^–1^, malt extract 10 g L^–1^, yeast extract 4 g L^–1^, agar 20 g L^–1^, adjusted to pH 6.3, before autoclaving) in the dark.

### Cultivation and Metabolite Extraction

The seed cultures, containing 200 mL of Q6/2 medium (d-glucose 2.5 g L^–1^, glycerol 10 g L^–1^, cottonseed flour 5 g L^–1^, pH 7.2) in a 500 mL Erlenmeyer flask, were inoculated with 5 × 25 mm^2^ sections of mycelium grown on YM 6.3 agar and cultivated at 23 °C and shaking at 140 rpm in the dark. After reaching sufficient biomass, the culture broth was homogenized using an Ultra-Turrax (T25 easy clean digital, IKA) equipped with an S25 N-25F dispersing tool at 10,000 rpm for 10 s. This seed culture served as the inoculum for subsequent cultivations in BRFT (K_2_HPO_4_: 0.5 g L^–1^; sodium tartrate: 0.5 g L^–1^; yeast extract: 1 g L^–1^; 100 mL of solution was added to 28 g of brown rice and autoclaved) and inoculated with 6 mL of the YM 6.3 media seed culture.

### Solid-State Fermentation

For the strain 20AD (DSM 112866), 20 Erlenmeyer flasks containing BRFT media were prepared and inoculated with 6 mL of homogenized seed culture each. Subsequently, 12 flasks were cultivated for 4 weeks, and 8 flasks were cultivated for 6 weeks in the dark at room temperature. After incubation, each culture flask was harvested by adding 3 × 250 mL of acetone, mixed, and extracted, following the previously described protocol.^15^ The total extracts were then defatted by liquid–liquid fractionation between *n*-heptane and methanol. Both fractions (*n*-heptane and methanol) were evaporated to dryness and analyzed with HPLC-DAD-MS. Thirty-five and 15 flasks were prepared for strain K01 (DSM 112867) using the same parameters outlined above.

### Liquid Fermentation

For liquid fermentation of 20AD (DSM 112866), 2 mL (0.5%) of the homogenized inoculum was transferred into 12 2-L Erlenmeyer culture flasks each containing 400 mL of YM 6.3 medium. Glucose content was monitored daily, and incubation was stopped 5 days after glucose depletion, applying the described procedure.^15^

### Isolation of Compounds **1**–**6**

The strain *Laburnicola nematophila* 20AD (DSM 112866) was cultivated on solid-state BRFT and liquid YM 6.3 media. From the cultivation, 2.2 g and 234 mg were obtained, respectively. The BRFT culture extract (2.2 g) was purified using a FlashPure ID silica 24 g cartridge on a Grace Reveleris X2 flash chromatography system. As a mobile phase, solvent A (DCM 100%), solvent B (DCM 58%, acetone 40%, MeOH 2%), and solvent C (acetone 37.5%, DCM 37.5%, MeOH 25%) were used; flow rate: 32 mL min^–1^; gradient: 5 min B at 0%, increasing to 100% B in 20 min, maintaining 100% B for 5 min, changing to solvent system B and C, starting from 0% C, raising to 100% C in 10 min, keeping isocratic conditions at 100% C for 10 min. This separation yielded a fraction of 95 mg with a retention time (*t*_R_) 36.2–41.4 min. This fraction was further separated using a Gilson PLC 2250 preparative HPLC system equipped with a Gemini C_18_ column (250 × 50 mm, 10 μm, Phenomenex, Aschaffenburg, Germany) and applying the following parameters: solvent A (H_2_O + 0.1% FA), solvent B (MeCN + 0.1% FA), flow rate 40 mL min^–1^, gradient: starting conditions at 20% B for 5 min, increasing to 80% B in 45 min, then up to 100% B in 5 min, and holding B at 100% for 10 min. This chromatographic separation resulted in the isolation of **1** (0.8 mg; *t*_R_ = 45.0 min), **3** (1.3 mg; *t*_R_ = 62.0 min), and **2** (3.8 mg; *t*_R_ = 63.0 min). To separate the mycelial extract of YM 6.3 liquid culture (234 mg), the preparative system Büchi Pure-C850 FlashPrep was utilized. Compounds were purified using a Gemini C_18_ column (250 × 50 mm, 10 μm, Phenomenex, Aschaffenburg, Germany) with H_2_O + 0.1% FA as solvent A, MeCN + 0.1% FA as solvent B, and a flow rate of 40 mL min^–1^. The applied gradient elution began at 10% B for 5 min, increasing to 50% B over 30 min, continuing at 80% B in 30 min, progressing to 100% B in 10 min, and holding for an additional 10 min at 100% B. Metabolites **4** (3.0 mg; *t*_R_ = 44 min) and **6** (3.8 mg; *t*_R_ = 24.5 min) were successfully purified.

BRFT cultivation of K01 (DSM112867) resulted in a methanol extract of 3.8 g, which was fractionated by a Grace Reveleris X2 flash chromatography system utilizing a FlashPure ID silica 40 g cartridge and a flow rate of 40 mL min^–1^, using parameters outlined above. The fractions from 3.5 to 15.6 min were combined and dried under reduced pressure, yielding a solid residue (396 mg), which was divided into three parts and further processed with a Gilson PLC 2250 system equipped with a Gemini C_18_ column (250 × 50 mm, 10 μm, Phenomenex, Aschaffenburg, Germany); the mobile phase and the applied gradient were as follows: solvent A (H_2_O + 0.1% FA), solvent B (MeCN + 0.1% FA), flow rate: 40 mL min^–1^, gradient: starting at 5% B for 5 min, progressing to 80% B in 50 min, reaching 100% B after additional 10 minutes, and keeping the 100% B for 10 min. The collected fractions from 42.4 to 42.9 min were pooled and dried in vacuum, affording a solid residue (25 mg). For the isolation of compound **5** (4.2 mg, *t*_R_ = 44.5–47.0 min), the latter residue (25 mg) was further processed with a Nucleodur C_18_HTec column (250 × 20 mm, 10 μm, Macherey-Nagel, Düren, Germany) connected to a Gilson PLC 2050 system. Separation was achieved by the following conditions: mobile phase: solvent A (H_2_O + 0.1% FA), solvent B (MeCN + 0.1% FA), flow rate: 20 mL min^–1^, gradient: 5 min at 5% B, increasing to 25% B in 10 min, holding at 25% B for 35 min, progressing to 100% B in 5 min, and maintaining 100% B for additional 10 minutes.

#### Dactylfungin C (**1**)

White amorphous solid; [α]_D_^20^ +4 (*c* 0.1, MeOH); UV–vis (MeOH) λ_max_ (log ε) = 283.0 (3.7), 236.5 (4.5), 217.0 (4.4) nm; ECD (MeOH, λ (nm) (Δε), *c* 3.69 × 10^–4^ M) 279 (+1.80), 241 (−8.66), 221sh (+0.95), 214 (+3.98), 202sh (−1.32), 197 (−3.17); NMR data (^1^H NMR: 700 MHz, ^13^C NMR: 175 MHz, methanol-*d*_4_) see Table 1; HR-(+)ESI-MS *m*/*z* 659.4156 [M – H_2_O + H]^+^ (calcd 659.4154 for C_38_H_59_O_9_^+^), *m*/*z* 677.4257 [M + H]^+^ (calcd 677.4259 for C_38_H_61_O_10_^+^), *m*/*z* 699.4076 [M + Na]^+^ (calcd 699.4079 for C_38_H_60_NaO_10_^+^), *t*_R_ = 10.67 min (LC-ESI-MS). C_38_H_60_O_10_ (676.34 g/mol).

#### Dactylfungin D (**2**)

Yellow amorphous solid; [α]_D_^20^ −4 (*c* 0.1, MeOH); UV–vis (MeOH) λ_max_ (log ε) = 283.0 (3.8), 236 (4.6), 219 (4.5) nm; ECD (MeOH, λ (nm) (Δε), *c* 3.78 × 10^–4^ M) 278 (+2.22), 244 (−10.76), 238sh (−10.15), 223sh (+2.20), 215 (+5.66), 201sh (−2.45); NMR data (^1^H NMR: 700 MHz, ^13^C NMR: 175 MHz, methanol-*d*_4_) see Table 1; HR-(+)ESI-MS *m*/*z* 643.4209 [M – H_2_O + H]^+^ (calcd 643.4204 for C_38_H_59_O_8_^+^), *m*/*z* 661.4316 [M + H]^+^ (calcd 661.4310 for C_38_H_61_O_9_^+^), *m*/*z* 683.4131 [M + Na]^+^ (calcd 683.4130 for C_38_H_60_NaO_9_^+^); *t*_R_ = 14.50 min (LC-ESI-MS). C_38_H_60_O_9_ (660.31 g/mol).

#### YM-202204 (**3**)

Yellow amorphous solid; [α]_D_^20^ −14 (*c* 0.1, MeOH); UV–vis (MeOH) λ_max_ (log ε) = 404.5 (3.9), 279.0 (4.3), 234.5 (4.7), 215.5 (4.7) nm; ECD (MeOH, λ (nm) (Δε), *c* 3.86 × 10^–4^ M) 297 (+0.33), 273 (+0.51), 245 (−5.31), 226 (+4.72), 214sh (+2.15), 199 (−1.20), 194 (+8.34); NMR data (^1^H NMR, 2D NMR: 700 MHz, methanol-*d*_4_) comparable to those reported in the literature;^17^ HR-(+)ESI-MS *m*/*z* 647.4152 [M + H]^+^ (calcd 647.4154 for C_37_H_59_O_9_^+^), *m*/*z* 669.3972 [M + Na]^+^ (calcd 669.3973 for C_37_H_58_NaO_9_^+^); *t*_R_ = 14.10 min (LC-ESI-MS). C_37_H_58_O_9_ (646.32 g/mol).

#### Laburnicolin (**4**)

Brown amorphous solid; [α]_D_^20^ −114 (*c* 0.1, MeOH); UV–vis (MeOH) λ_max_ (log ε) = 339 (3.7), 280 (4.1), 240.5 (4.2), 210 (4.2) nm; ECD (MeOH, λ (nm) (Δε), *c* 5.84 × 10^–4^ M) 283 (+0.64), 277sh (+0.63), 234 (+0.56), 214sh (−0.26), 204 (−1.01), 196 (+1.81); NMR data (^1^H: 500 MHz, ^13^C NMR: 125 MHz, DMSO-*d*_6_) see Table 2; HR-(+)ESI-MS *m*/*z* 411.1024 [M – H_2_O + H]^+^ (calcd 411.1074 for C_22_H_19_O_8_^+^), *m*/*z* 429.1175 [M + H]^+^ (calcd 429.1180 for C_22_H_21_O_9_^+^), *m*/*z* 451.0990 [M + Na]^+^ (calcd 451.1000 for C_22_H_20_NaO_9_^+^); *t*_R_ = 7.86 min (LC-ESI-MS). C_22_H_20_O_9_ (428.39 g/mol).

#### Laburnicolenone (**5**)

White amorphous solid; [α]_D_^20^ −9 (*c* 0.1, MeOH); UV–vis (MeOH) λ_max_ (log ε) = 287 (3.9), 206 (3.7) nm; ECD (MeOH, λ (nm) (Δε), *c* 9.05 × 10^–4^ M) 352 (−0.19), 324sh (−0.65), 314 (−1.30), 303sh (−0.26), 292sh (+1.35), 284 (+1.64), 275sh (+1.30), 241 (+0.79), 222 (−1.14), 201 (−1.56); NMR data (^1^H NMR: 500 MHz, ^13^C NMR: 125 MHz, methanol-*d*_4_) see Table 2; HR-(+)ESI-MS *m*/*z* 259.0967 [M – H_2_O + H]^+^ (calcd 259.0965 for C_15_H_15_O_4_^+^), *m*/*z* 277.1070 [M + H]^+^ (calcd 277.1071 for C_15_H_17_O_5_^+^), *m*/*z* 299.0890 [M + Na]^+^ (calcd 299.0890 for C_15_H_16_NaO_5_^+^); *t*_R_ = 6.29 min (LC-ESI-MS). C_15_H_16_O_5_ (276.29 g/mol).

#### 10-Norparvulenone (**6**)

Yellow amorphous solid; [α]_D_^20^ +19 (*c* 0.1, MeOH); UV–vis (MeOH) λ_max_ (log ε) = 284 (4.1), 224 (4.2) nm; ECD (MeOH, λ (nm) (Δε), *c* 1.05 × 10^–3^ M) 323sh (−0.26), 311sh (−0.64), 305 (−0.68), 280 (+1.55), 271sh (+1.16), 238 (0.55), 216 (−2.32), 208sh (−1.81), 199sh (+0.09), 192 (+1.97); NMR data (^1^H NMR: 500 MHz, ^13^C NMR: 125 MHz, DMSO-*d*_6_, methanol-*d*_4_) comparable to those reported in the literature;^22^ HR-(+)ESI-MS *m*/*z* 221.0803 [M – H_2_O + H]^+^ (calcd 221.0808 for C_12_H_13_O_4_^+^), *m*/*z* 261.0730 [M + Na]^+^ (calcd 261.0733 for C_12_H_14_NaO_5_^+^); *t*_R_ = 3.68 min (LC-ESI-MS). C_12_H_14_O_5_ (238.24 g/mol).

### Antimicrobial Assay

The antimicrobial activity was determined by a serial dilution assay to assess the minimum inhibitory concentration (MIC) of the isolated metabolites against different Gram-positive (*Bacillus subtilis*, *Mycolicibacterium smegmatis*, and *Staphylococcus aureus*) and Gram-negative (*Acinetobacter baumannii*, *Chromobacterium violaceum*, *Escherichia coli*, and *Pseudomonas aeruginosa*) bacteria and against five fungi (*Candida albicans*, *Mucor hiemalis*, *Rhodotorula glutinis*, *Schizosaccharomyces pombe*, and *Wickerhamomyces anomalus*), applying the same methods as previously described.^20^ A 20 μL amount of methanol was used as a negative control, and positive controls were selected based on the tested organism. Oxytetracycline, ciprobay, kanamycin, and gentamicin were used as controls for *B. subtilis*, *A. baumannii*, *M. smegmatis*, and *P. aeruginosa*, respectively, and nystatin was used for all fungal strains. In-depth analysis of the antifungal activity was performed as described by Štěpánek et al.^42^ in a serial dilution assay. Methanol was used as a negative and amphotericin B as a positive control. The fungal strains, which are important human pathogens, were incubated in malt extract broth (Oxoid, Basingstoke, UK) for filamentous fungi and in YM 6.3 for yeasts in combination with the metabolites (**1**–**3**) in a concentration range of 100–0.13, 33–0.13, and 16.7–0.013 μg mL^–1^. After 12 h (*C. albicans*), 48 h (*A. fumigatus* CCF 3522, *Cr. neoformans*, and *M. plumbeus*), and 72 h (others) the MIC was determined according to the published guidelines.^43^ All MIC values were determined in biological duplicates.

### Cytotoxicity Assay

Compounds **1**–**6** were tested for their cytotoxic effects on mouse fibroblast (L929) and human endocervical adenocarcinoma (KB3.1) cell lines using the MTT (3-(4,5-dimethylthiayol-2-yl)-2,5-diphenyltetrazolium bromide) test. The assay was performed with biological duplicates as previously reported.^20^ Epothilone B was used as a positive control.

### Nematicidal Assay

Nematicidal effects were assessed with *Caenorhabditis elegans* in a 48-well flat-bottom plate. Metabolites **1**, **2**, and **4**–**6** were tested at concentrations of 100, 50, and 10 μg mL^–1^ in biological triplicates. Ivermectin was used as a positive control at a concentration of 1 μg mL^–1^, and methanol as a negative control. The assay was performed as described by Phutthacharoen et al.^44^

### Computational Section

Mixed torsional/low-mode conformational searches were carried out by means of the Macromodel 10.8.011 software using the MMFF with an implicit solvent model for CHCl_3_ and applying a 21 kJ mol^–1^ energy window.^45^ Geometry reoptimizations of the resultant conformers (ωB97X/TZVP PCM/MeOH) and TDDFT-ECD (B3LYP/TZVP PCM/MeOH, BH&HLYP/TZVP PCM/MeOH, CAM-B3LYP/TZVP PCM/MeOH, and PBE0/TZVP PCM/MeOH) calculations were performed with the Gaussian 16 package.^46^ ECD spectra were generated as sums of Gaussians with a 4200 cm^–1^ width at half-height, using dipole-velocity-computed rotational strength values.^47^ Boltzmann distributions were estimated from the DFT energies. Visualization of the results was performed by the MOLEKEL 5.4 software package.^48^

## Data Availability

All data related to structure elucidation and bioassays are available as Supporting Information.

## Supporting Information Available

LR-ESI-MS, HR-ESI-MS, 1D (^1^H/^13^C) and 2D (^1^H–^1^H COSY, HMBC, HSQC and ROESY) NMR, and ECD spectra of compounds **1**–**6** (PDF)
