# Supplementary material for: Dactylfungins and Tetralones: Bioactive Metabolites from a Nematode-Associated Laburnicola nematophila
Source: J Nat Prod. 2024 Jul 16;87(7):1860–71. doi: 10.1021/acs.jnatprod.4c00623 (PMC11287750; doi:10.1021/acs.jnatprod.4c00623)
Supplement: Supplementary file 1 — np4c00623_si_001.pdf [file np4c00623_si_001.pdf]

Supporting Information for:

Dactylfungins and Tetralones: Bioactive Metabolites from a Nematode-Associated *Laburnicola nematophila*

Jan-Peer Wennrich,[†,‡] Caren Holzenkamp,[†,‡] Miroslav Kolařík,[§] Wolfgang Maier,[⊥] Attila Mándi,[¶] Tibor Kurtán,[¶] Samad Ashrafi,[⊥,∇] Sherif S. Ebada,[†,Δ]* and Marc Stadler[†,‡]*

[†] Department of Microbial Drugs, Helmholtz Centre for Infection Research (HZI) and German Centre for Infection Research, Inhoffenstrasse 7, 38124 Braunschweig, Germany.

[‡] Institute of Microbiology, Technische Universität Braunschweig, Spielmannstraße 7, 38106 Braunschweig, Germany.

[§] Institute of Microbiology, Czech Academy of Science, Vídeňská 1083, 14220 Prague, Czech Republic

[⊥] Institute for Epidemiology and Pathogen Diagonstics, Julius Kühn Institut (JKI) - Federal Research Centre for Cultivated Plants, Messeweg 11-12, 38104 Braunschweig, Germany.

[¶] Department of Organic Chemistry, University of Debrecen, P. O. Box 400, 4002 Debrecen, Hungary.

[∇] Institute for Crop and Soil Science, Julius Kühn Institute (JKI) – Federal Research Centre for Cultivated Plants, Bundesallee 58, 38116 Braunschweig, Germany.

[Δ] Department of Pharmacognosy, Faculty of Pharmacy, Ain Shams University, Cairo 11566, Egypt

* Correspondence:

E-mail: sherif.elsayed@helmholtz-hzi.de, sherif_elsayed@pharma.asu.edu.eg (S.S.E); Marc.Stadler@helmholtz-hzi.de (M.S.); Tel.: +49-531-6181-424, Fax: +49-531-6181-9499

Contents of Supporting Information

#	Contents	Page
**1**	Figure S1. LR-ESI-MS spectra of **1**.	**S4**
**2**	Figure S2. HR-ESI-MS spectra of **1**.	**S5**
**3**	Figure S3. ^{1}H NMR spectrum of **1** in methanol-d_4 at 700 MHz.	**S6**
**4**	Figure S4. ^{13}C NMR spectrum of **1** in methanol-d_4 at 175 MHz.	**S7**
**5**	Figure S5. ^{1}H-^{1}H COSY spectrum of **1** in methanol-d_4 at 700 MHz.	**S8**
**6**	Figure S6. HMBC spectrum of **1** in methanol-d_4 at 700 MHz.	**S9**
**7**	Figure S7. HSQC spectrum of **1** in methanol-d_4 at 700 MHz.	**S10**
**8**	Figure S8. ROESY spectrum of **1** in methanol-d_4 at 700 MHz.	**S11**
**9**	Figure S9. LR-ESI-MS spectra of **2**.	**S12**
**10**	Figure S10. HR-ESI-MS spectra of **2**.	**S13**
**11**	Figure S11. ^{1}H NMR spectrum of **2** in methanol-d_4 at 700 MHz.	**S14**
**12**	Figure S12. ^{13}C NMR spectrum of **2** in methanol-d_4 at 175 MHz.	**S15**
**13**	Figure S13. ^{1}H-^{1}H COSY spectrum of **2** in methanol-d_4 at 700 MHz.	**S16**
**14**	Figure S14. HMBC spectrum of **2** in methanol-d_4 at 700 MHz.	**S17**
**15**	Figure S15. HSQC spectrum of **2** in methanol-d_4 at 700 MHz.	**S18**
**16**	Figure S16. ROESY spectrum of **2** in methanol-d_4 at 700 MHz.	**S19**
**17**	Figure S17. LR-ESI-MS spectra of **3**.	**S20**
**18**	Figure S18. HR-ESI-MS spectra of **3**.	**S21**
**19**	Figure S19. ^{1}H NMR spectrum of **3** in methanol-d_4 at 700 MHz.	**S22**
**20**	Figure S20. ^{1}H-^{1}H COSY spectrum of **3** in methanol-d_4 at 700 MHz.	**S23**
**21**	Figure S21. HMBC spectrum of **3** in methanol-d_4 at 700 MHz.	**S24**
**22**	Figure S22. HSQC spectrum of **3** in methanol-d_4 at 700 MHz.	**S25**
**23**	Figure S23. LR-ESI-MS spectra of **4**.	**S26**
**24**	Figure S24. HR-ESI-MS spectra of **4**.	**S27**
**25**	Figure S25. ^{1}H NMR spectrum of **4** in DMSO-d_6 at 500 MHz.	**S28**
**26**	Figure S26. ^{13}C NMR spectrum of **4** in DMSO-d_6 at 125 MHz.	**S29**
**27**	Figure S27. ^{1}H-^{1}H COSY spectrum of **4** in DMSO-d_6 at 500 MHz.	**S30**
**28**	Figure S28. HMBC spectrum of **4** in DMSO-d_6 at 500 MHz.	**S31**
**29**	Figure S29. HSQC spectrum of **4** in DMSO-d_6 at 500 MHz.	**S32**
**30**	Figure S30. ROESY spectrum of **4** in DMSO-d_6 at 700 MHz.	**S33**
**31**	Figure S31. LR-ESI-MS spectra of **5**.	**S34**
**32**	Figure S32. HR-ESI-MS spectra of **5**.	**S35**
**33**	Figure S33. ^{1}H NMR spectrum of **5** in methanol-d_4 at 500 MHz.	**S36**
**34**	Figure S34. ^{13}C NMR spectrum of **5** in methanol-d_4 at 125 MHz.	**S37**
**35**	Figure S35. ^{1}H-^{1}H COSY spectrum of **5** in methanol-d_4 at 500 MHz.	**S38**
**36**	Figure S36. HMBC spectrum of **5** in methanol-d_4 at 500 MHz.	**S39**
**37**	Figure S37. HSQC spectrum of **5** in methanol-d_4 at 500 MHz.	**S40**
**38**	Figure S38. ROESY spectrum of **5** in methanol-d_4 at 500 MHz.	**S41**
**39**	Figure S39. Kohn-Sham orbitals of (*R*)-**5** computed at the BH&HLYP/TZVP PCM/MeOH level.	**S42**
**40**	Figure S40. LR-ESI-MS spectra of **6**.	**S43**
**41**	Figure S41. HR-ESI-MS spectra of **6**.	**S44**
**42**	Figure S42. ^{1}H NMR spectrum of **6** in DMSO-d_6 at 500 MHz.	**S45**
**43**	Figure S43. ^{13}C NMR spectrum of **6** in DMSO-d_6 at 125 MHz.	**S46**
**44**	Figure S44. ^{1}H-^{1}H COSY spectrum of **6** in DMSO-d_6 at 500 MHz.	**S47**
**45**	Figure S45. HMBC spectrum of **6** in DMSO-d_6 at 500 MHz.	**S48**
**46**	Figure S46. HSQC spectrum of **6** in DMSO-d_6 at 500 MHz	**S49**
**47**	Figure S47. ROESY spectrum of **6** in DMSO-d_6 at 500 MHz.	**S50**
**48**	Figure S48. ^{1}H NMR spectrum of **6** in methanol-d_6 at 500 MHz.	**S51**
**49**	Figure S49. ^{13}C NMR spectrum of **6** in methanol-d_6 at 125 MHz.	**S52**
**50**	Figure S50. ^{1}H-^{1}H COSY spectrum of **6** in methanol-d_6 at 500 MHz.	**S53**
**51**	Figure S51. ROESY spectrum of **6** in methanol-d_6 at 500 MHz.	**S54**
**52**	Figure S52. Kohn-Sham orbitals of (*R*)-**6** computed at the CAM-B3LYP/TZVP PCM/MeOH.	**S55**

**53**	Figure S53. Experimental ECD spectrum of **5** in MeOH compared with the BH&HLYP/TZVP PCM/MeOH ECD spectra of the lowest-energy ωB97X/TZVP PCM/MeOH representatives of the two conformer groups of (*R*)-**5**; red: group A with a total population of 73.7% represented by conformer A (*M* helicity), blue: group B with a total population of 26.3% represented by conformer I (*P* helicity).	**S56**
**54**	Figure S54. Experimental ECD spectrum of **6** in MeOH compared with the CAM-B3LYP/TZVP PCM/MeOH ECD spectra of the lowest-energy ωB97X/TZVP PCM/MeOH representatives of the two conformer groups of (*R*)-**6**; red: group A with a total population of 65.9% represented by conformer A (*M* helicity), blue: group B with a total population of 30.5% represented by conformer I (*P* helicity).	**S56**
**55**	Table S1. Cytotoxicity (IC_{50}) and antimicrobial activity (MIC) of **1**–**6**.	**S57**
**56**	Table S2. Nematicidal activity of **1**, **2**, **4**–**6**.	**S57**

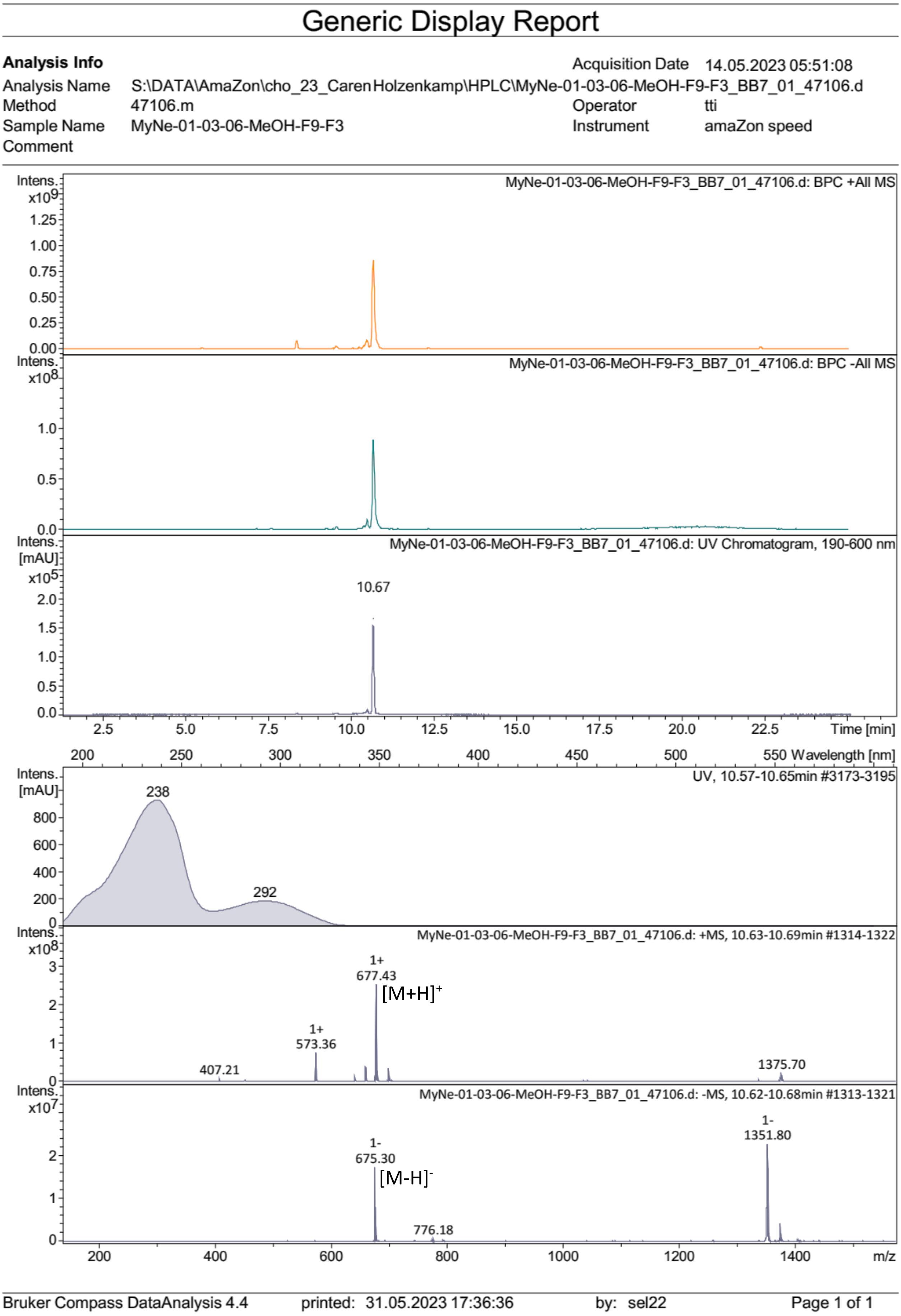

Figure S1. LR-ESI-MS of **1**.

Generic Display Report

Analysis Info

Analysis Name	S:\DATA\MaXis\cho23_CarenHolzenkamp\23_05\MyNe_01_03_06_MeOH_F9-F3_29_01_11748.d	Acquisition Date	31.05.2023 00:30:17
Method	pos_säure_10000_screening_ms_100_2500_line.m	Operator	ate06
Sample Name	MyNe_01_03_06_MeOH_F9-F3	Instrument	maXis
Comment	Screening01<br>Waters Acquity UPLC BEH C_18 1,7um 2.1x50mm		

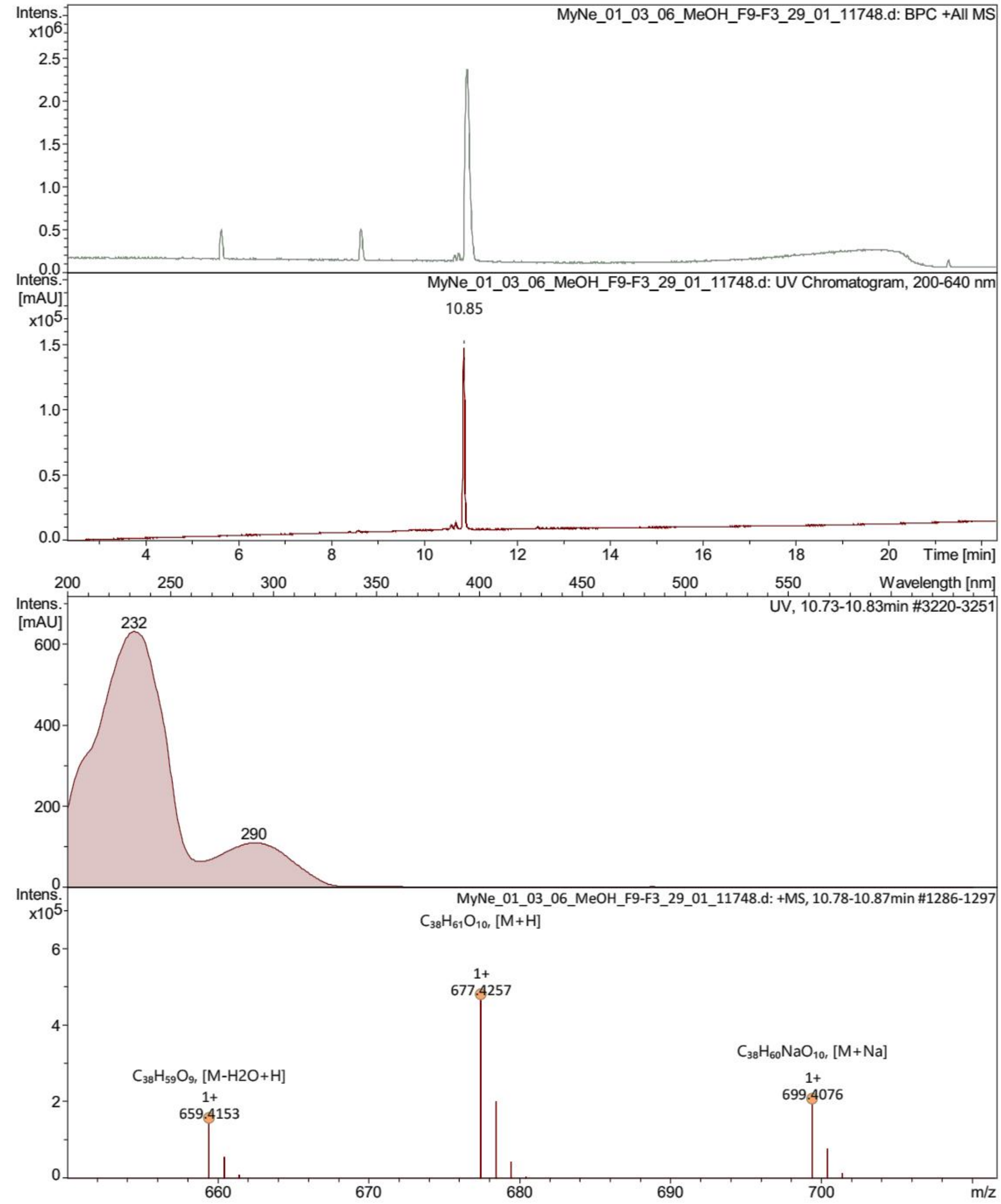

Figure S2. HR-ESI-MS of **1**.

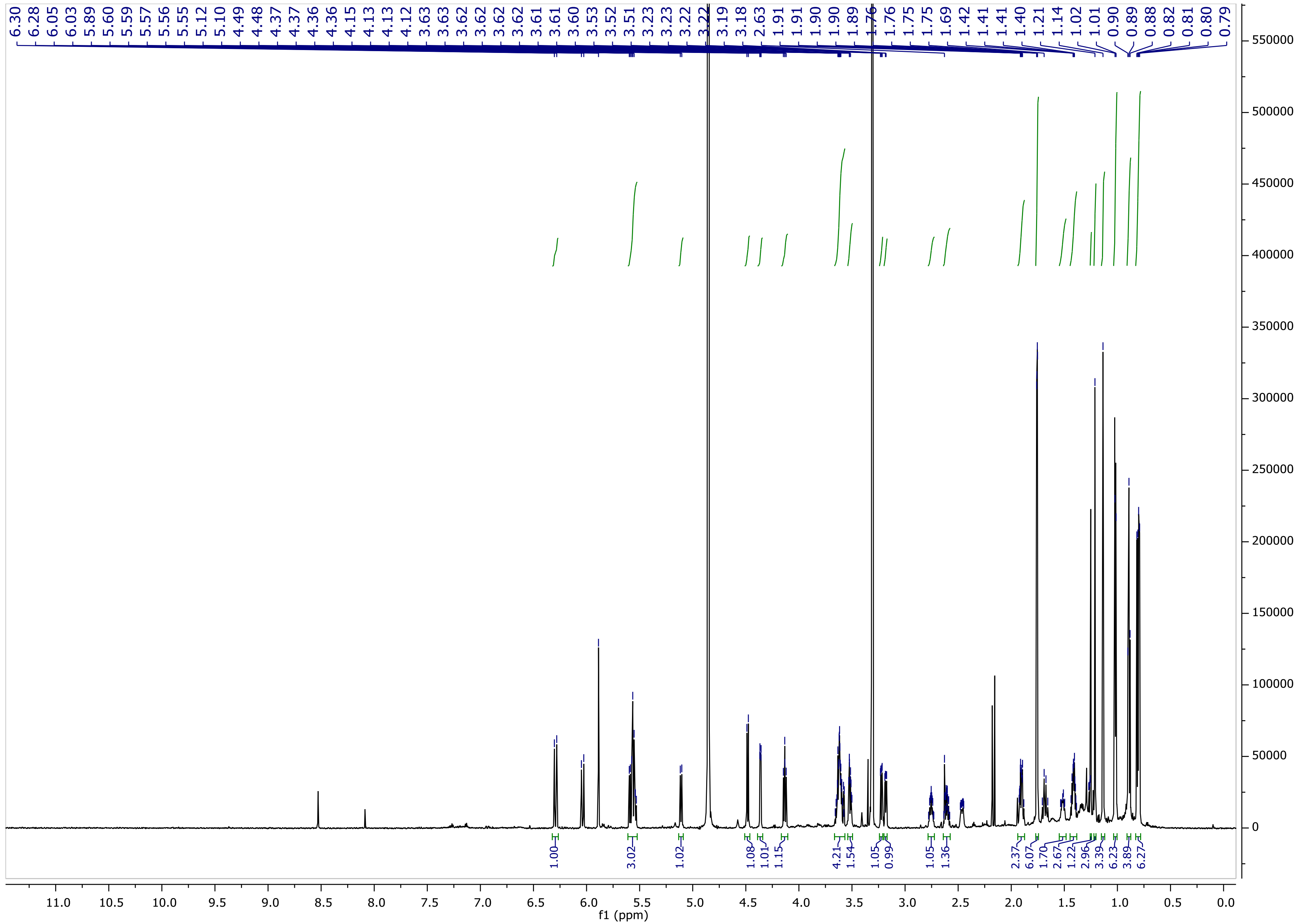

Figure S3. ^{1}H NMR spectrum of **1** in methanol-d_4 at 700 MHz.

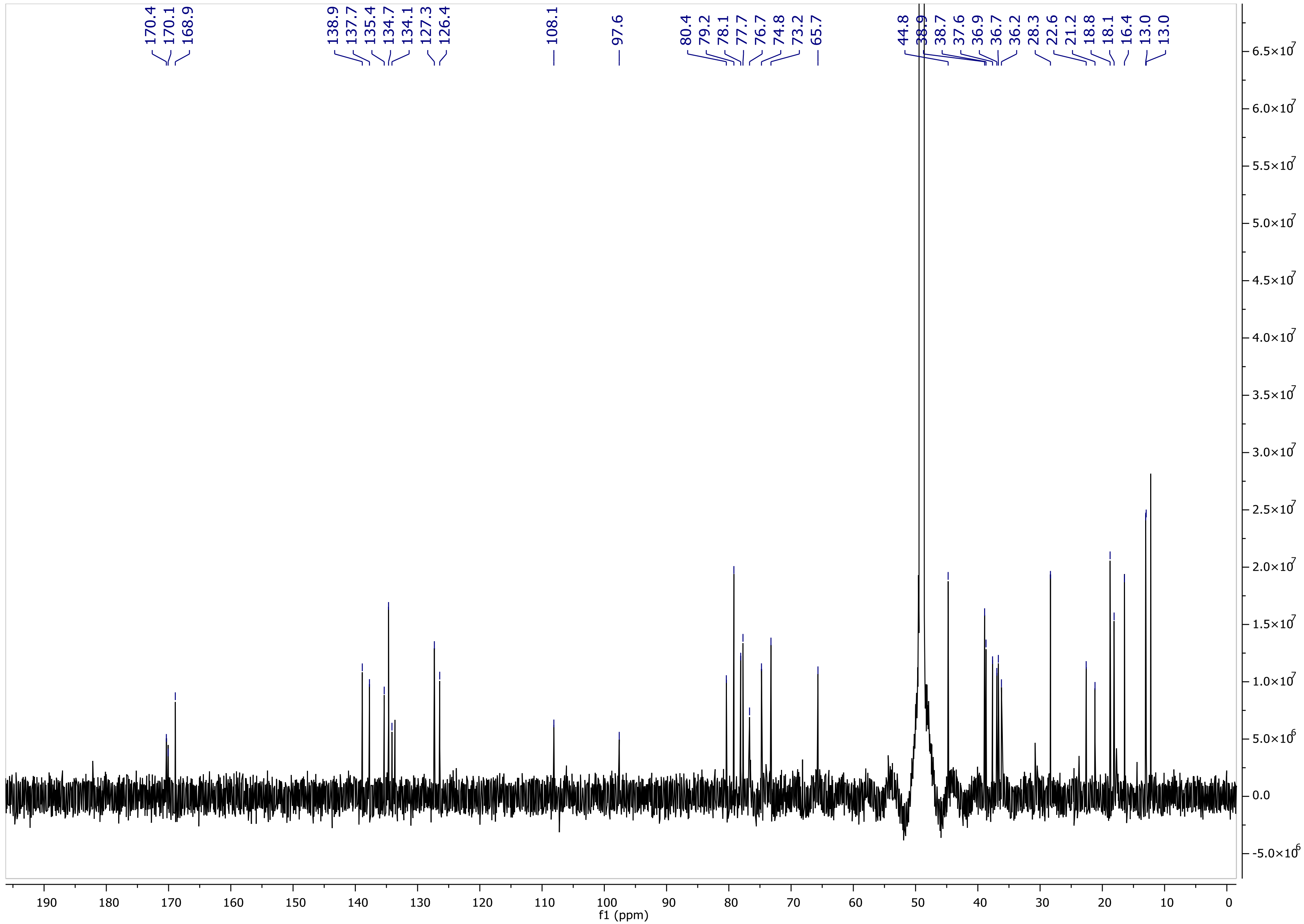

Figure S4. ^{13}C NMR spectrum of **1** in methanol-d_4 at 175 MHz.

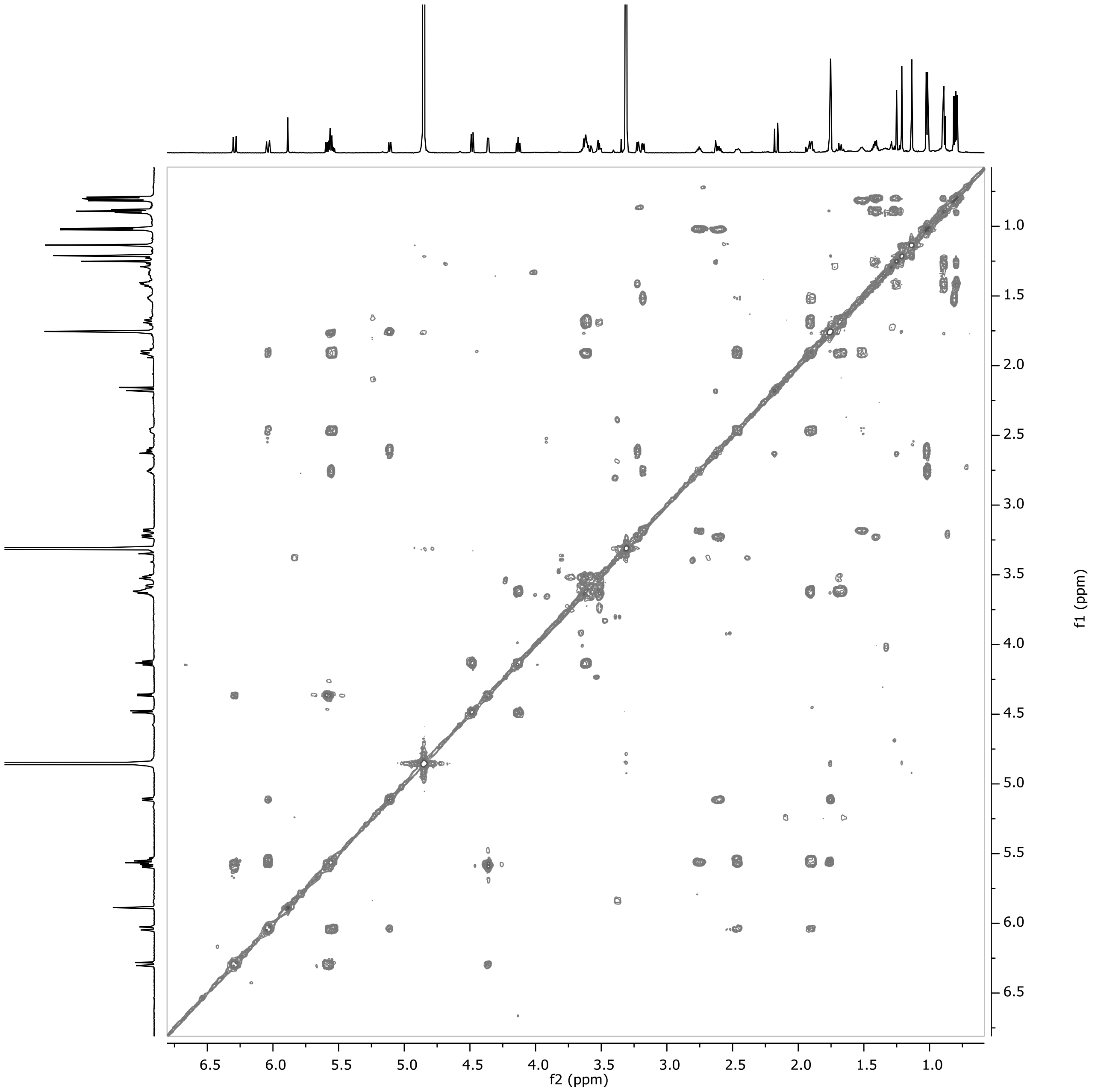

Figure S5. ^{1}H-^{1}H COSY spectrum of **1** in methanol-*d₄* at 700 MHz.

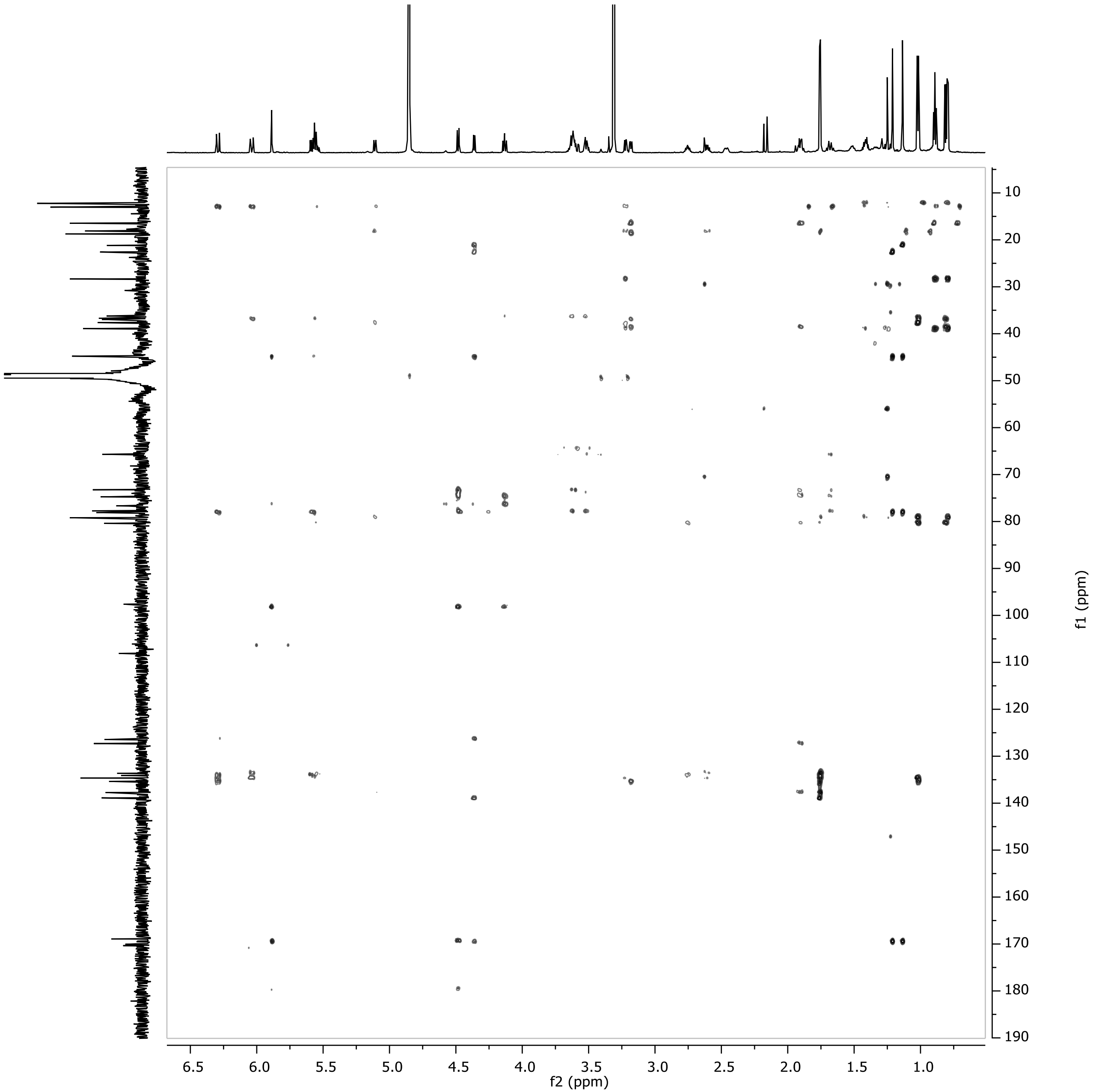

Figure S6. HMBC spectrum of **1** in methanol-d_4 at 700 MHz.

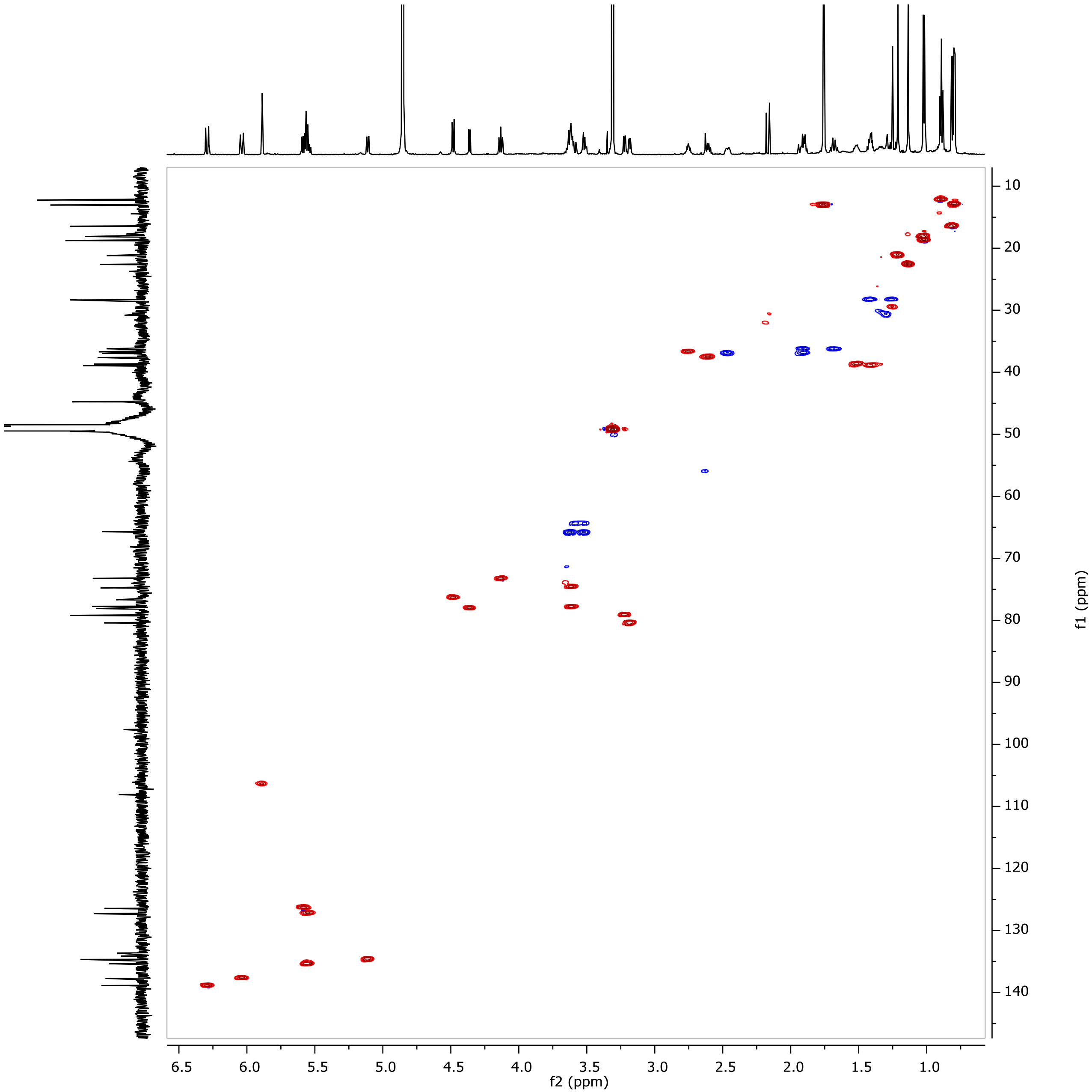

Figure S7. HSQC spectrum of **1** in methanol-d_4 at 700 MHz.

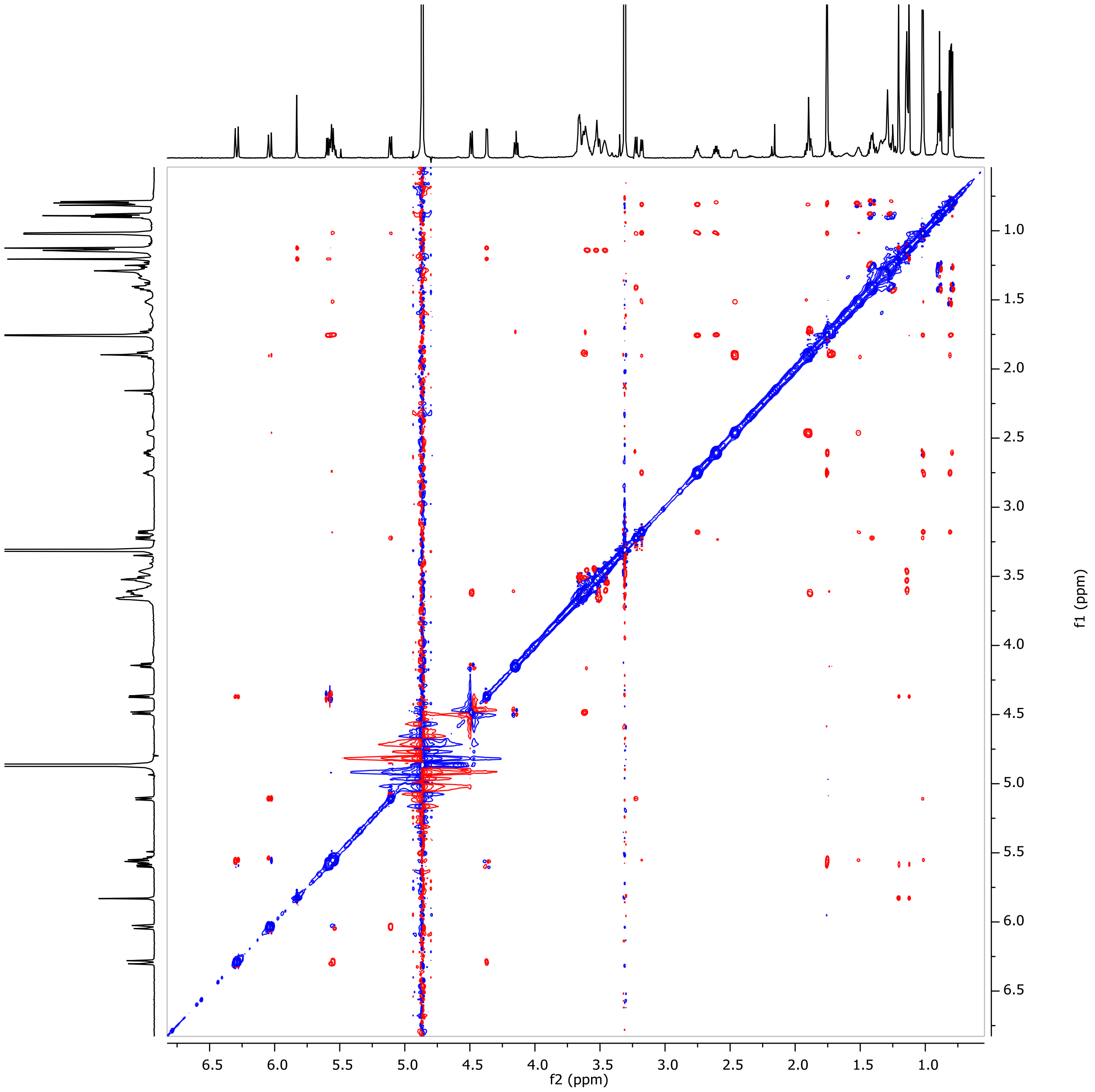

Figure S8. ROESY spectrum of **1** in methanol-d_4 at 500 MHz.

Generic Display Report

Analysis Info

Analysis Name	S:\DATA\AmaZon\cho_23_CarenHolzenkamp\HPLC\MyNe-01-03-06-MeOH-F8-F6_BA6_01_47094.d	Acquisition Date	13.05.2023 22:36:41
Method	47094.m	Operator	tti
Sample Name	MyNe-01-03-06-MeOH-F8-F6	Instrument	amaZon speed
Comment			

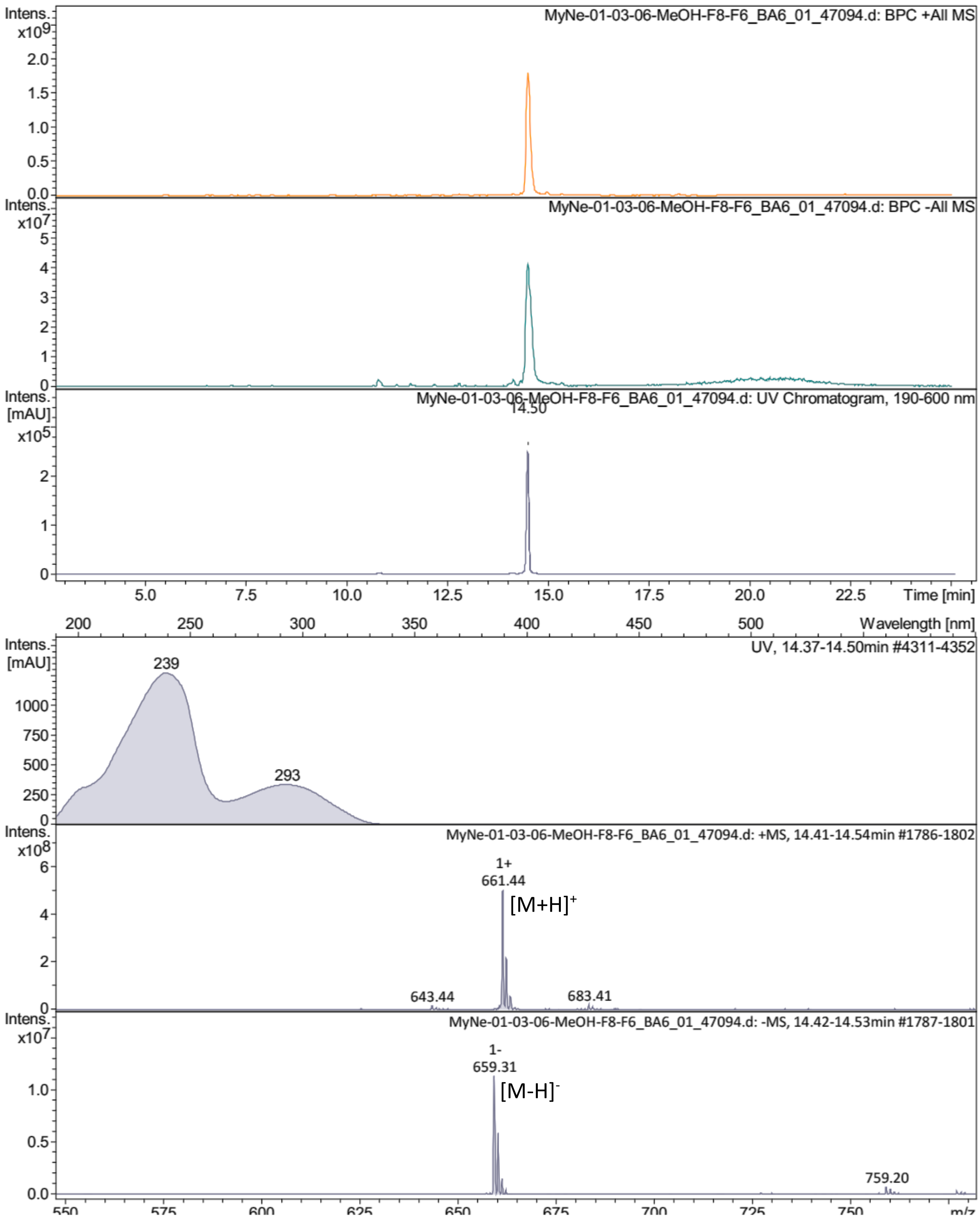

Bruker Compass DataAnalysis 4.4 printed: 31.05.2023 19:21:45 by: sel22 Page 1 of 1

Figure S9. LR-ESI-MS of **2**.

Generic Display Report

Analysis Info

Analysis Name	S:\DATA\MaXis\cho23_CarenHolzenkamp\23_05\MyNe_01_03_06_MeOH_F8-F6_28_01_11747.d	Acquisition Date	30.05.2023 23:59:18
Method	pos_säure_10000_screening_ms_100_2500_line.m	Operator	ate06
Sample Name	MyNe_01_03_06_MeOH_F8-F6	Instrument	maXis
Comment	Screening01<br>Waters Acquity UPLC BEH C_18 1,7um 2.1x50mm		

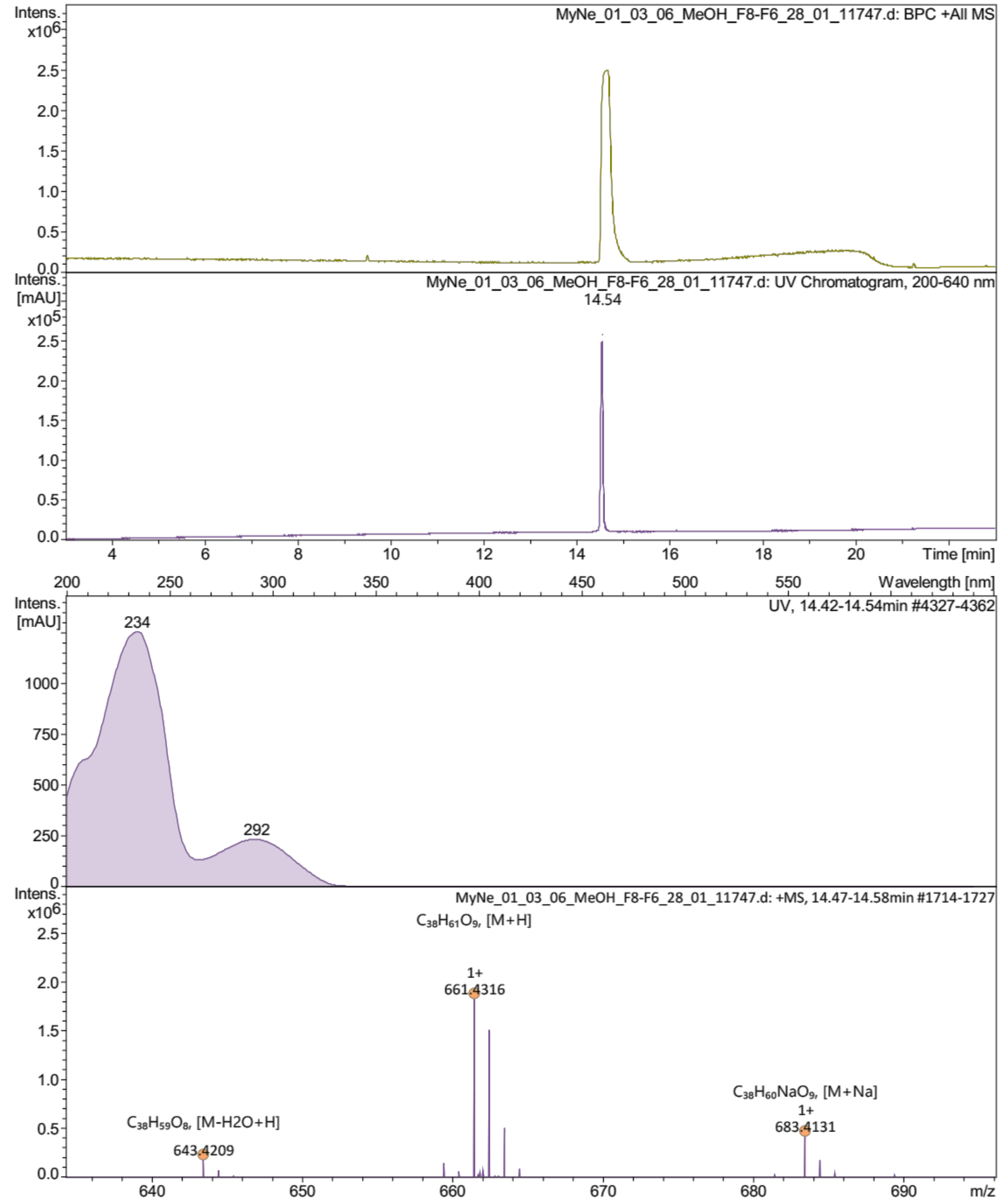

Bruker Compass DataAnalysis 4.4 printed: 24.07.2023 17:41:12 by: sel22 Page 1 of 1

Figure S10. HR-ESI-MS of **2**.

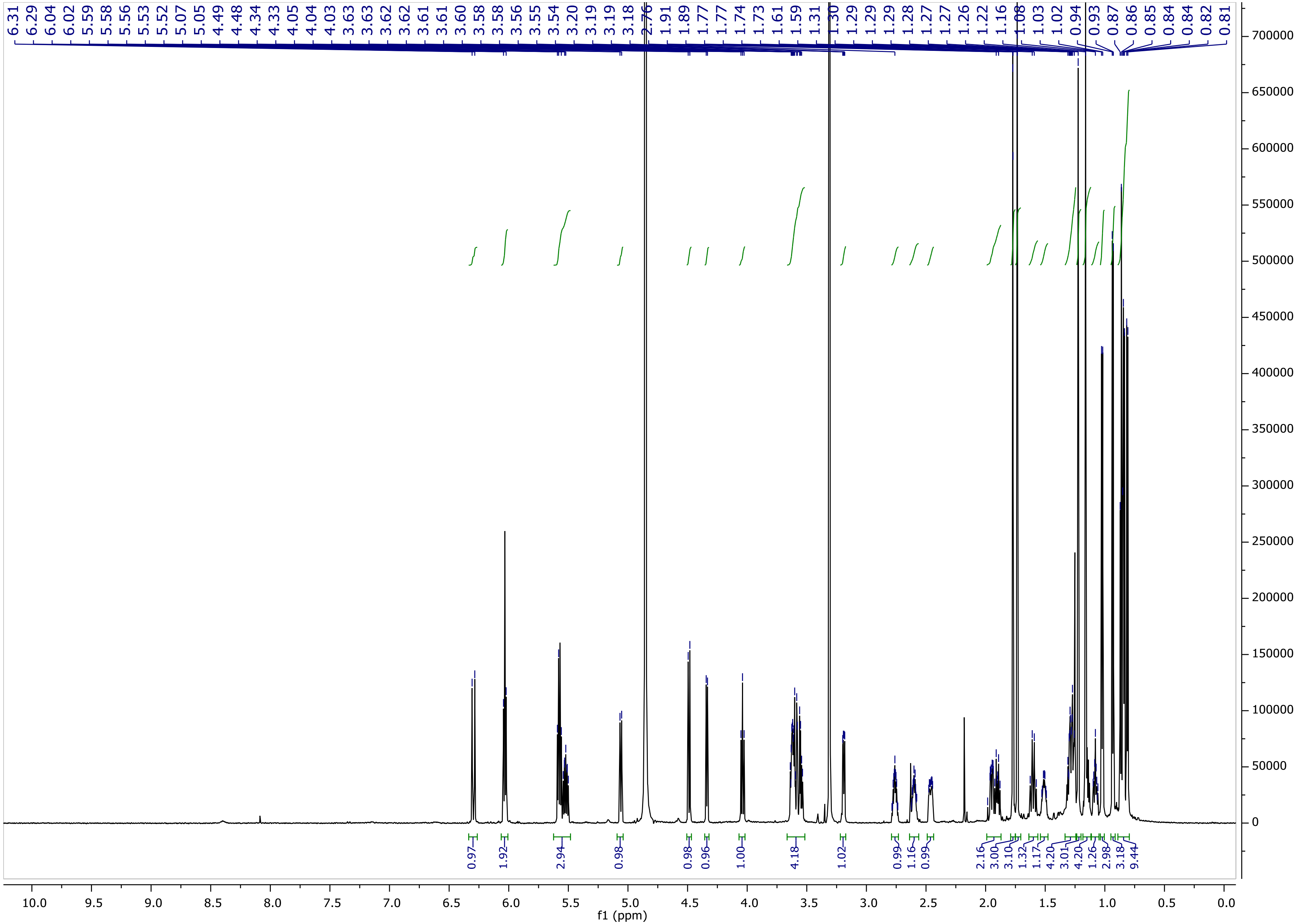

Figure S11. ^{1}H NMR spectrum of **2** in methanol-d_4 at 700 MHz.

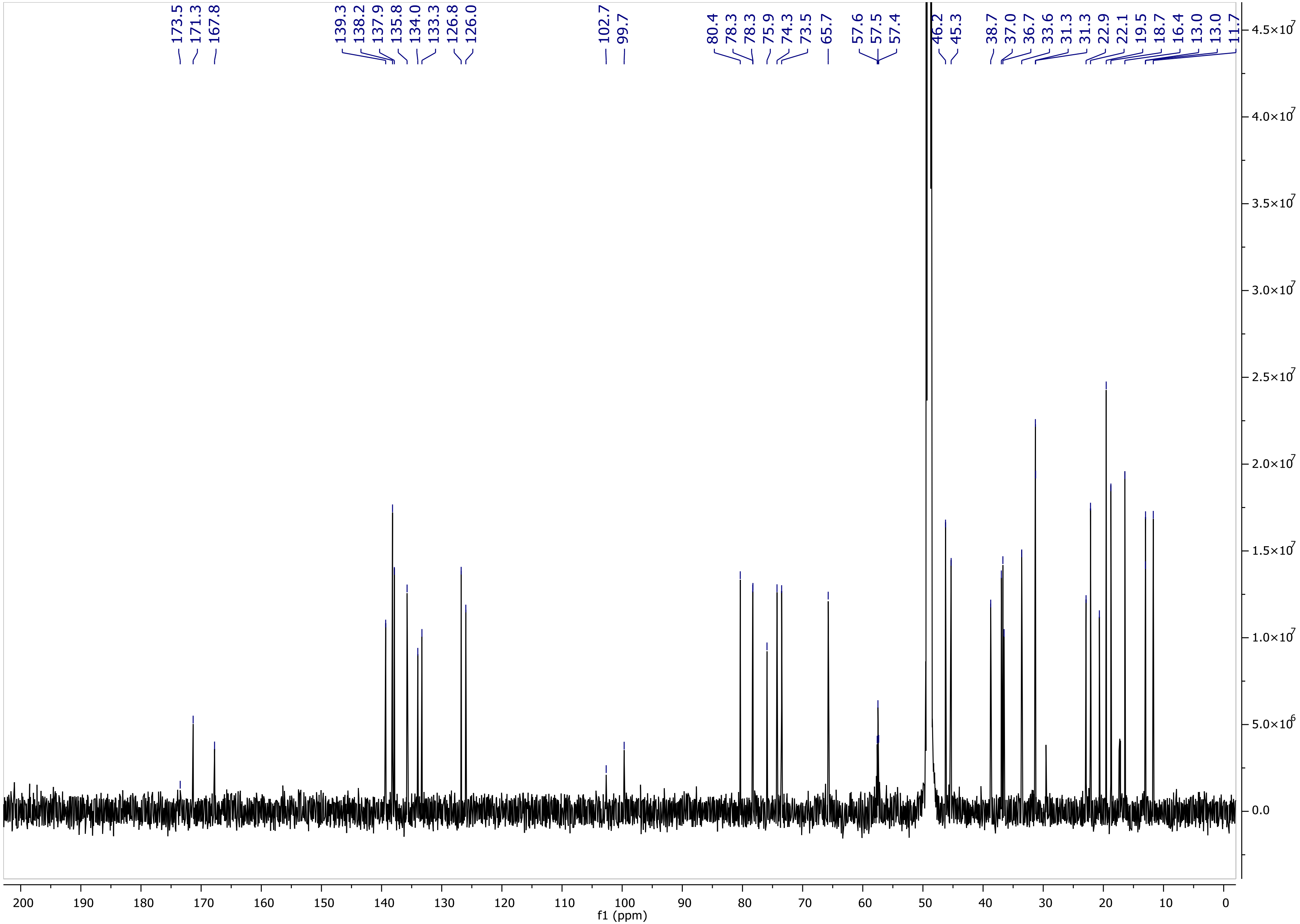

Figure S12. ^{13}C NMR spectrum of **2** in methanol-d_4 at 175 MHz.

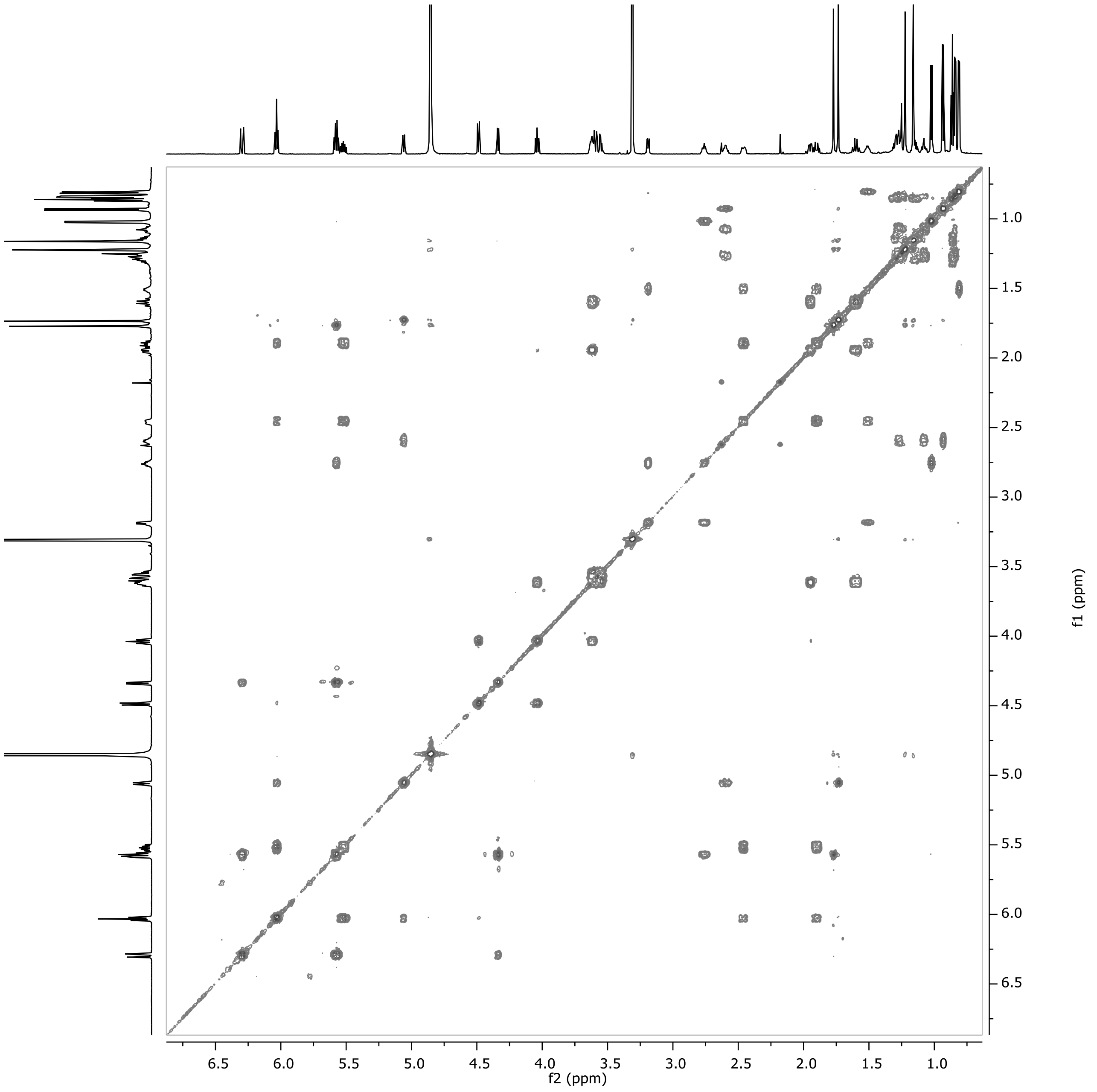

Figure S13. ^{1}H-^{1}H COSY spectrum of **2** in methanol-d_4 at 700 MHz.

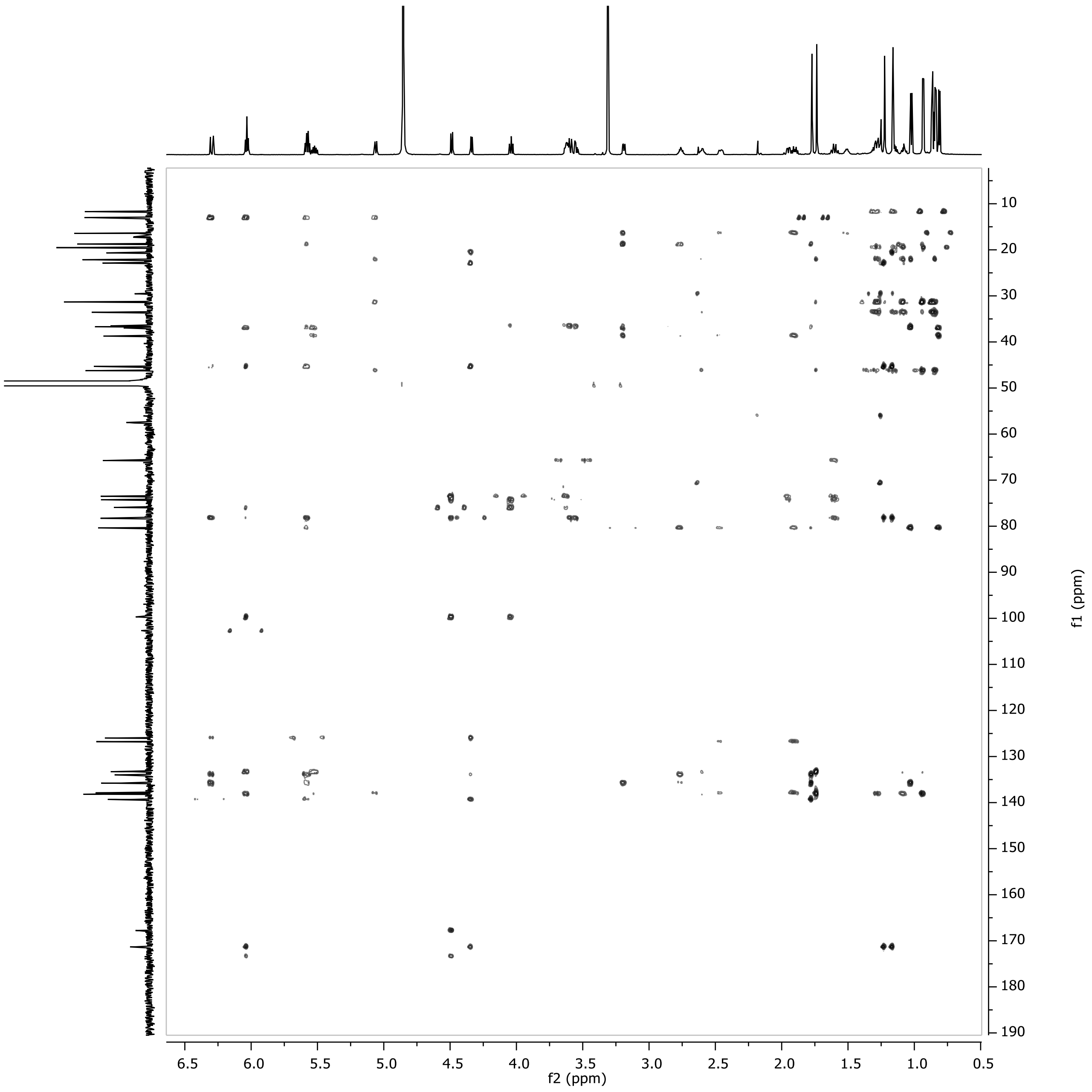

Figure S14. HMBC spectrum of **2** in methanol-d_4 at 700 MHz.

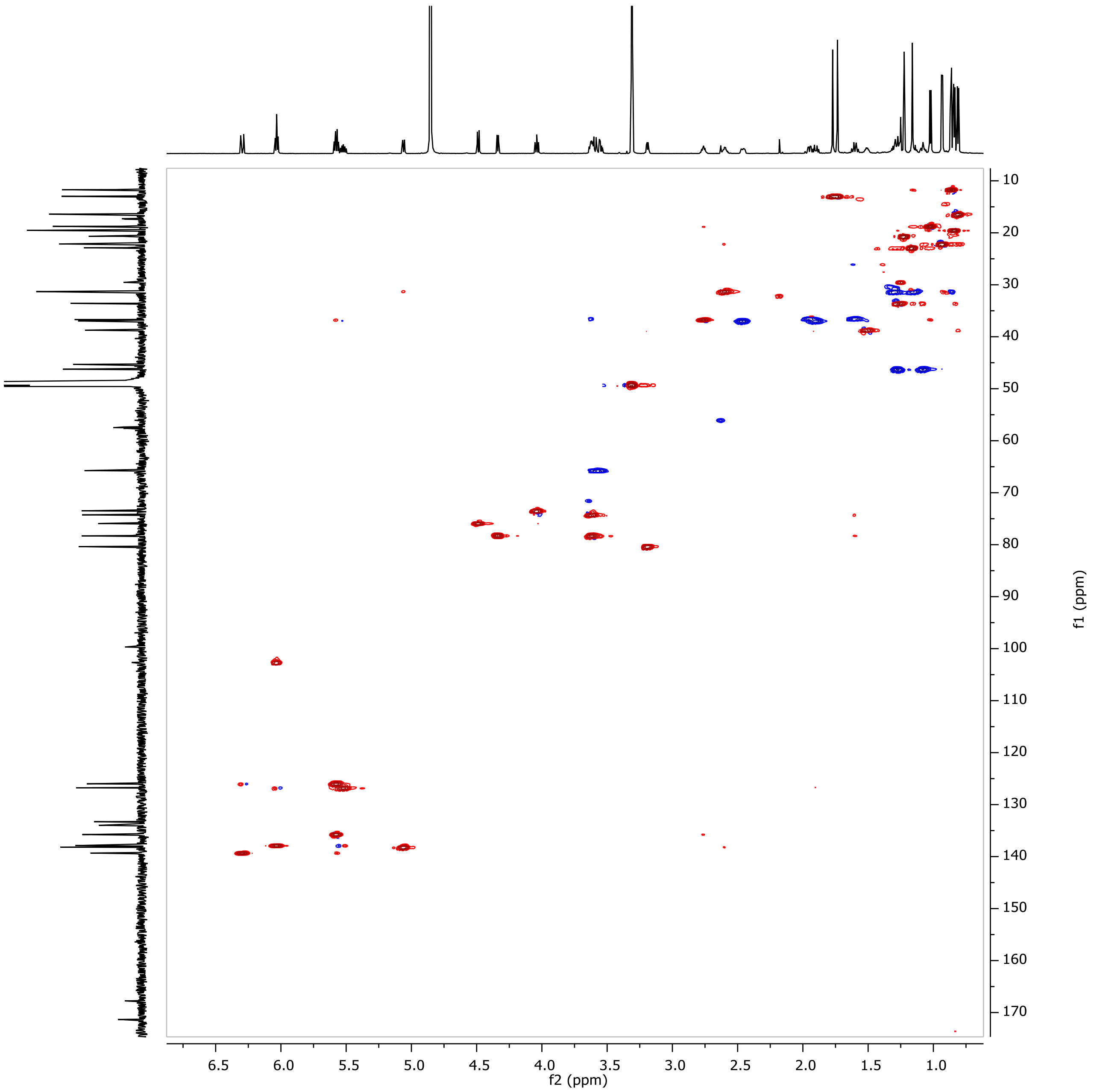

Figure S15. HSQC spectrum of **2** in methanol-d_4 at 700 MHz.

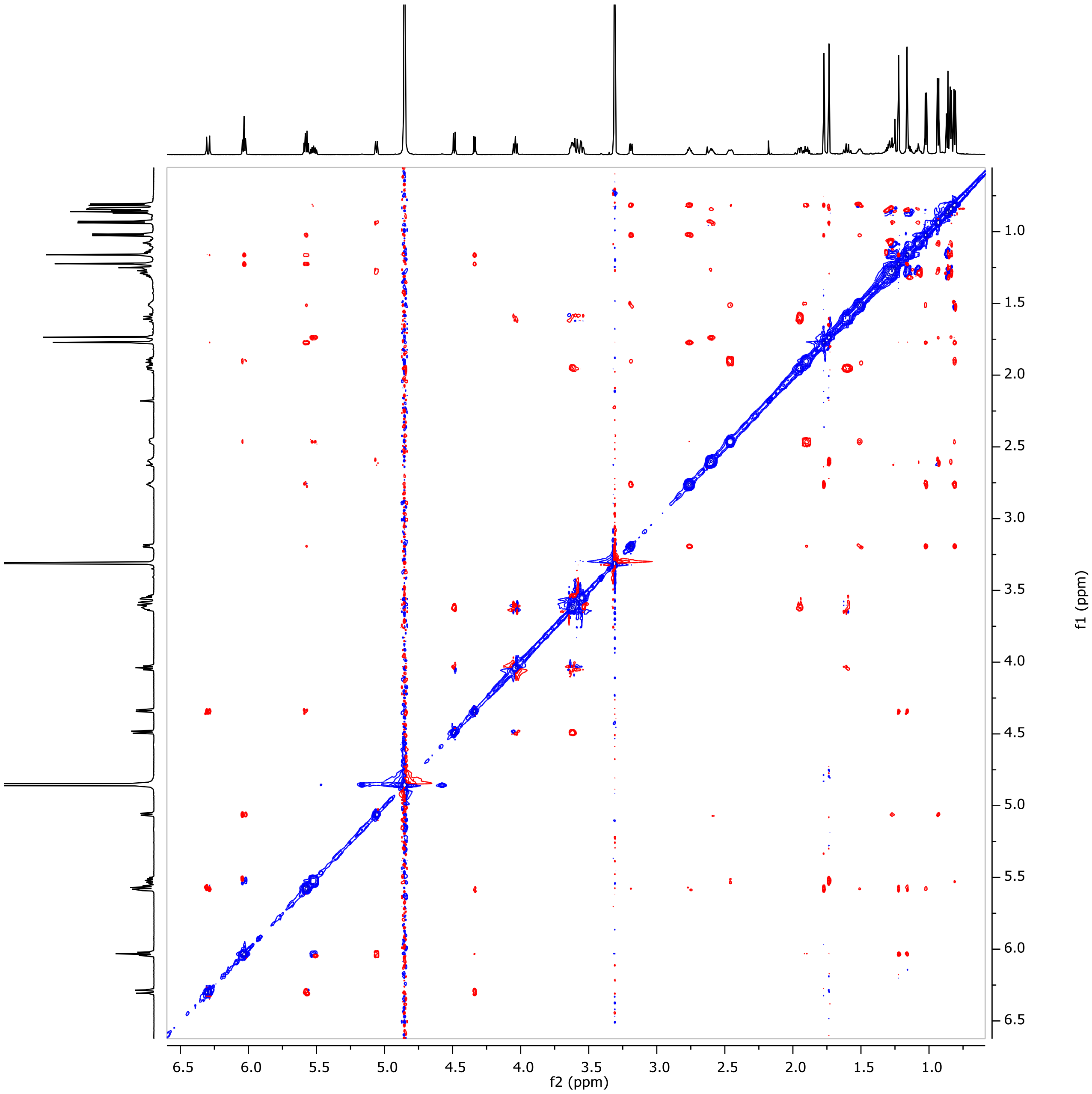

Figure S16. ROESY spectrum of **2** in methanol-d_4 at 700 MHz.

Generic Display Report

**Analysis Info**		Acquisition Date	17.08.2023 13:10:13
Analysis Name	S:\PEOPLE\cho23_Caren Holzenkamp\NMR\Maxis Data\purified fractions\MeOH-F11-F15-647		
Method	49830\MyNe-03-03-06-MeOH-F11-F15_GD2_01_49830.d	Operator	tti
Sample Name	MyNe-03-03-06-MeOH-F15	Instrument	amaZon speed
Comment			

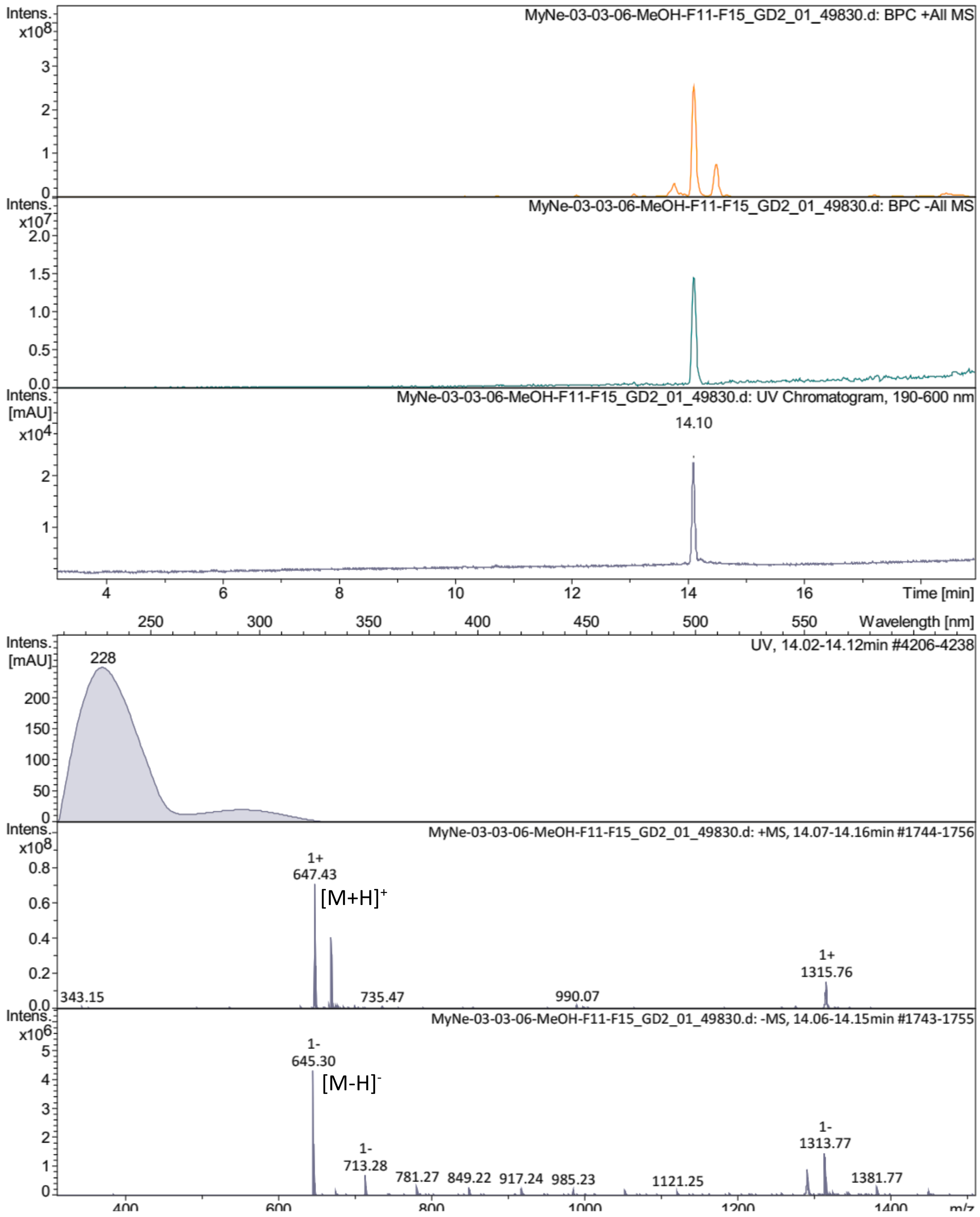

Bruker Compass DataAnalysis 4.4 printed: 12.10.2023 18:35:55 by: sel22 Page 1 of 1

Figure S17. LR-ESI-MS of **3**.

Generic Display Report

Analysis Info

Analysis Name	S:\PEOPLE\cho23_Caren Holzenkamp\NMR\Maxis Data\purified fractions\MeOH-F11-F15-647	Acquisition Date	27.09.2023 08:03:23
Method	[illegible].m	Operator	ate06
Sample Name	03-03-06-MeOH-F11-F15	Instrument	maXis
Comment	Screening01<br>Waters Acquity UPLC BEH C_18 1,7um 2.1x50mm		

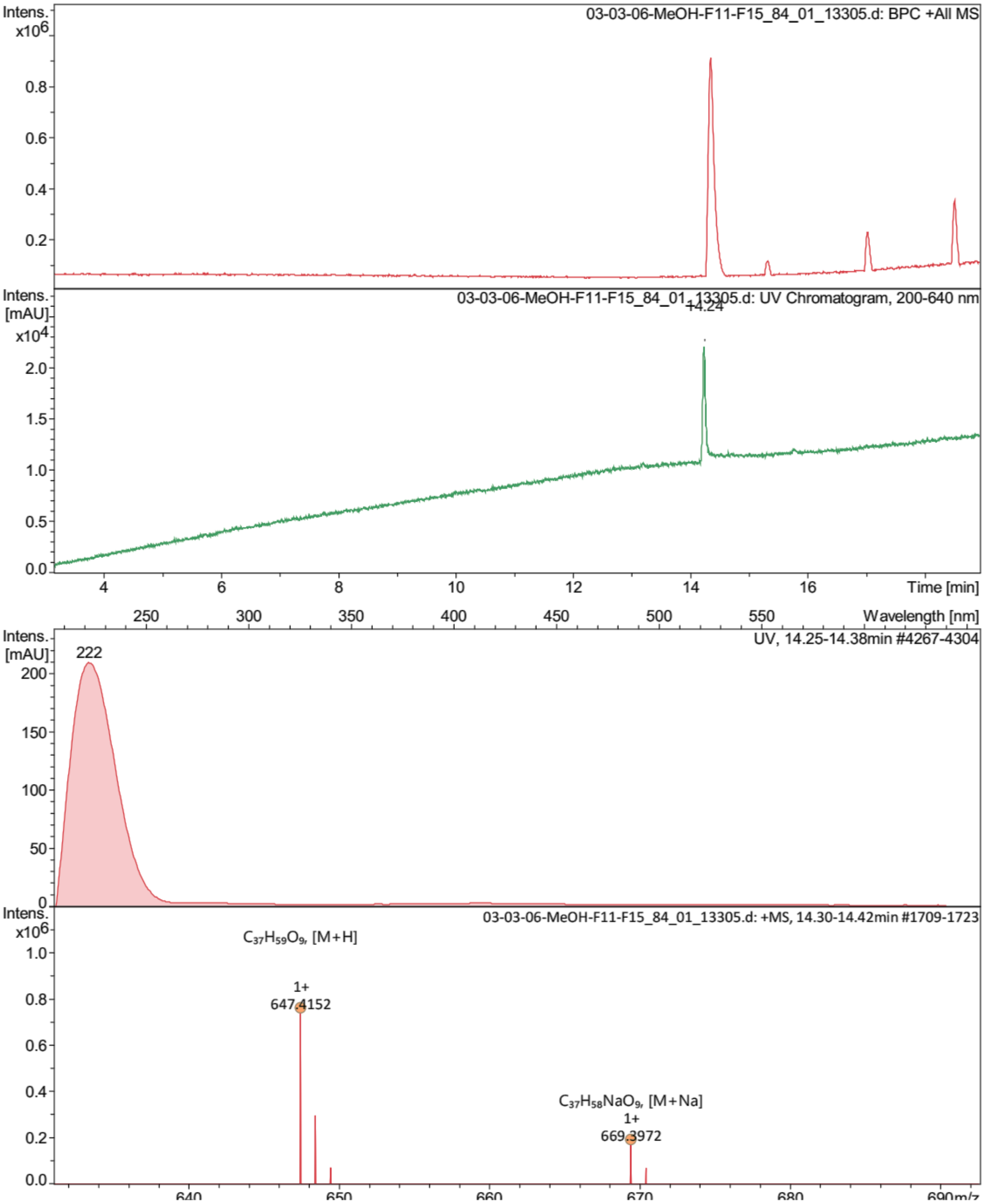

Bruker Compass DataAnalysis 4.4 printed: 12.10.2023 18:33:18 by: sel22 Page 1 of 1

Figure S18. HR-ESI-MS of **3**.

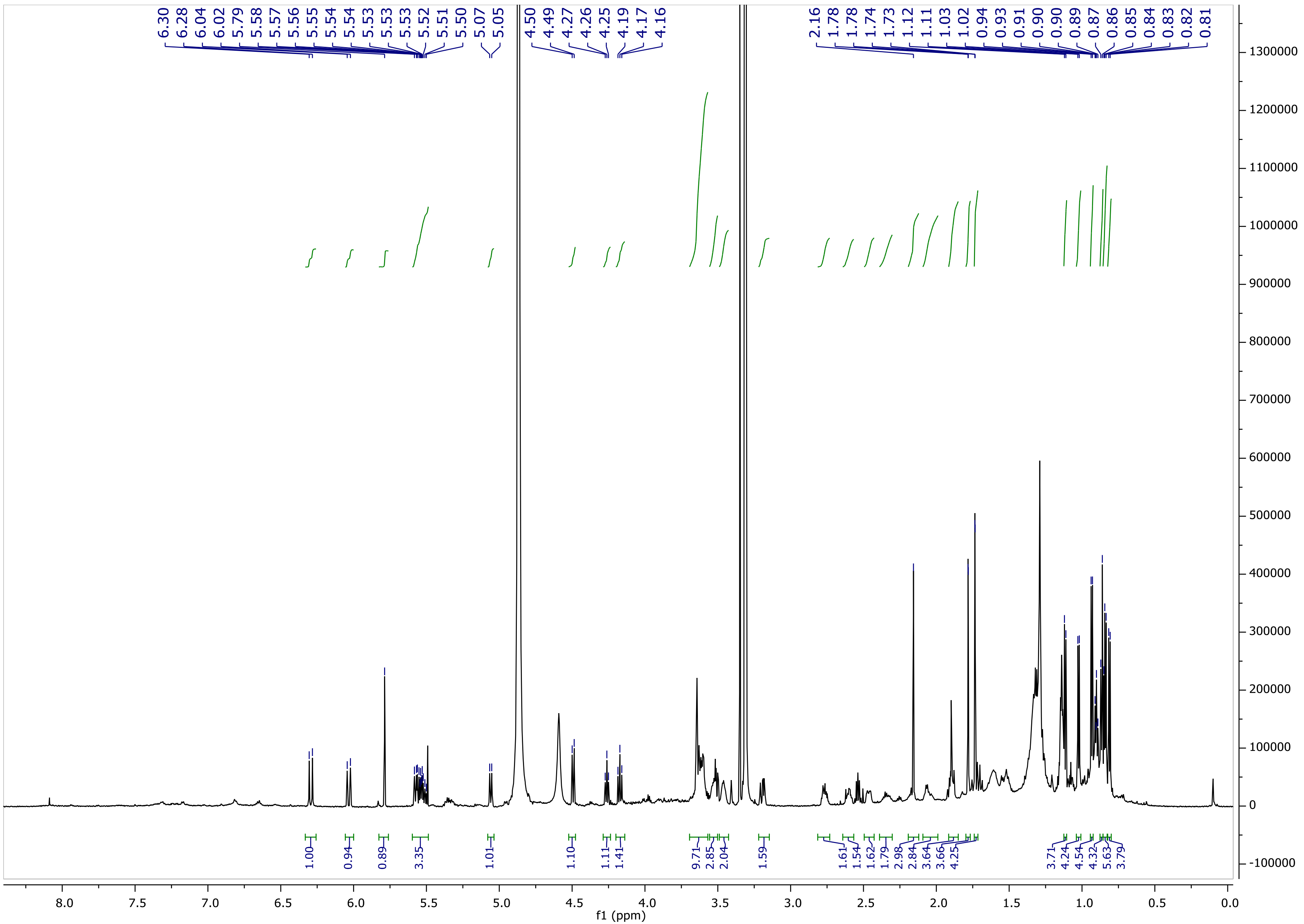

Figure S19. ^{1}H NMR spectrum of **3** in methanol-d_4 at 700 MHz.

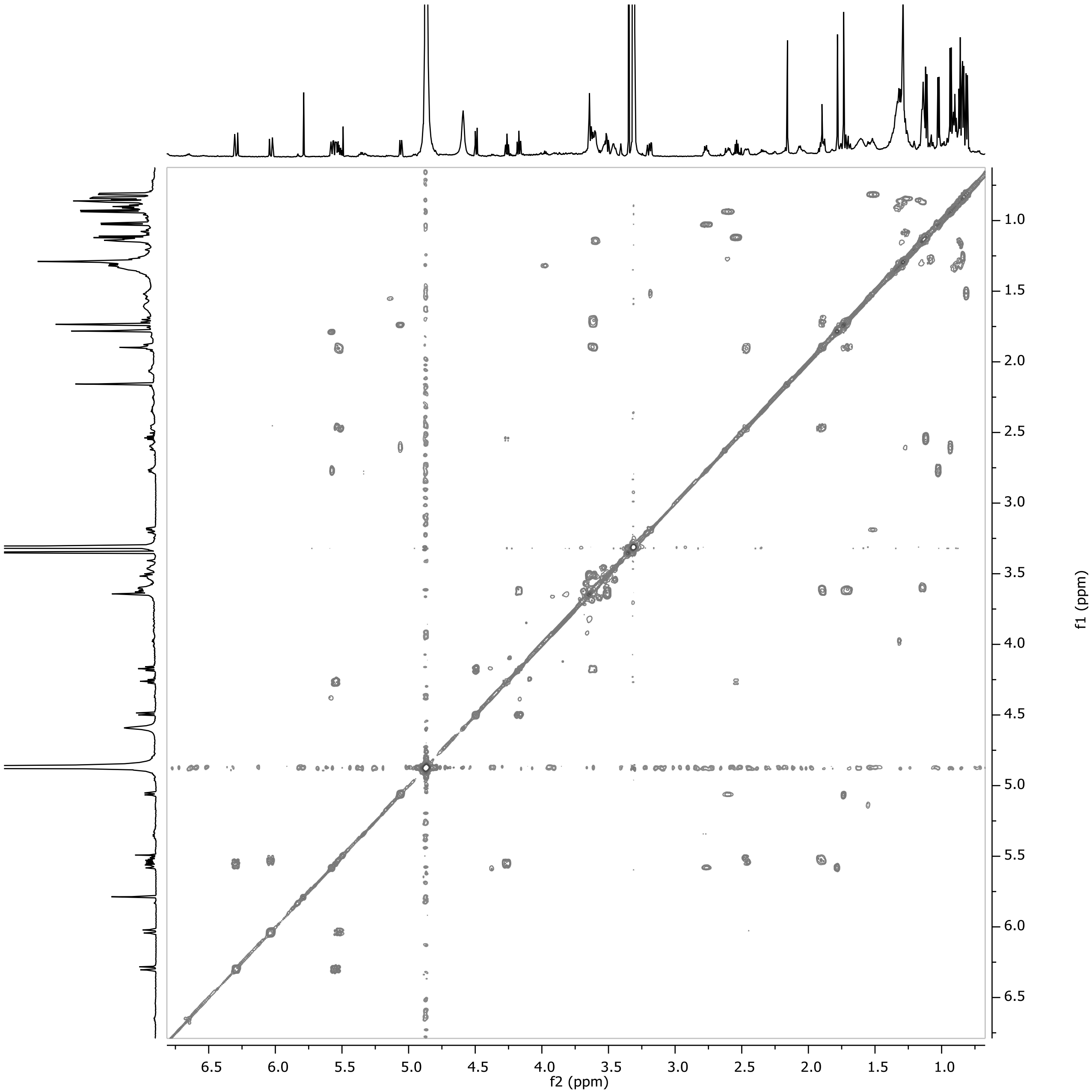

Figure S20. ^{1}H-^{1}H COSY spectrum of **3** in methanol-d_4 at 700 MHz.

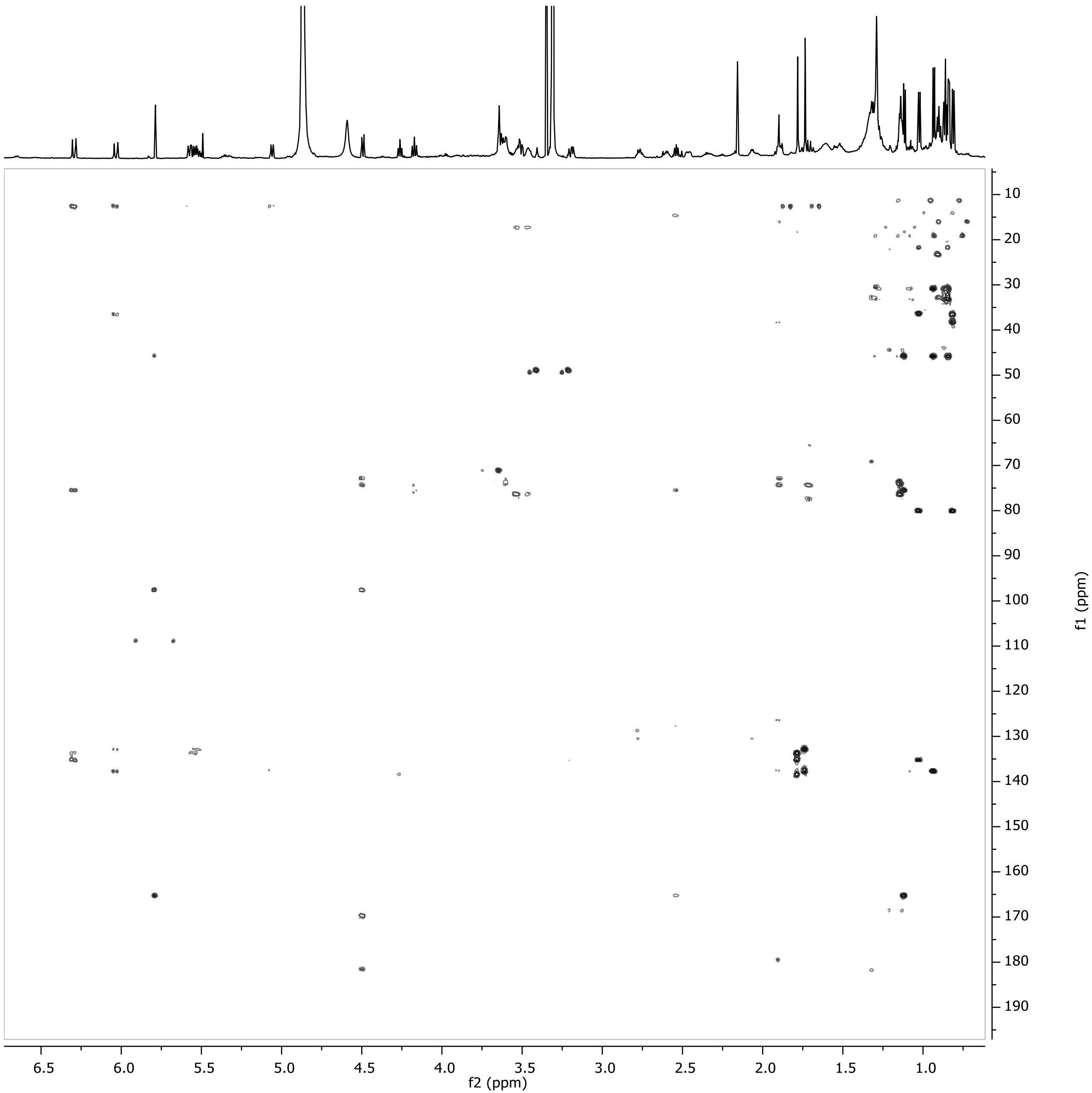

Figure S21. HMBC spectrum of **3** in methanol-d_4 at 700 MHz.

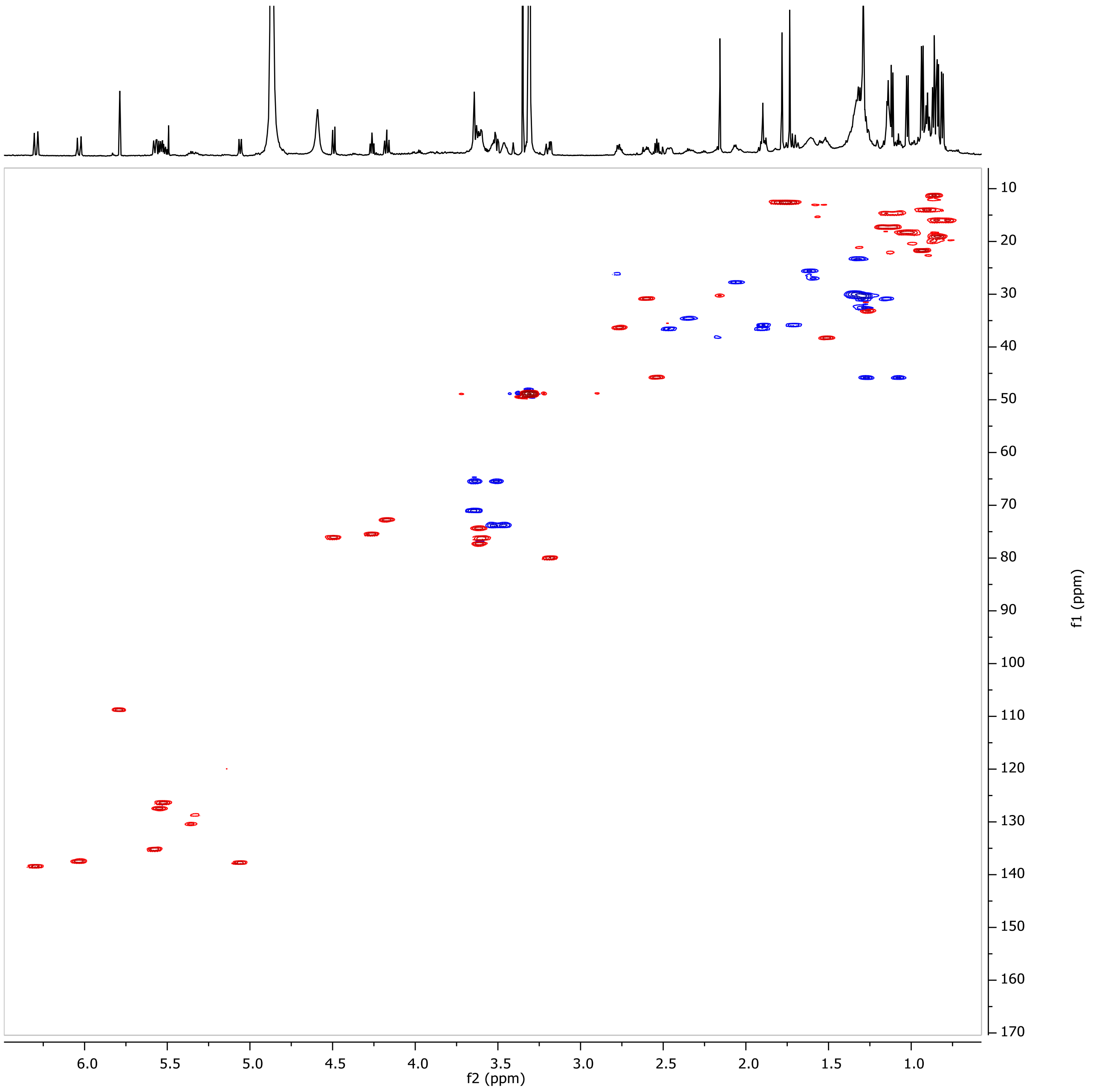

Figure S22. HSQC spectrum of **3** in methanol-d_4 at 700 MHz.

Generic Display Report

Analysis Info

Analysis Name	S:\PEOPLE\cho23_Caren Holzenkamp\NMR\Maxis Data\purified fractions\03-M-MeOH-F6-429	Acquisition Date	03.09.2023 07:27:56
Method	50380.d MyNe-02-03-02-M-MeOH-F6_BC8_01_50380.d	Operator	tti
Sample Name	MyNe-02-03-02-M-MeOH-F6	Instrument	amaZon speed
Comment			

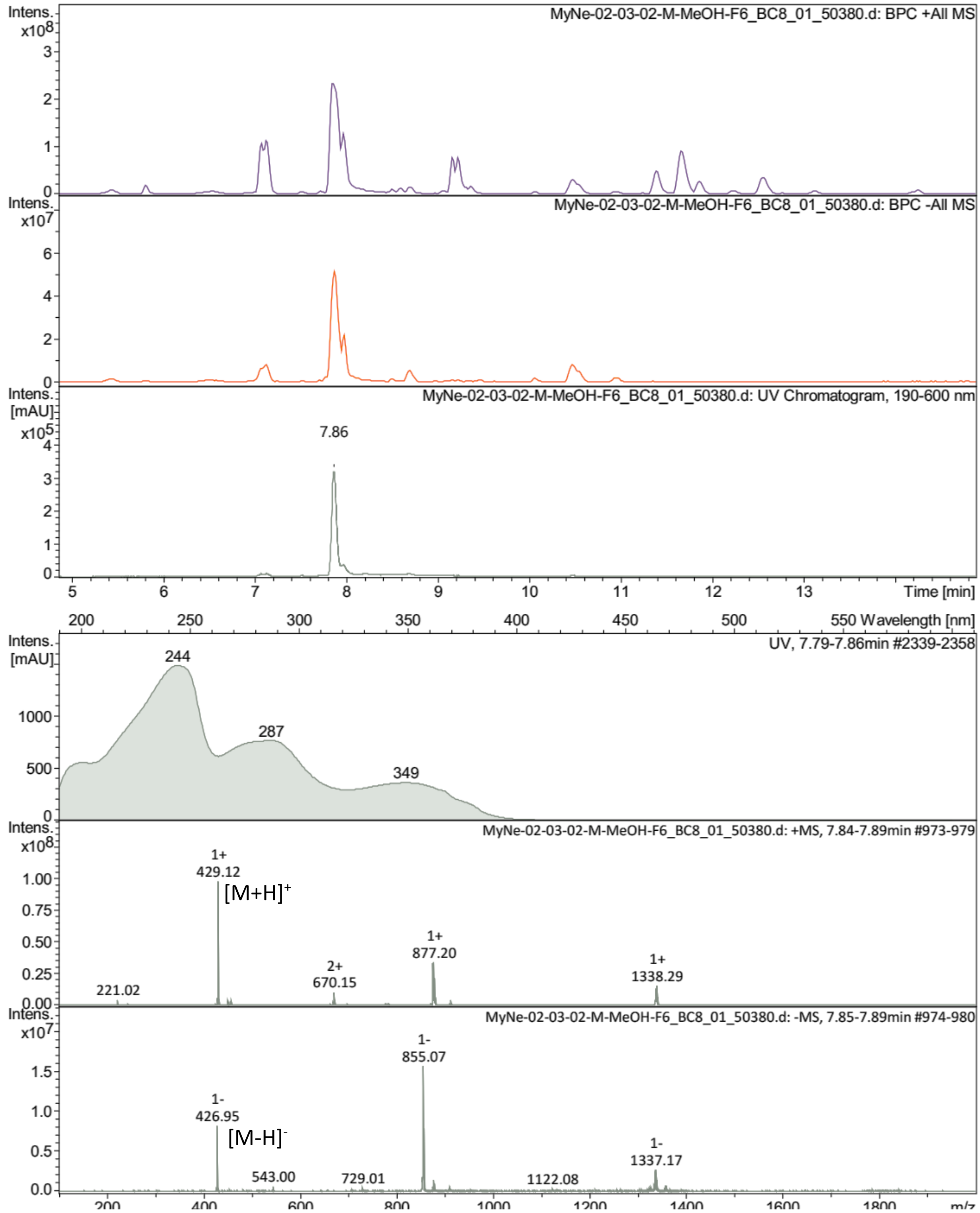

Bruker Compass DataAnalysis 4.4 printed: 11.10.2023 12:57:37 by: sel22 Page 1 of 1

Figure S23. LR-ESI-MS of **4**.

Generic Display Report

Analysis Info

Analysis Name	S:\PEOPLE\cho23_Caren Holzenkamp\NMR\Maxis Data\purified fractions\03-M-MeOH-F6-429	Acquisition Date	27.09.2023 09:36:11
Method	[illegible]	Operator	ate06
Sample Name	MyNe-02-03-02-M-MeOH-F6	Instrument	maXis
Comment	Screening01<br>Waters Acquity UPLC BEH C_18 1,7um 2.1x50mm		

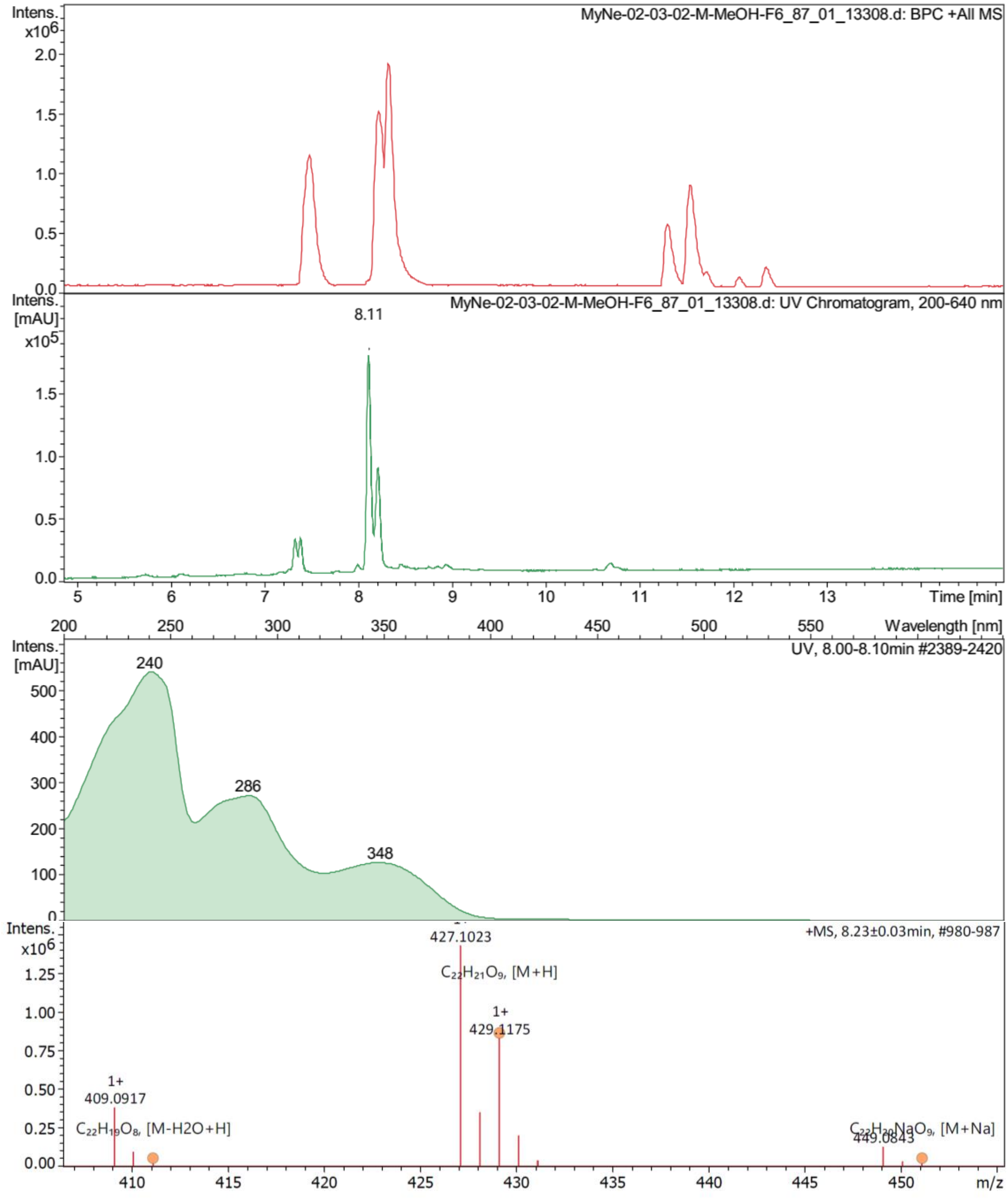

Figure S24. HR-ESI-MS of **4**.

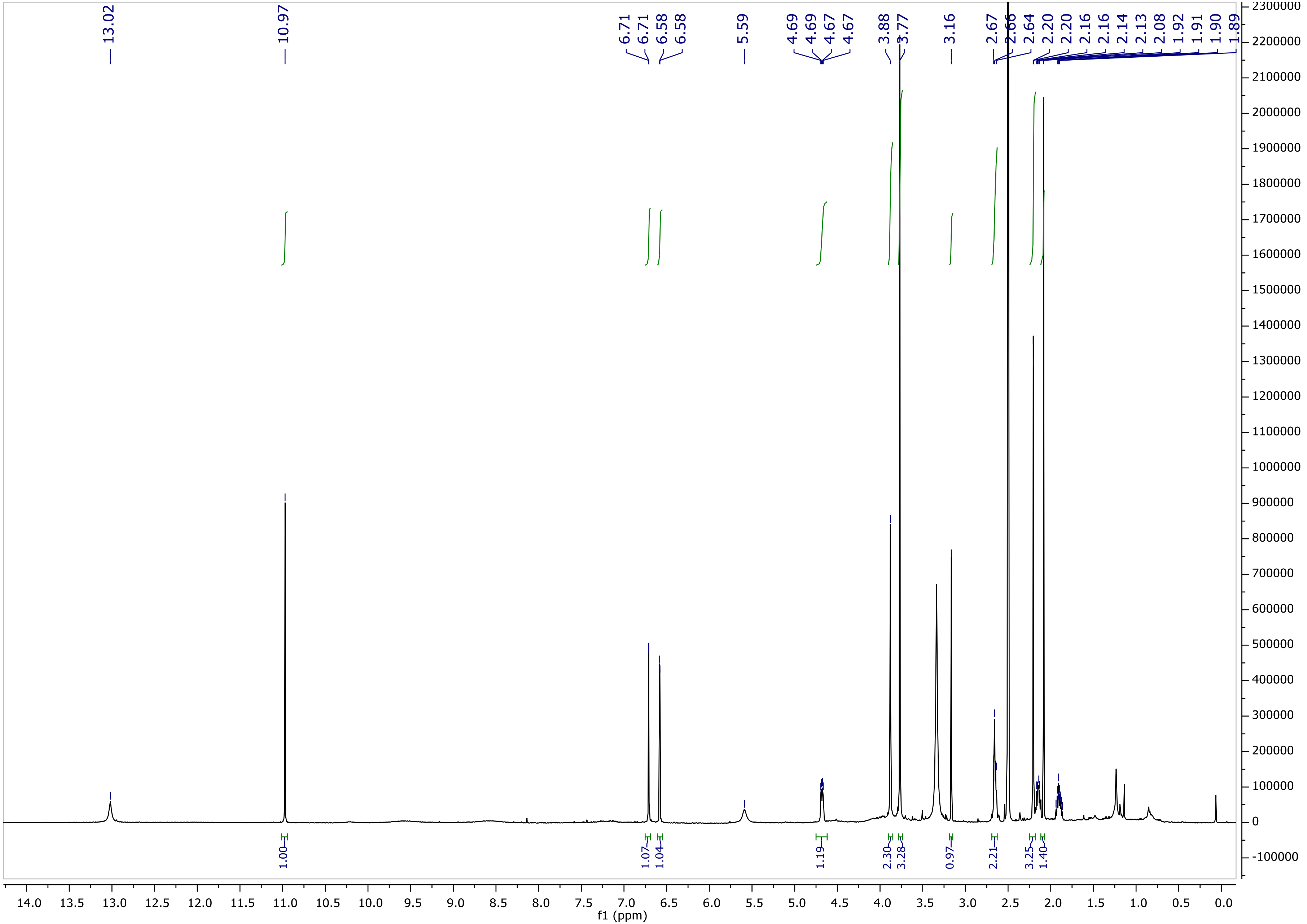

Figure S25. ^{1}H NMR spectrum of **4** in DMSO-d_6 at 500 MHz.

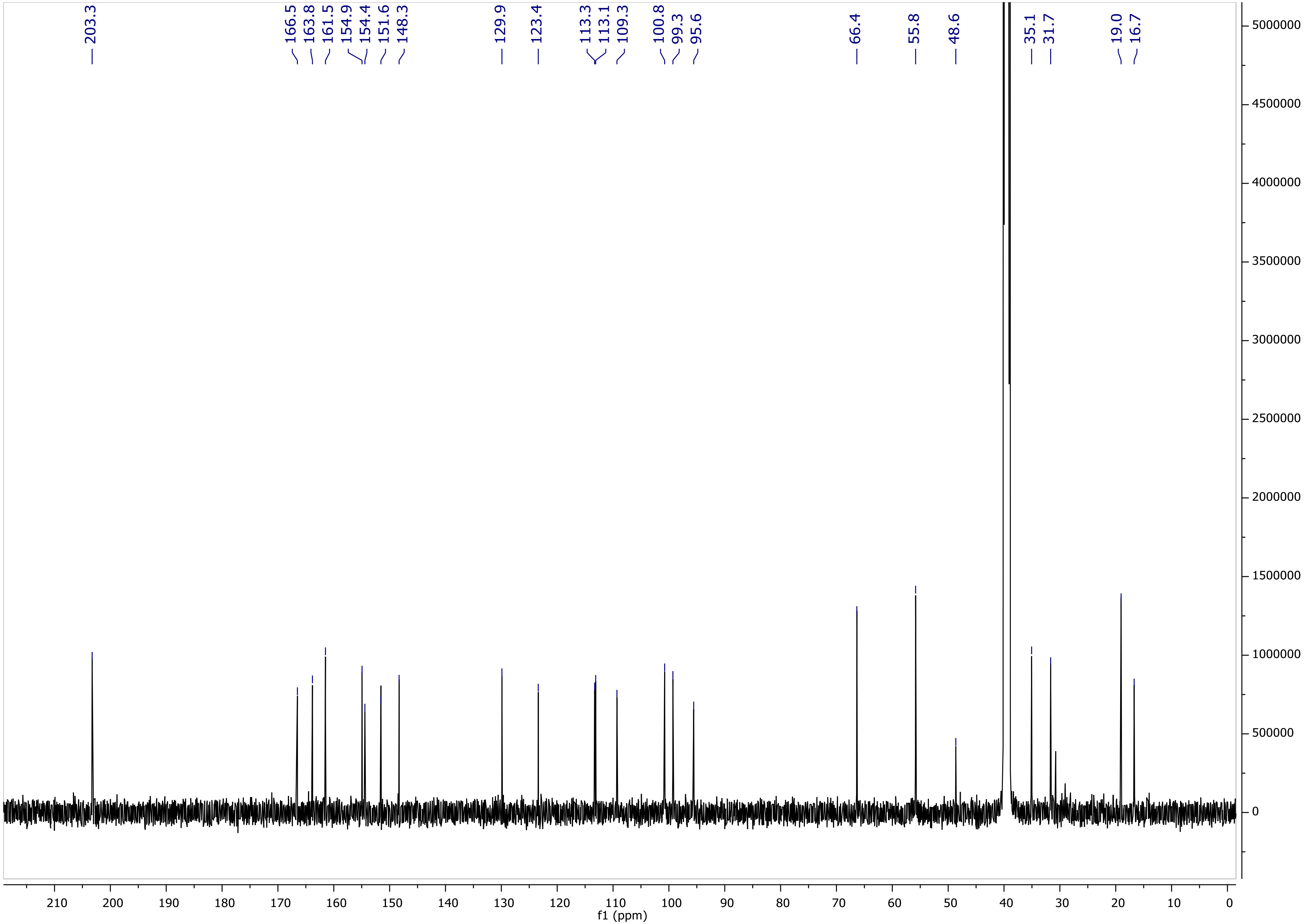

Figure S26. ^{13}C NMR spectrum of **4** in DMSO-d_6 at 125 MHz.

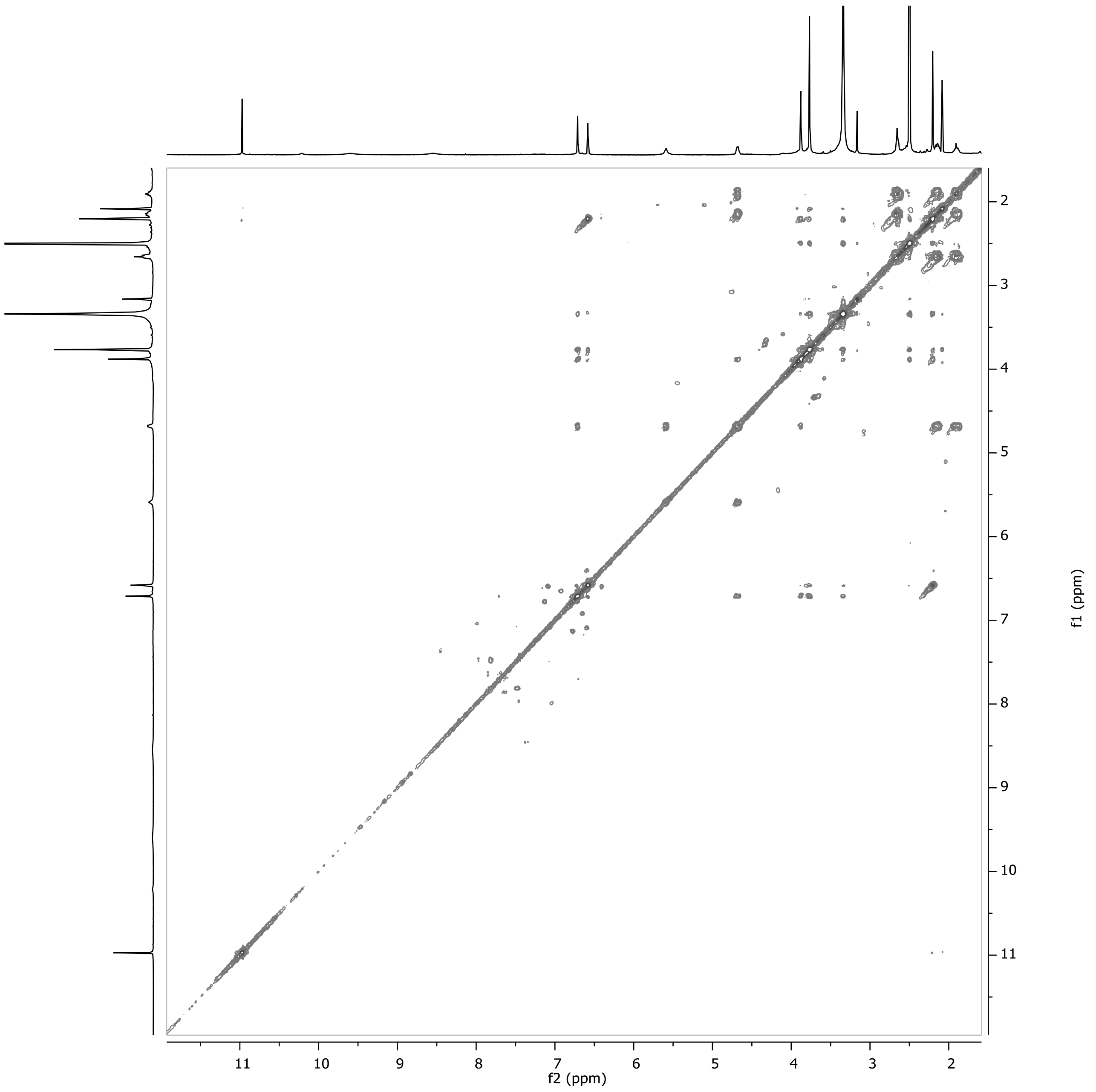

Figure S27. ^{1}H-^{1}H COSY spectrum of **4** in DMSO-d_6 at 500 MHz.

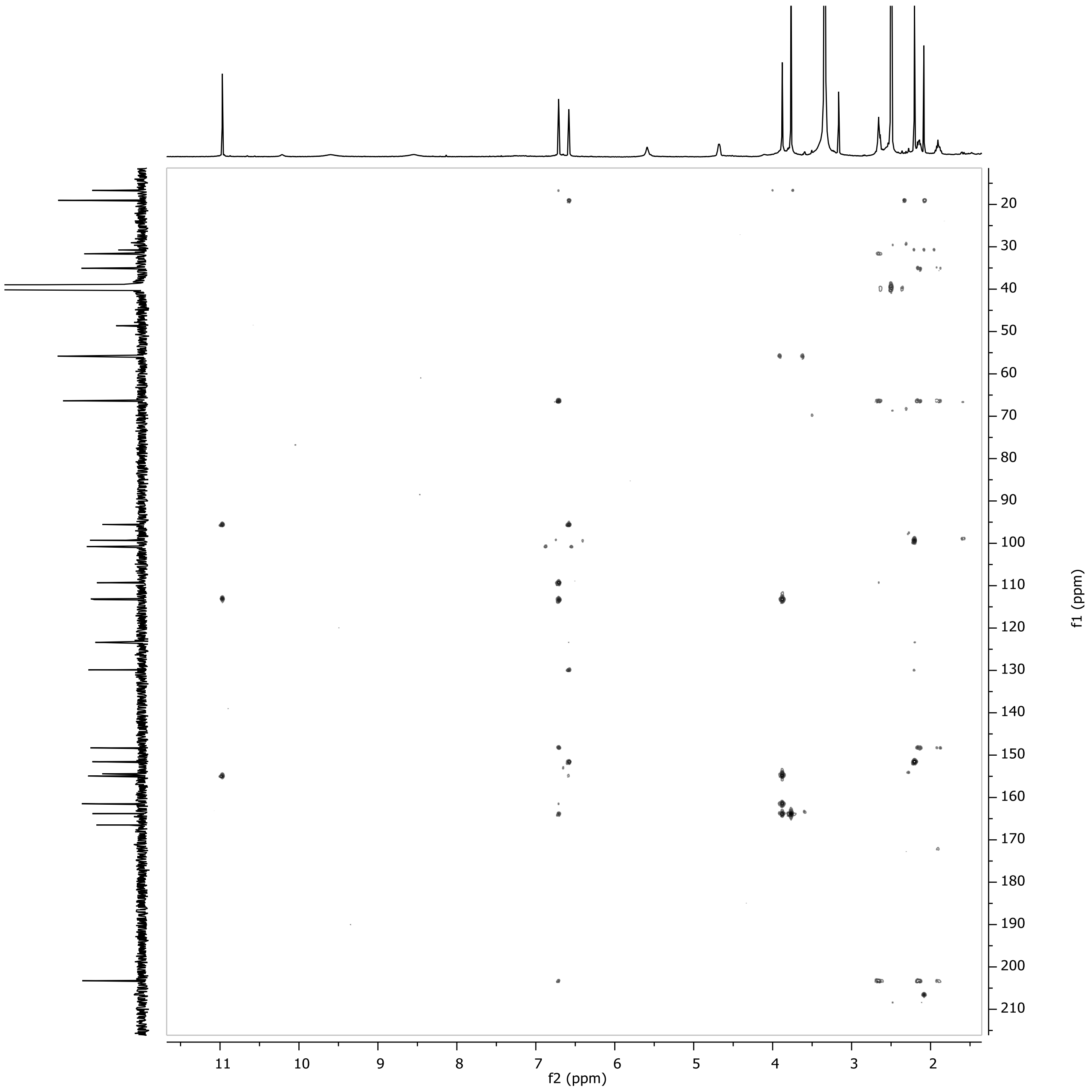

Figure S28. HMBC spectrum of **4** in DMSO-d_6 at 500 MHz.

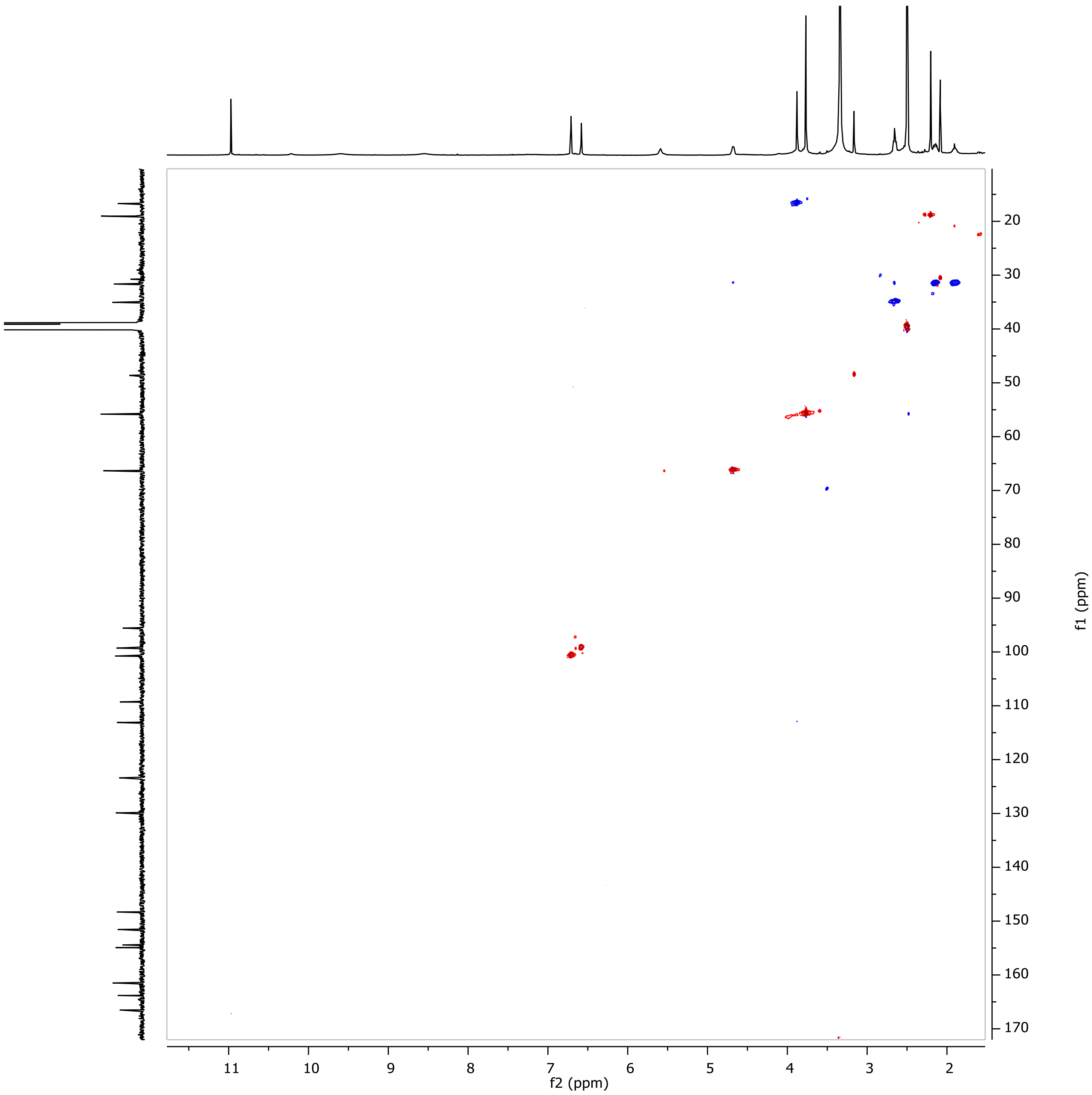

Figure S29. HSQC spectrum of **4** in DMSO-d_6 at 500 MHz.

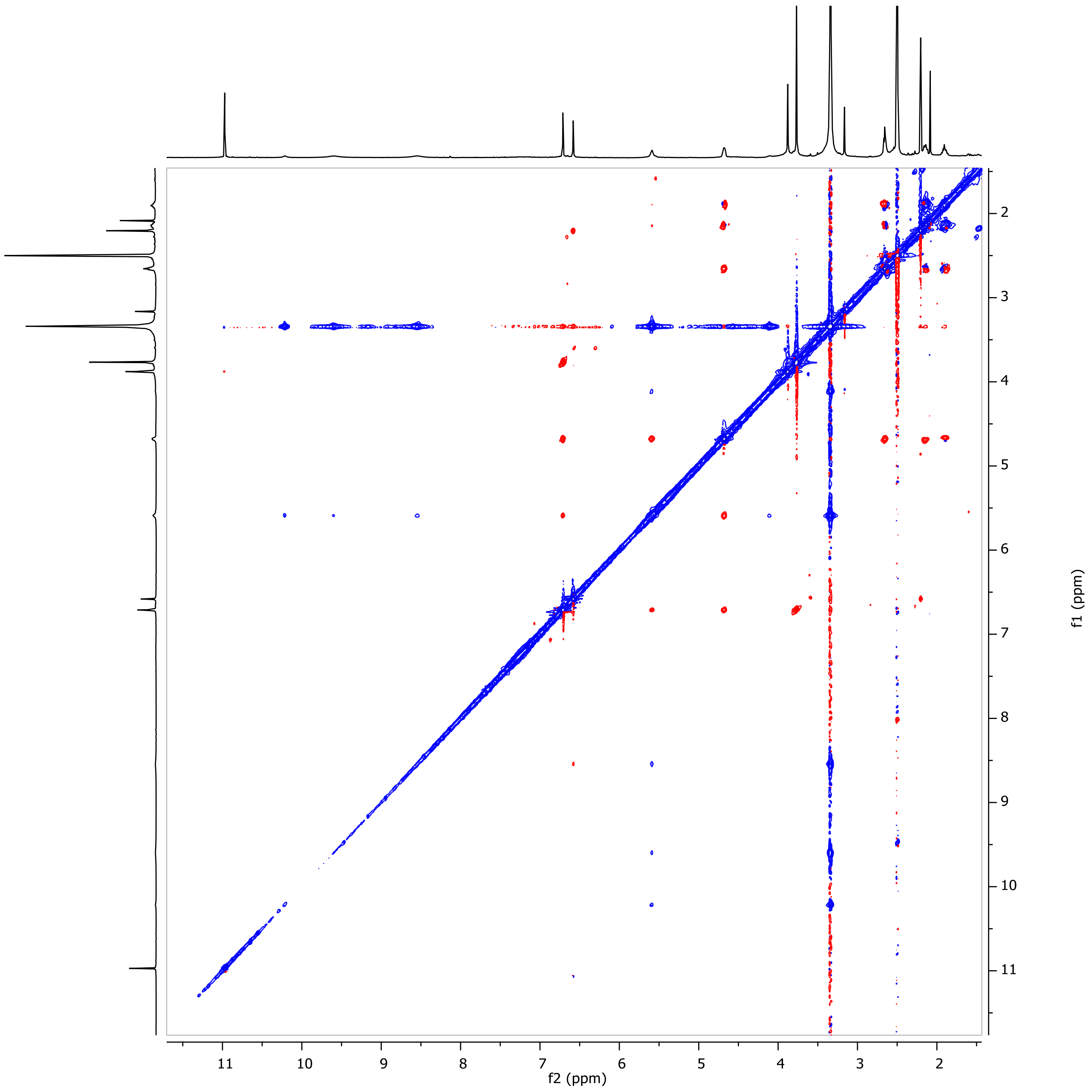

Figure S30. ROESY spectrum of **4** in DMSO-d_6 at 700 MHz.

Display Report

Analysis Info

Acquisition Date 21.10.2023 10:07:45

Analysis Name S:\PEOPLE\cho23_Caren Holzenkamp\NMR\MS-Data\purified fractions\MeOH-F14-F7-276\MyNe-03-02-06-MeOH-F3+..-F14-F7_GE5_01_15351.d

Method 15351.m

Operator lab

Sample Name MyNe-03-02-06-MeOH-F3+..-F14-F7

Instrument amaZon speed 8374444.06119

Comment

Acquisition Parameter

Ion Polarity Negative

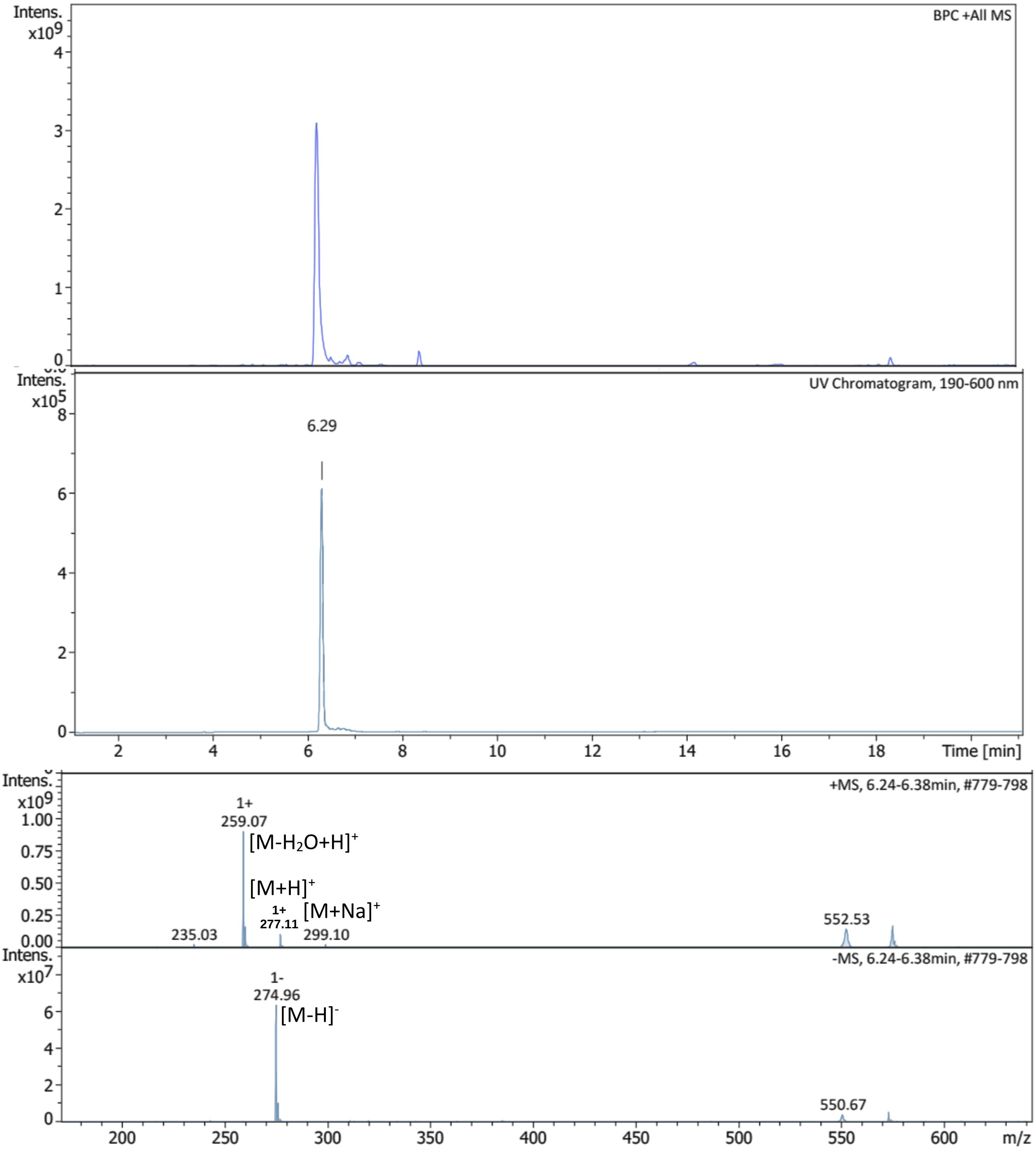

Figure S31. LR-ESI-MS of **5**.

Display Report

Analysis Info

Analysis Name	S:\PEOPLE\cho23_Caren Holzenkamp\NMR\MS-Data\purified fractions\MeOH-F14-F7-276\MyNe-03-02-06-MeOH-F3+F4+F6+F7-F14-F6_P1-B-3_1_905.d	Acquisition Date	03.11.2023 04:50:50
Method	MWIS_BEH50mm_25min_ohne tims.m	Operator	Demo User
Sample Name	MyNe-03-02-06-MeOH-F3+F4+F6+F7-F14-F6	Instrument	timsTOF Pro 2 1875087.10646
Comment			

Acquisition Parameter

Source Type	ESI	Ion Polarity	Positive	Set Nebulizer	1.0 Bar
Focus	Active	Set Capillary	4000 V	Set Dry Heater	200 °C
Scan Begin	150 m/z	Set End Plate Offset	-500 V	Set Dry Gas	5.0 l/min
Scan End	2500 m/z	Set Collision Cell RF	1000.0 Vpp	Set Divert Valve	Waste

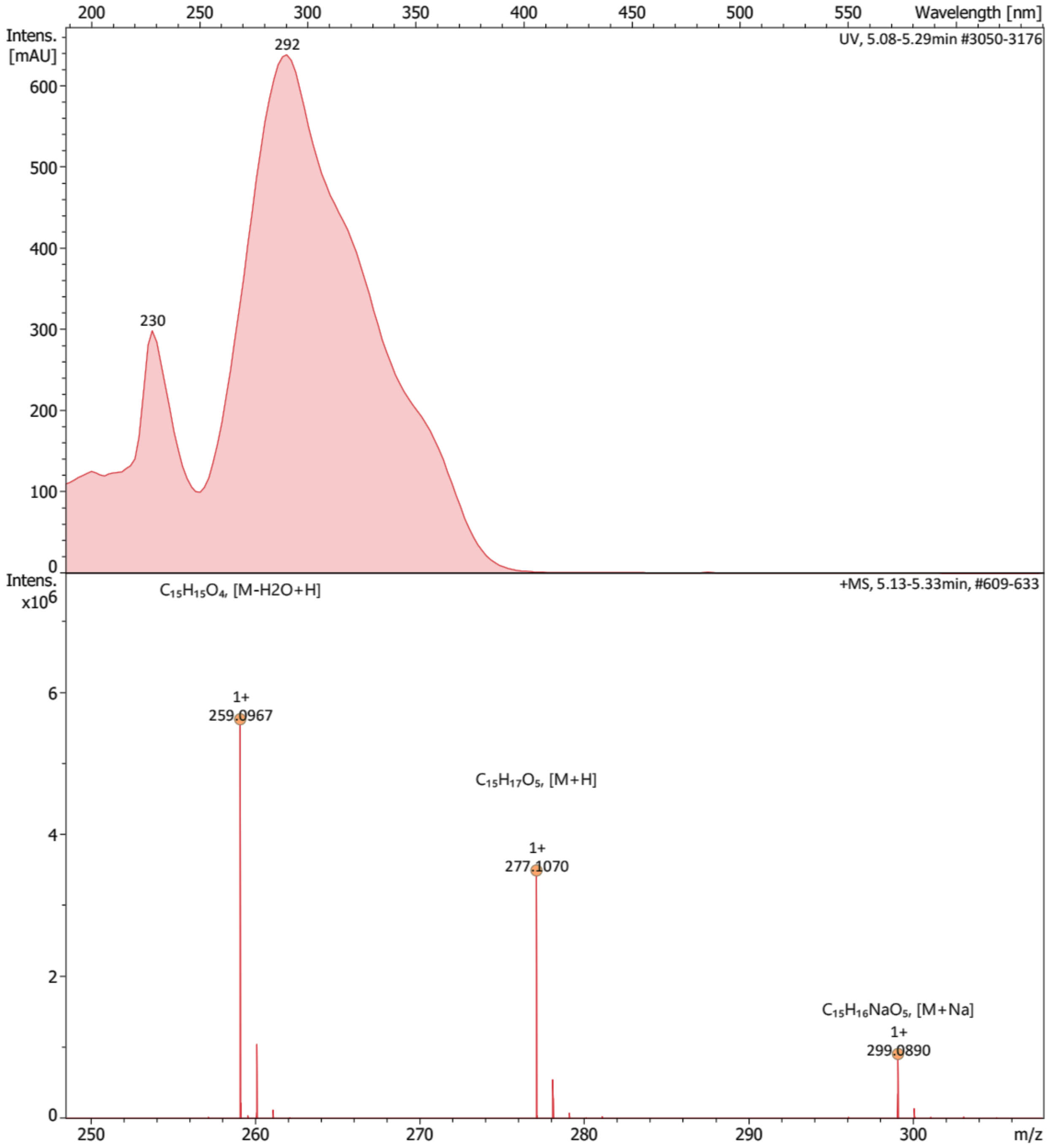

Bruker Compass DataAnalysis 6.1 printed: 09.11.2023 06:07:27 by: sel22 Page 1 of 1

Figure S32. HR-ESI-MS of **5**.

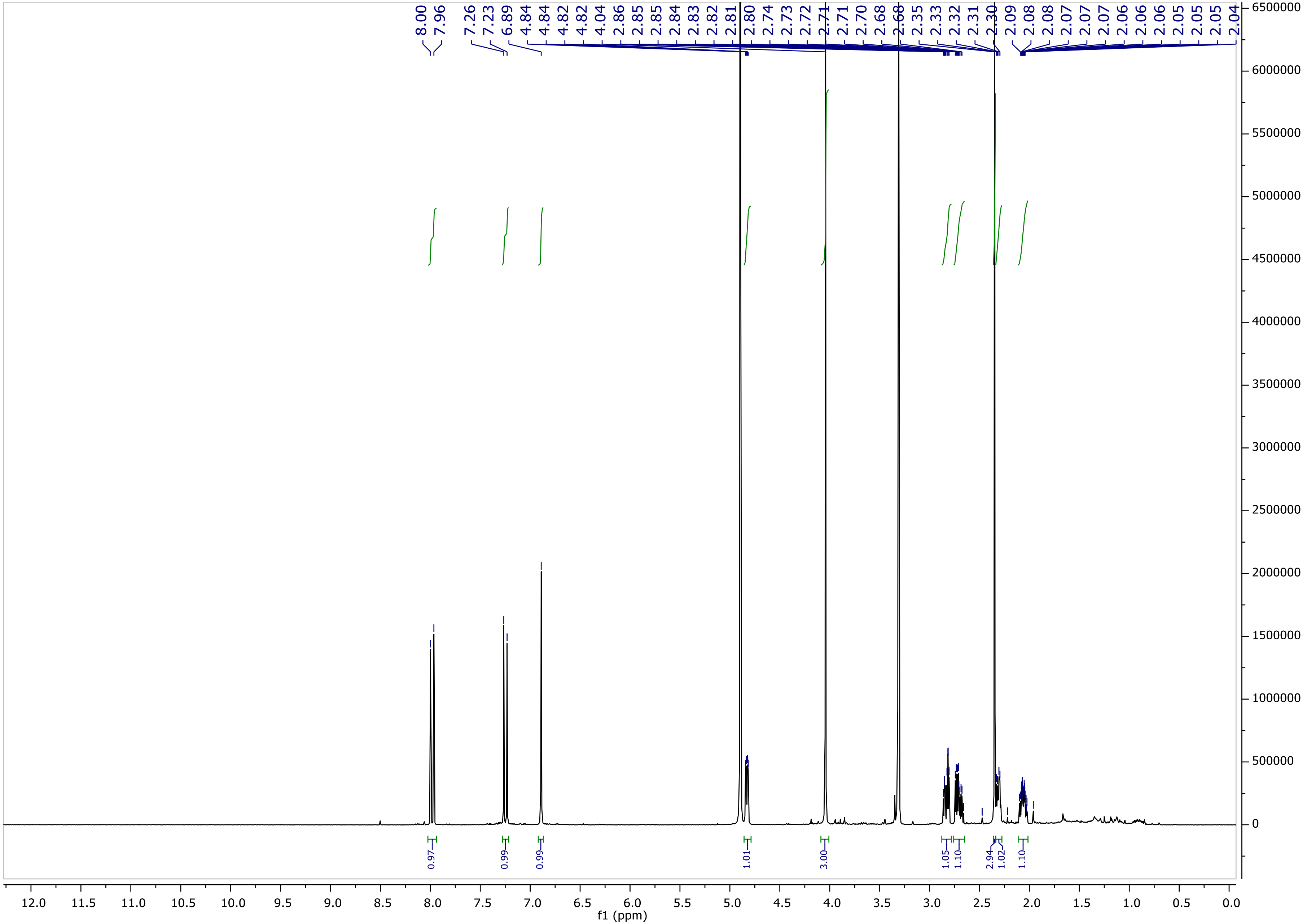

Figure S33. ^{1}H NMR spectrum of **5** in methanol-d_4 at 500 MHz.

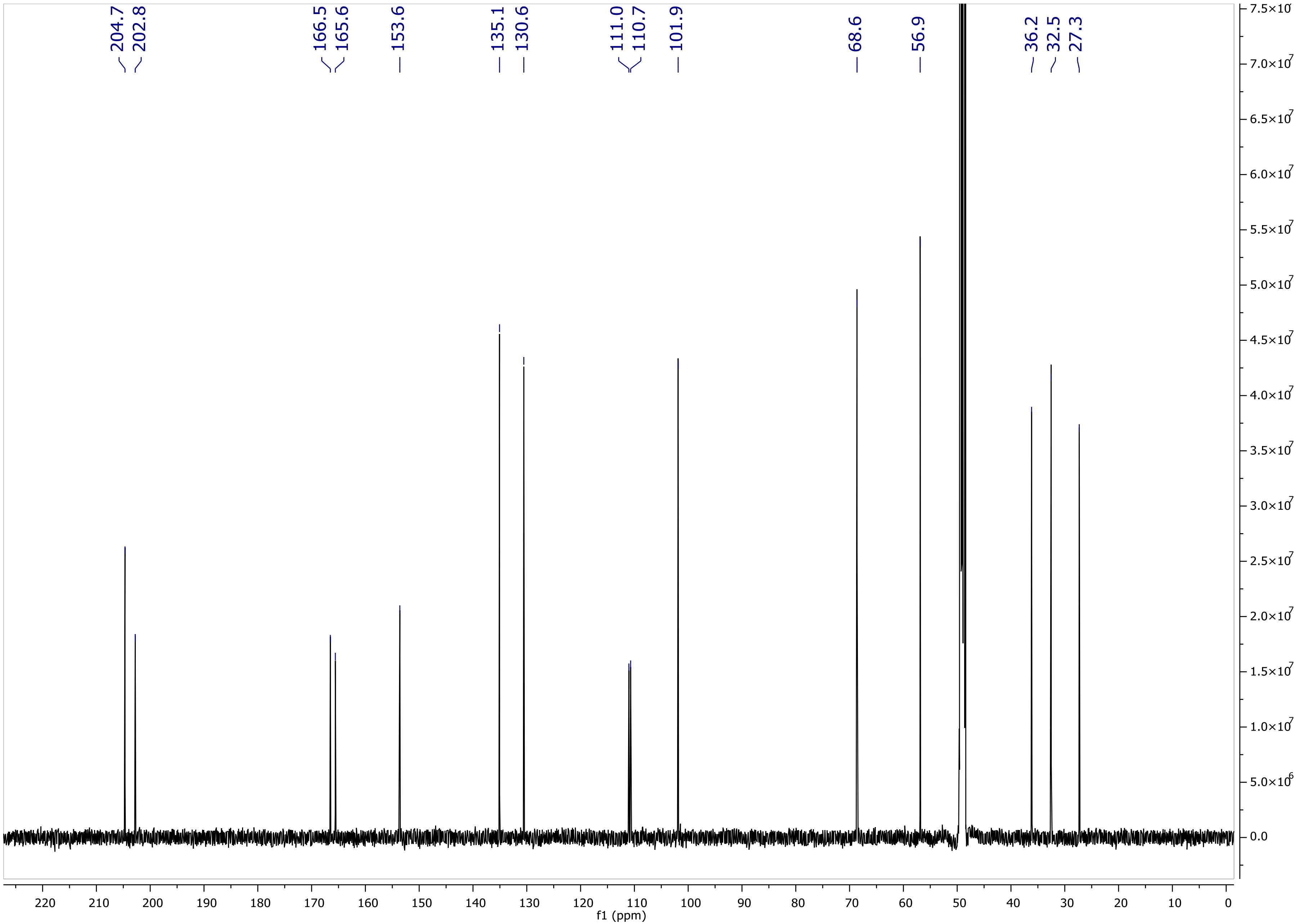

Figure S34. ^{13}C NMR spectrum of **5** in methanol-d_4 at 125 MHz.

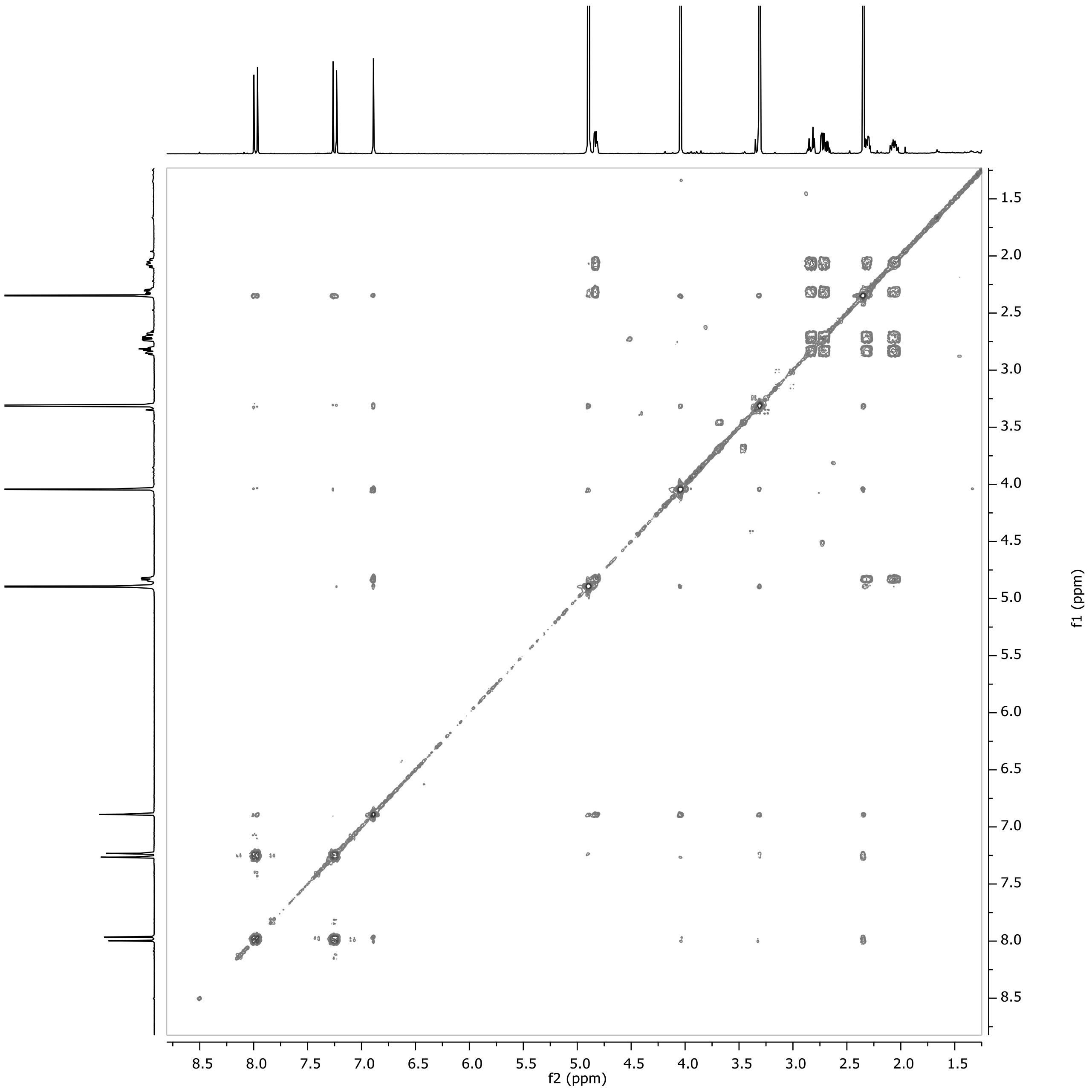

Figure S35. ^{1}H-^{1}H COSY spectrum of **5** in methanol-d_4 at 500 MHz.

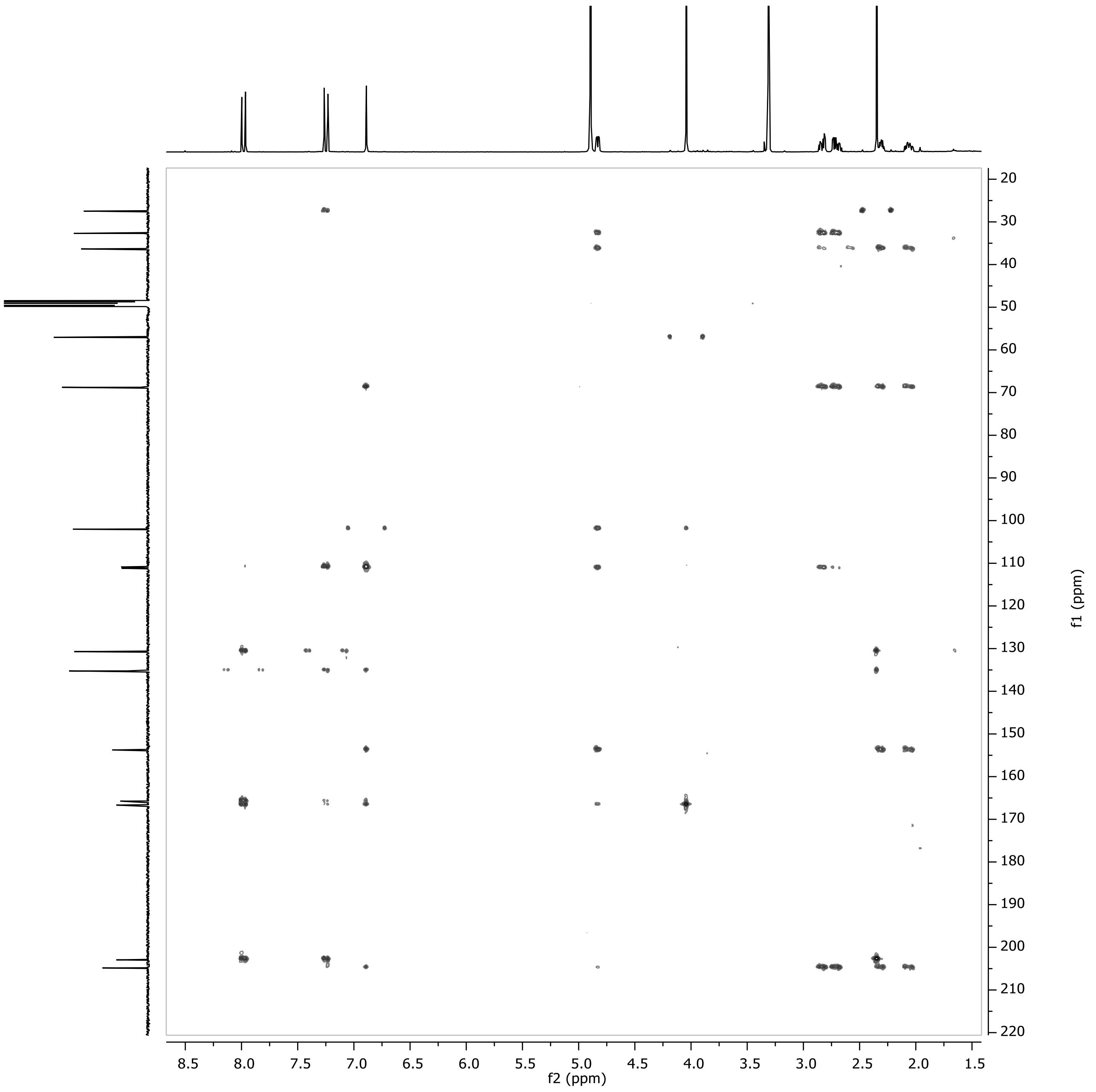

Figure S36. HMBC spectrum of **5** in methanol-d_4 at 500 MHz.

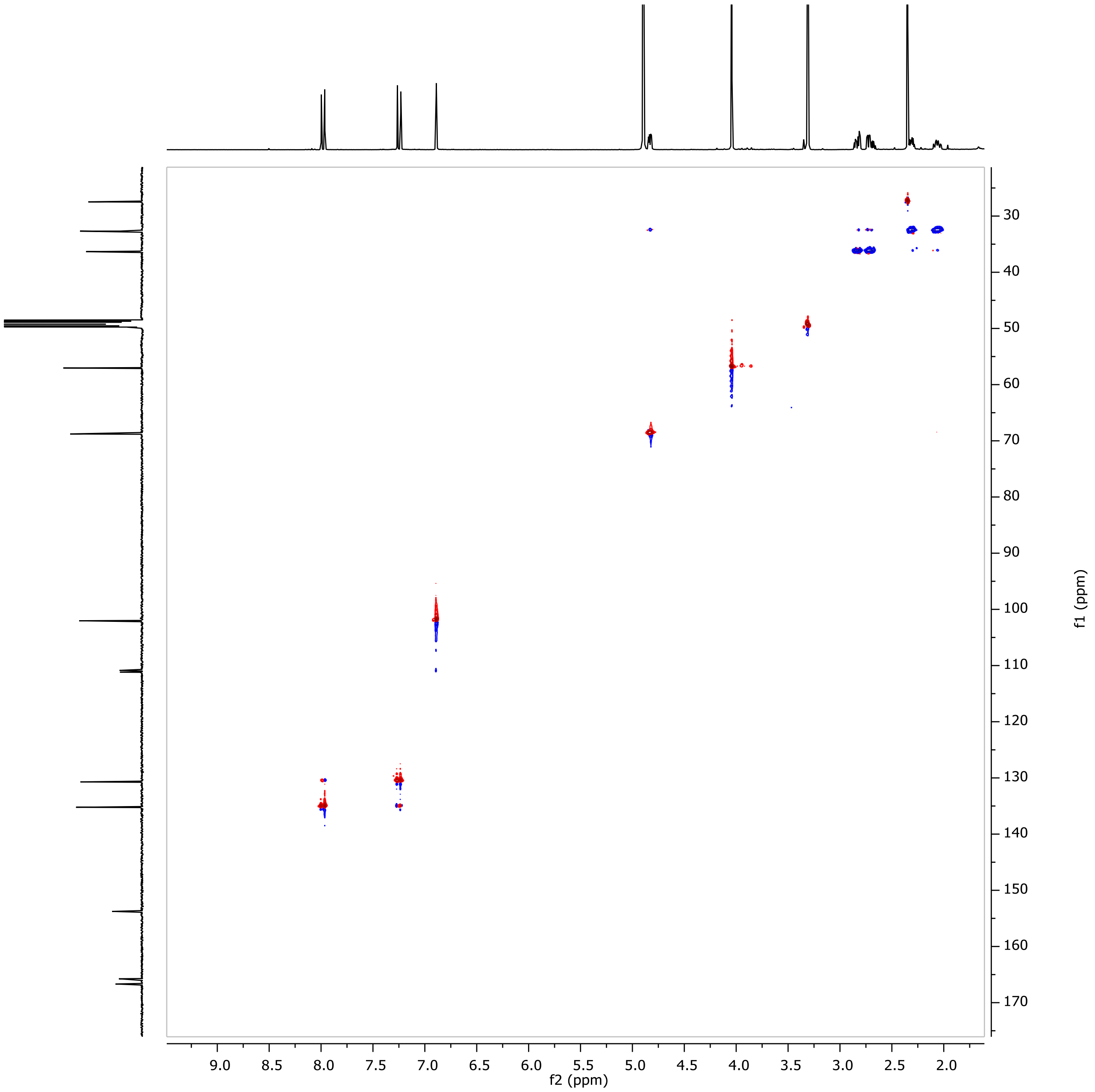

Figure S37. HSQC spectrum of **5** in methanol-d_4 at 500 MHz.

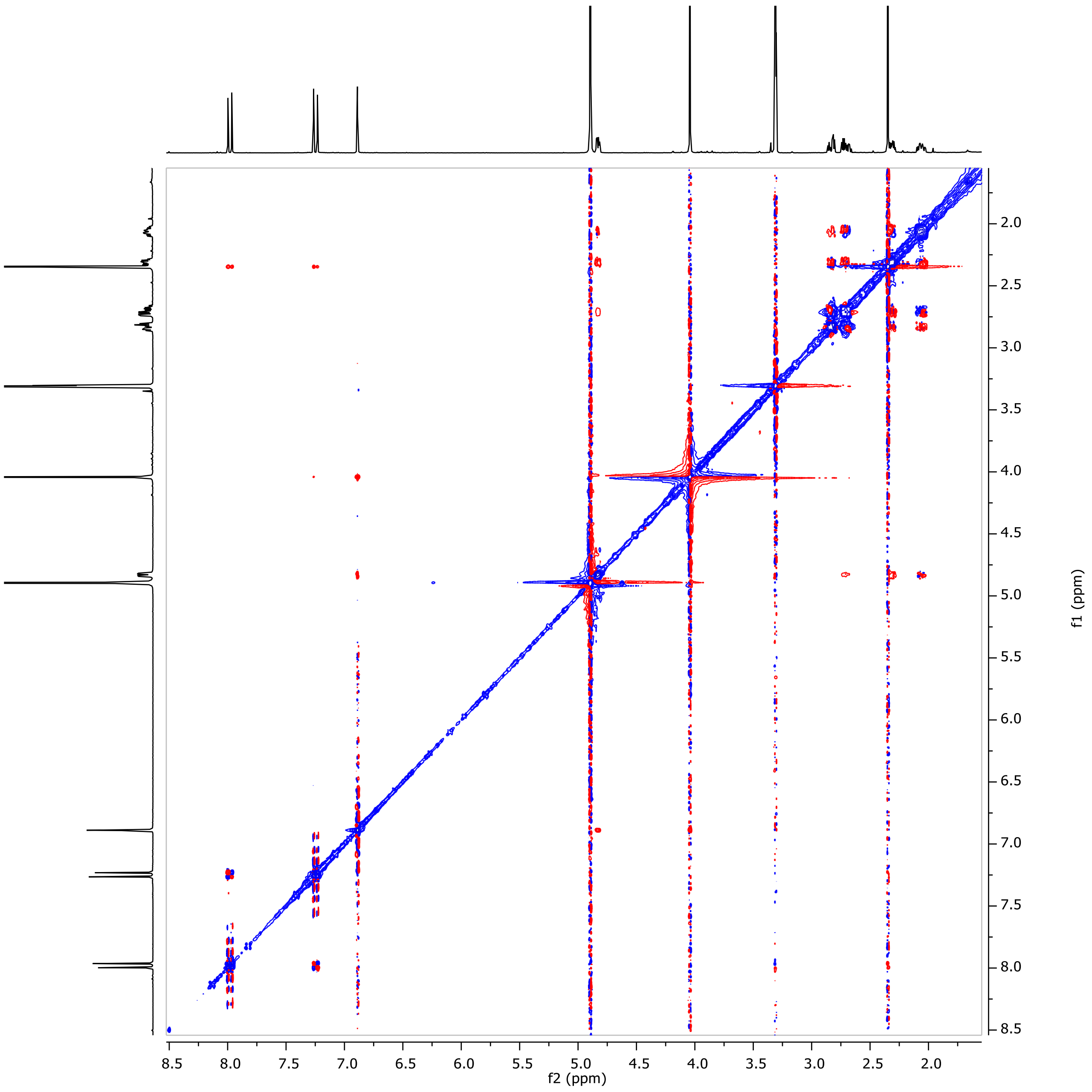

Figure S38. ROESY spectrum of **5** in methanol-d_4 at 500 MHz.

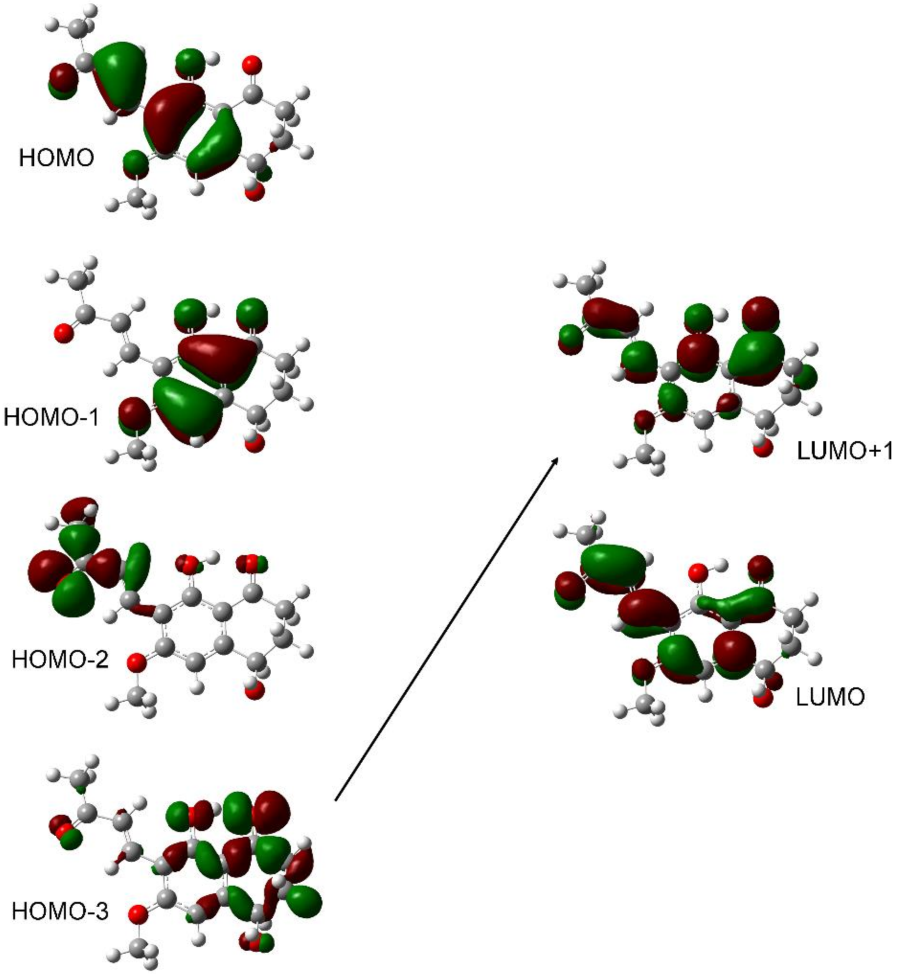

Figure S39. Kohn-Sham orbitals of (*R*)-**5** computed at the BH&HLYP/TZVP PCM/MeOH level for the lowest-energy ωB97X/TZVP PCM/MeOH conformer. Major contributors of the first four orbitals: 1. HOMO-2 → LUMO, 2. HOMO → LUMO, 3. HOMO-1 → LUMO, 4. HOMO-3 → LUMO+1. The arrow indicates the fourth one corresponding to the n-π* transition of the tetralone chromophore.

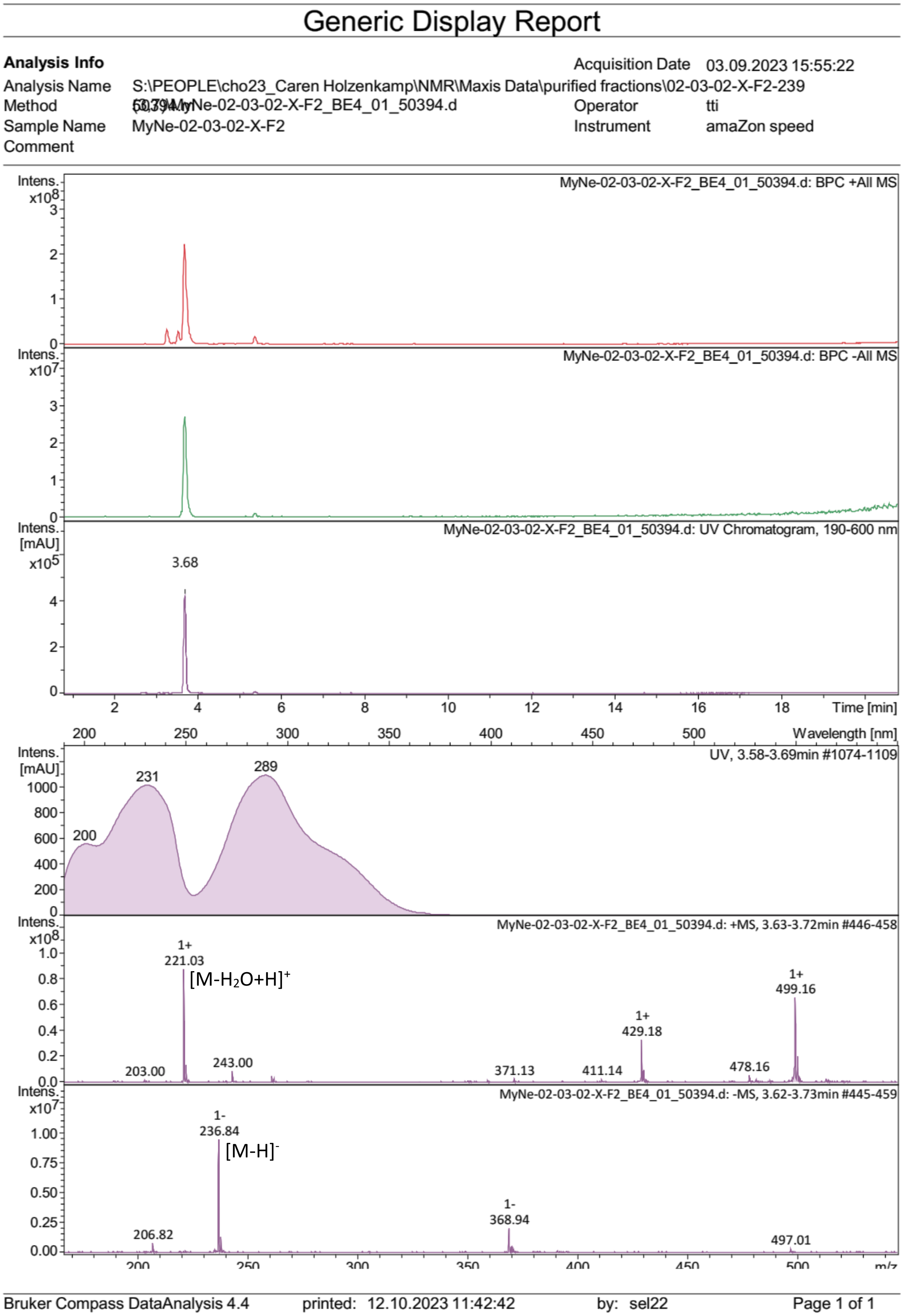

Figure S40. LR-ESI-MS of **6**.

Generic Display Report

Analysis Info

Analysis Name	S:\PEOPLE\cho23_Caren Holzenkamp\NMR\Maxis Data\purified fractions\02-03-02-X-F2-239	Acquisition Date	27.09.2023 08:34:18
Method	[illegible]_line.m	Operator	ate06
Sample Name	MyNe-02-03-02-X-F2	Instrument	maXis
Comment	Screening01<br>Waters Acquity UPLC BEH C_18 1,7um 2.1x50mm		

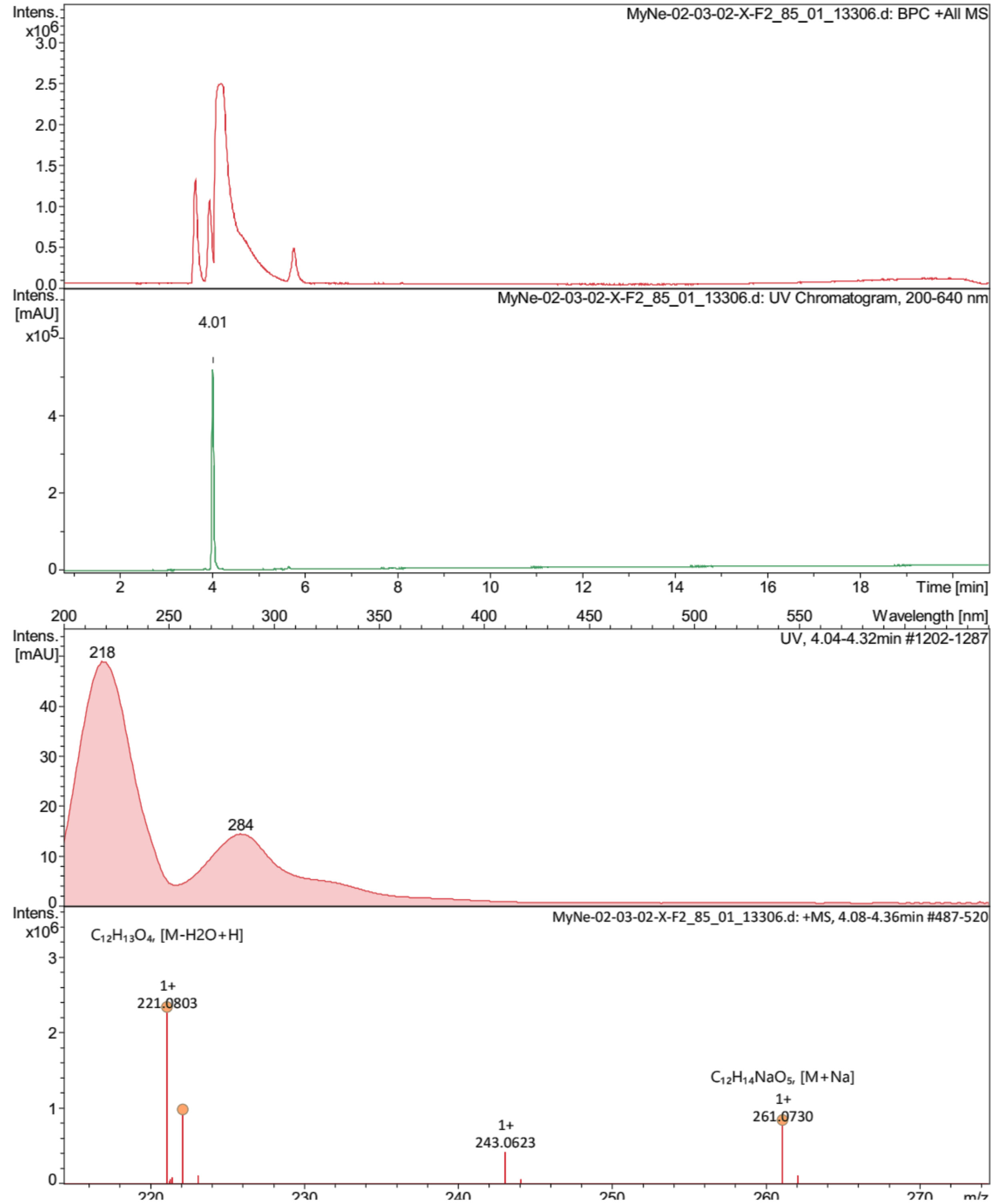

Bruker Compass DataAnalysis 4.4 printed: 12.10.2023 12:23:20 by: sel22 Page 1 of 1

Figure S41. HR-ESI-MS of **6**.

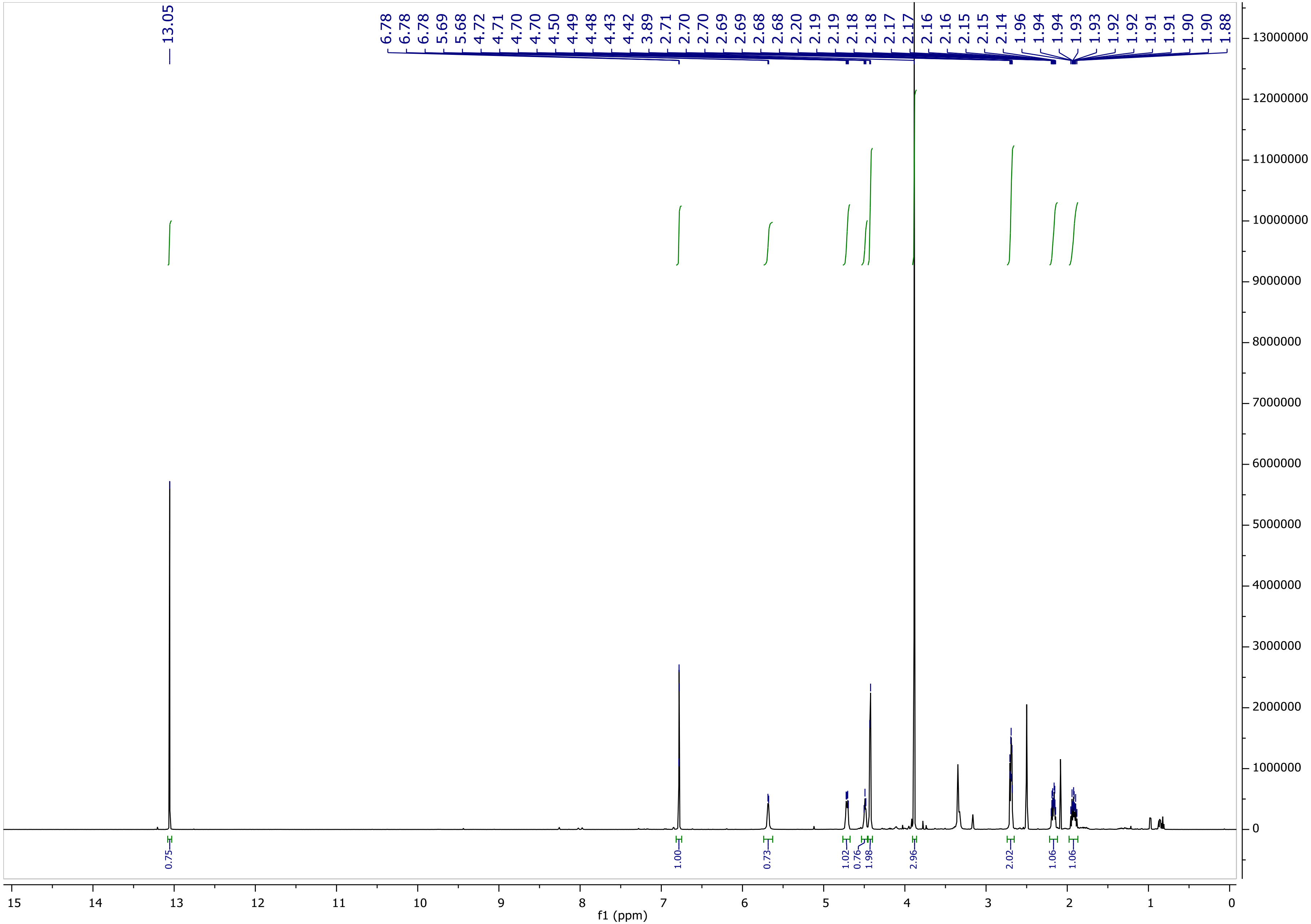

Figure S42. ^{1}H NMR spectrum of **6** in DMSO-d_6 at 500 MHz.

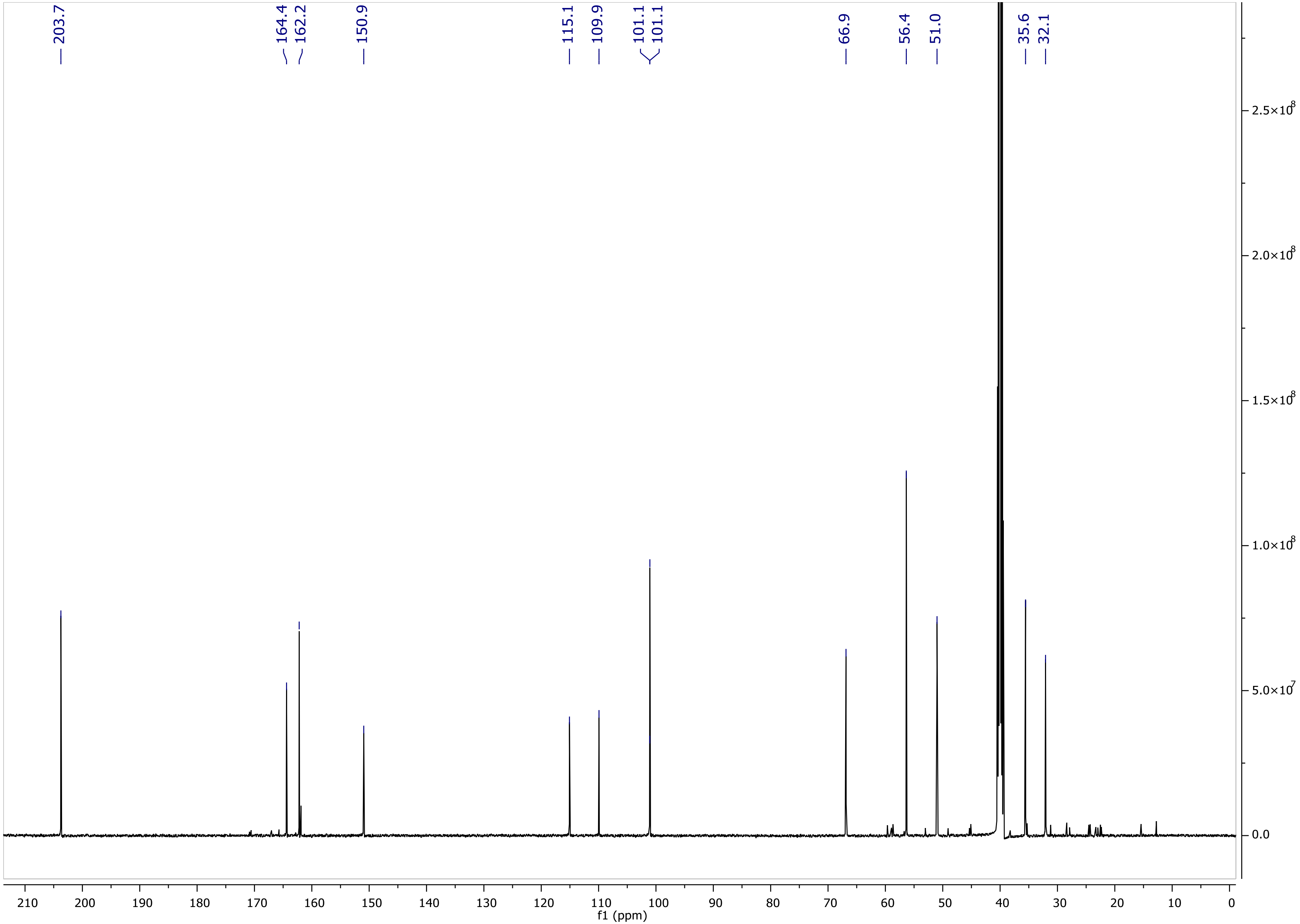

Figure S43. ^{13}C NMR spectrum of **6** in DMSO-d_6 at 125 MHz.

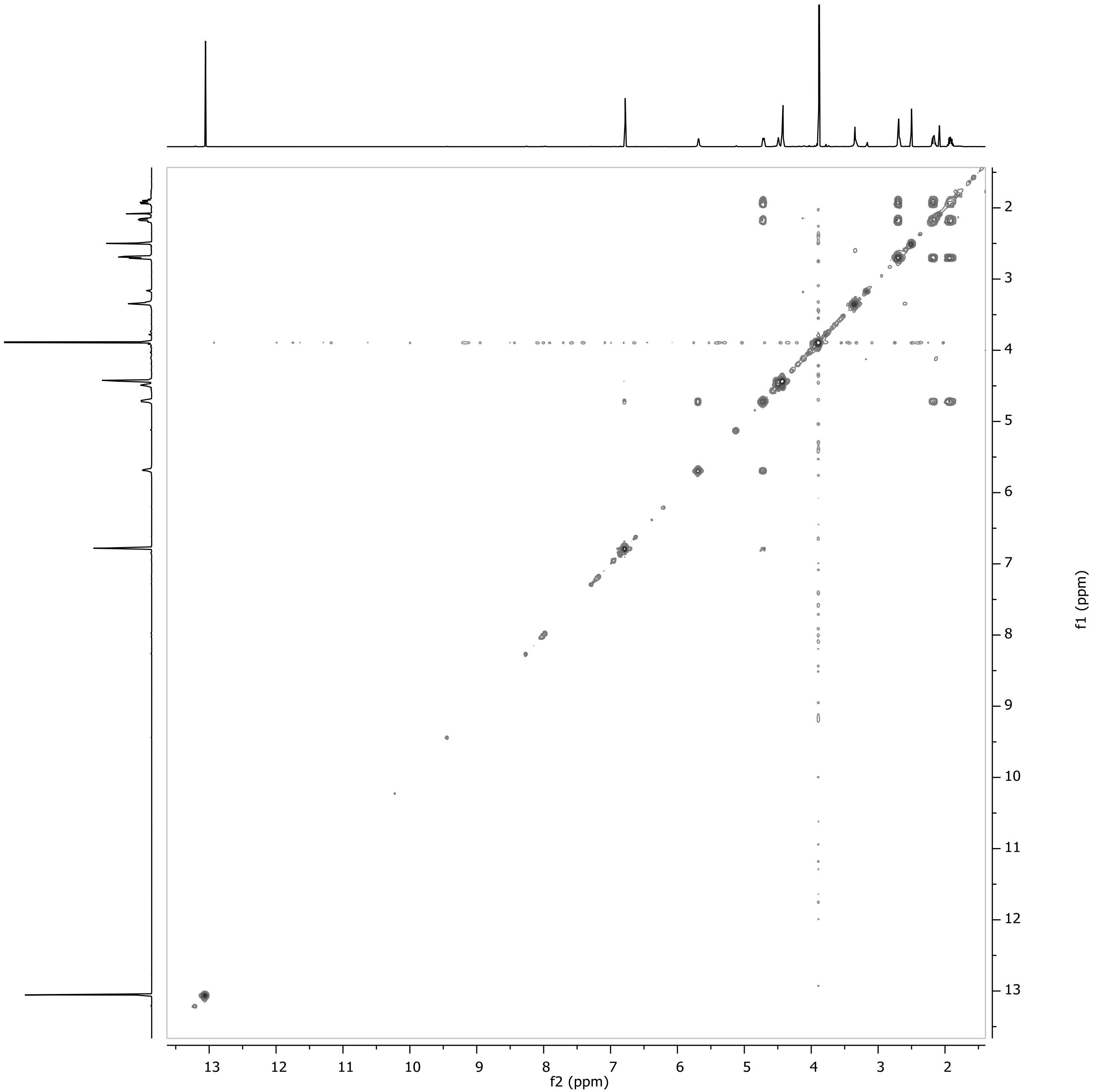

Figure S44. ^{1}H-^{1}H COSY spectrum of **6** in DMSO-d_6 at 500 MHz.

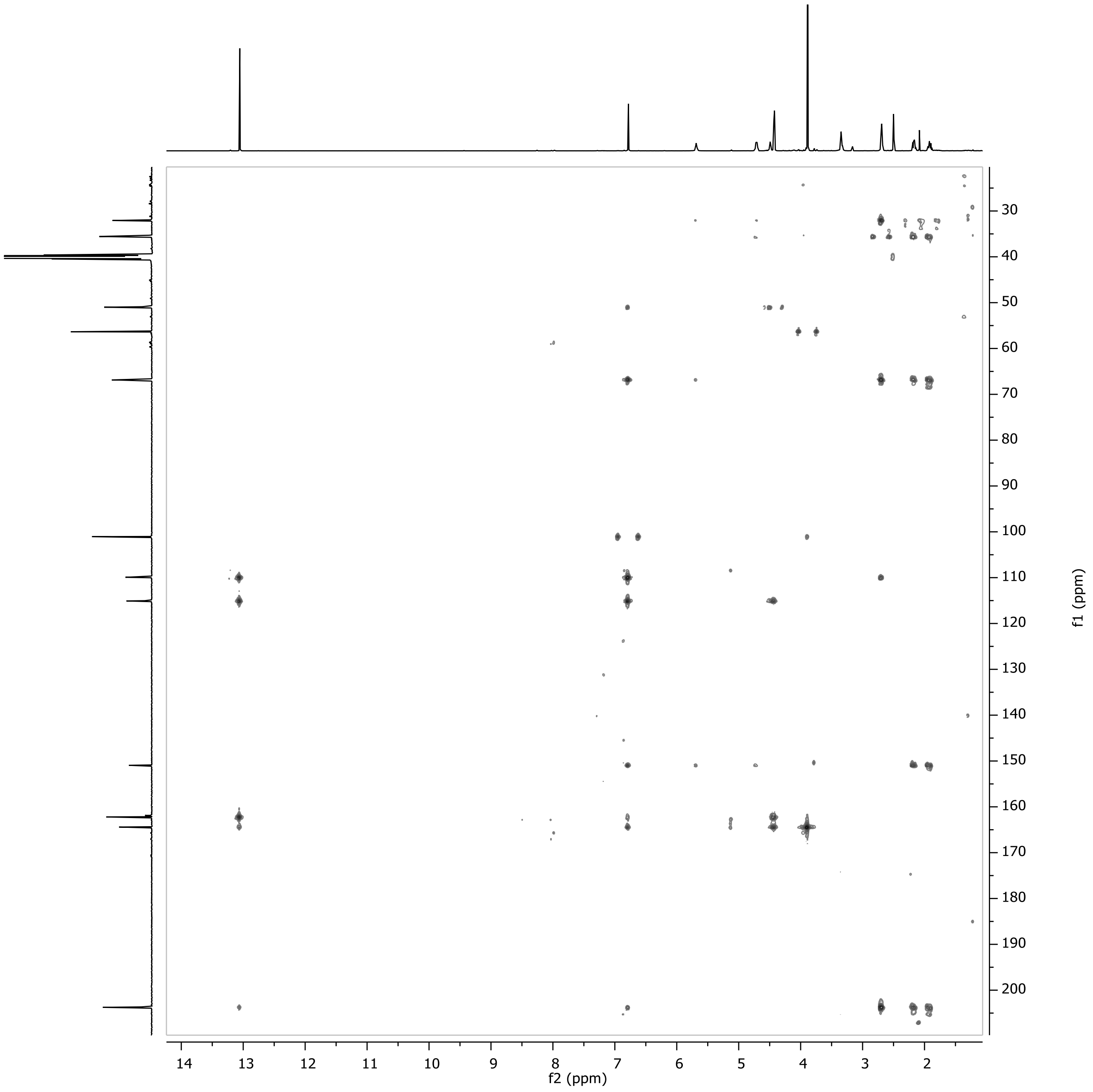

Figure S45. HMBC spectrum of **6** in DMSO-d_6 at 500 MHz.

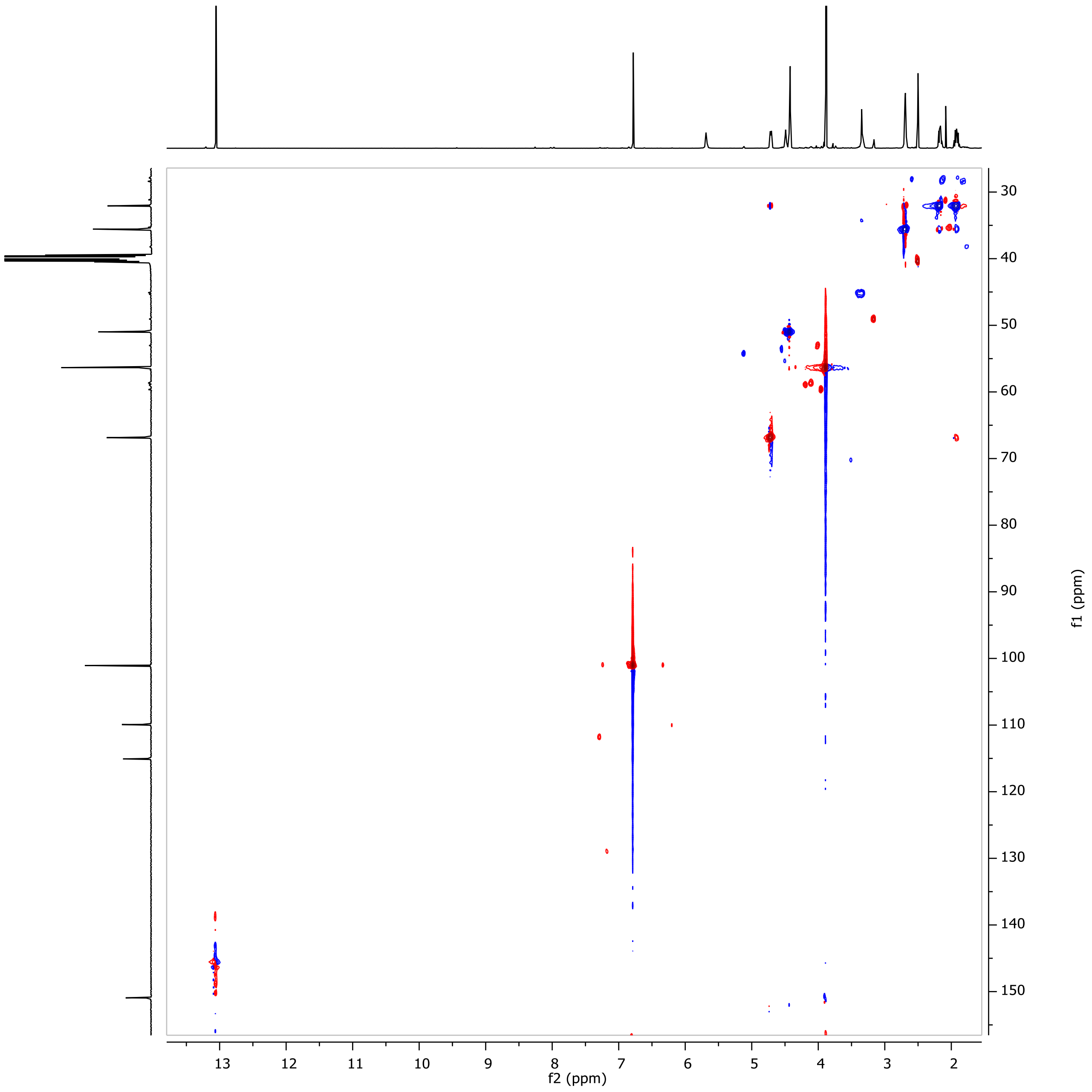

Figure S46. HSQC spectrum of **6** in DMSO-d_6 at 500 MHz.

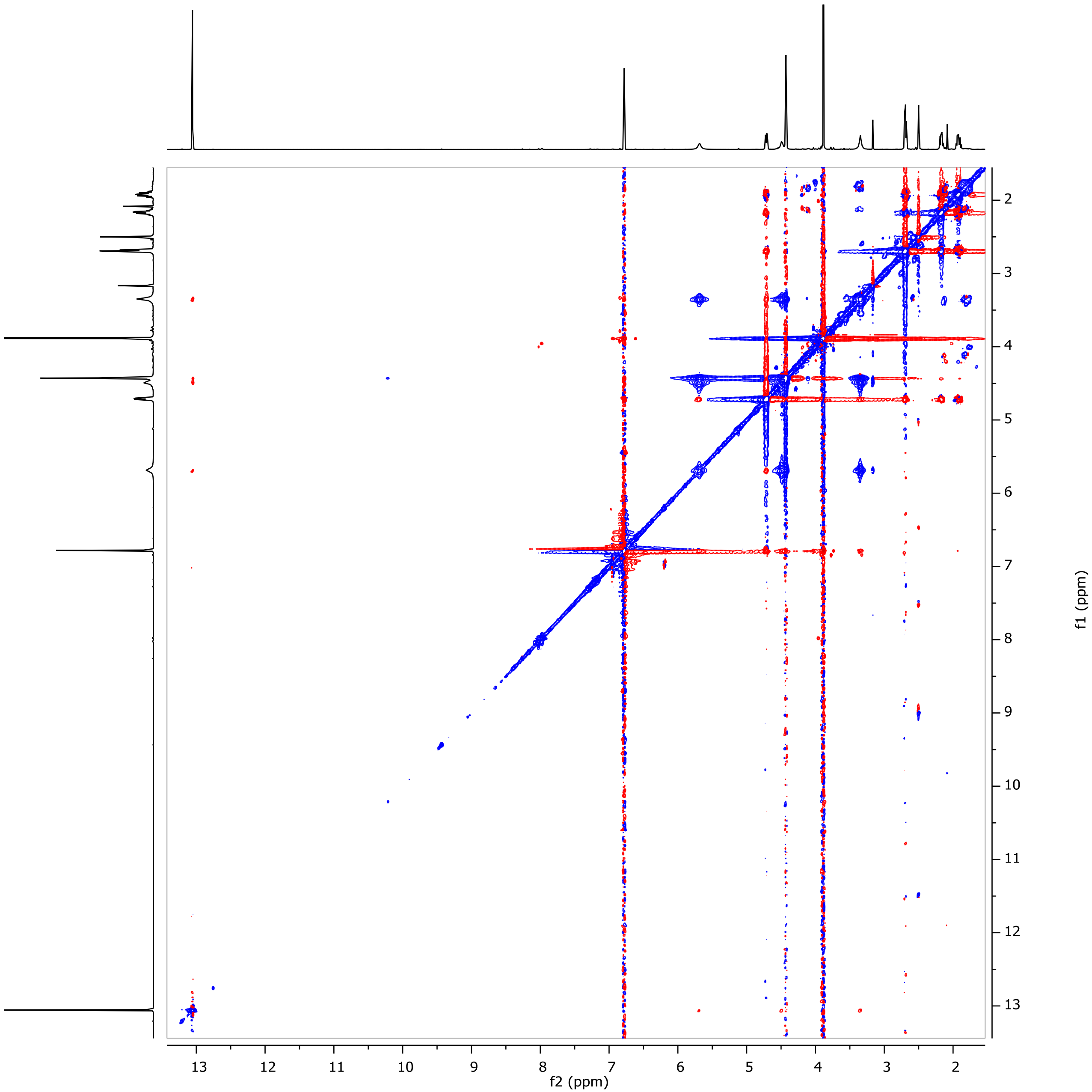

Figure S47. ROESY spectrum of **6** in DMSO-d_6 at 500 MHz.

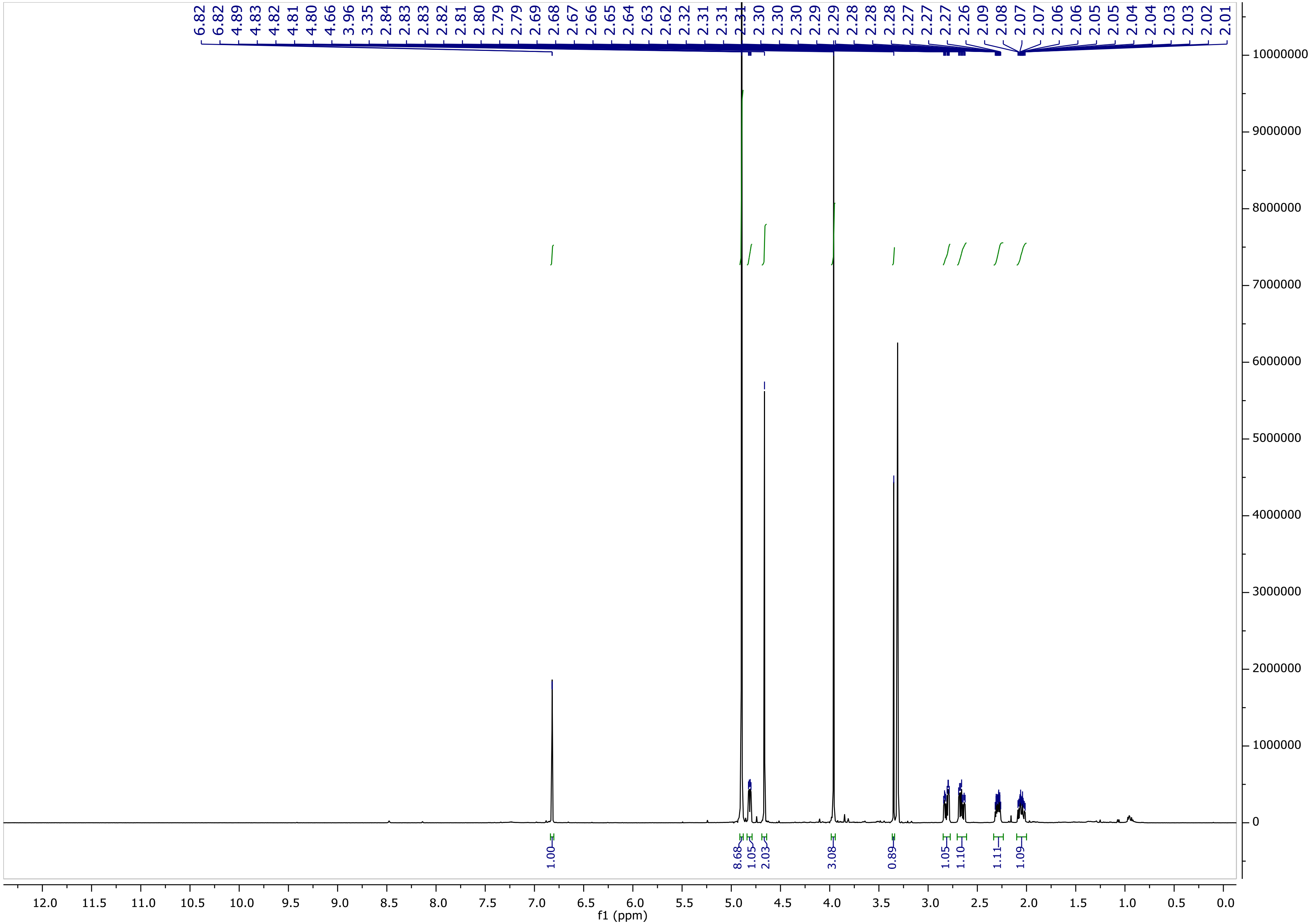

Figure S48. ^{1}H NMR spectrum of **6** in methanol-d_6 at 500 MHz.

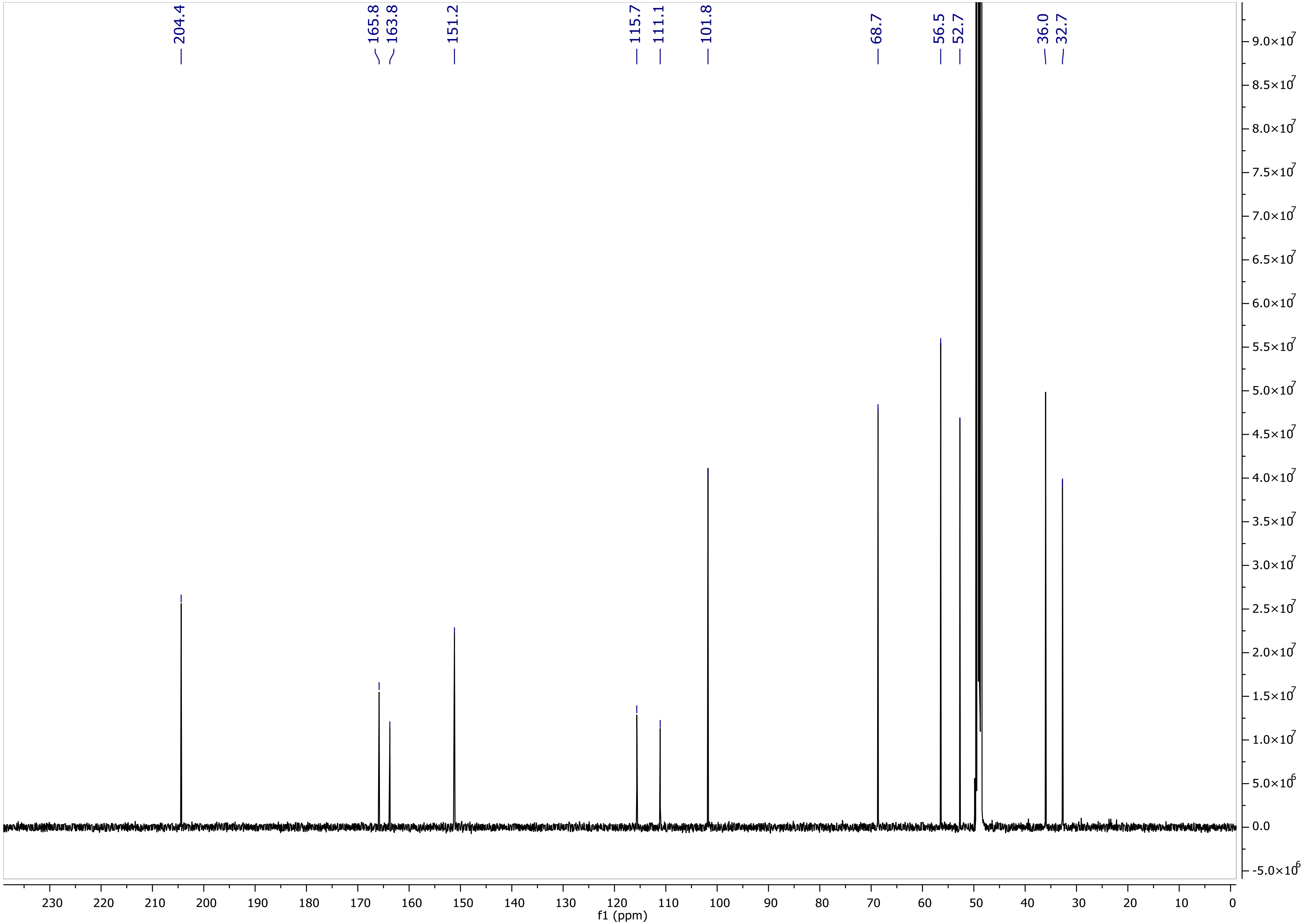

Figure S49. ^{13}C NMR spectrum of **6** in methanol-d_6 at 125 MHz.

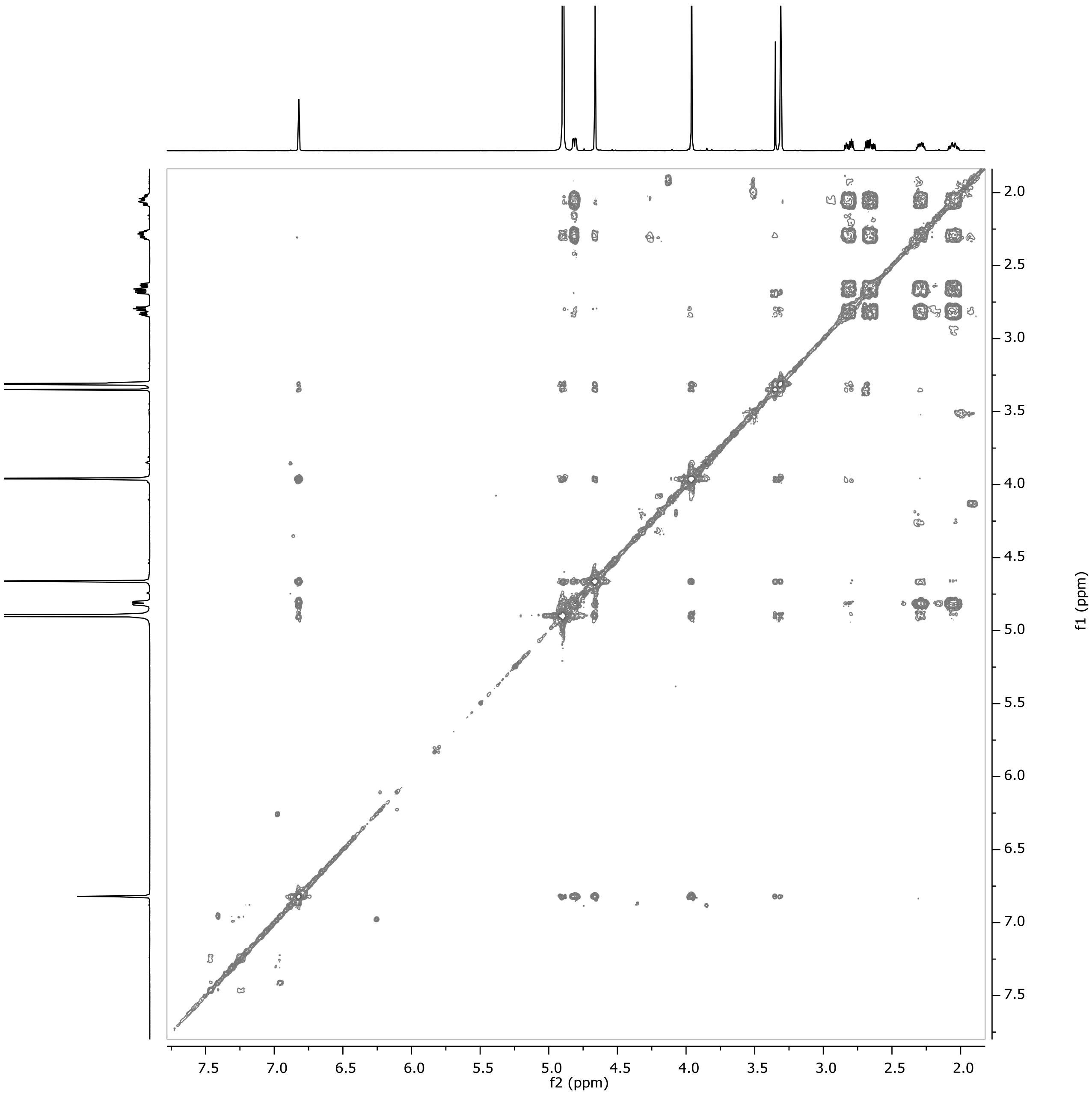

Figure S50. ^{1}H-^{1}H COSY spectrum of **6** in methanol-d_6 at 500 MHz.

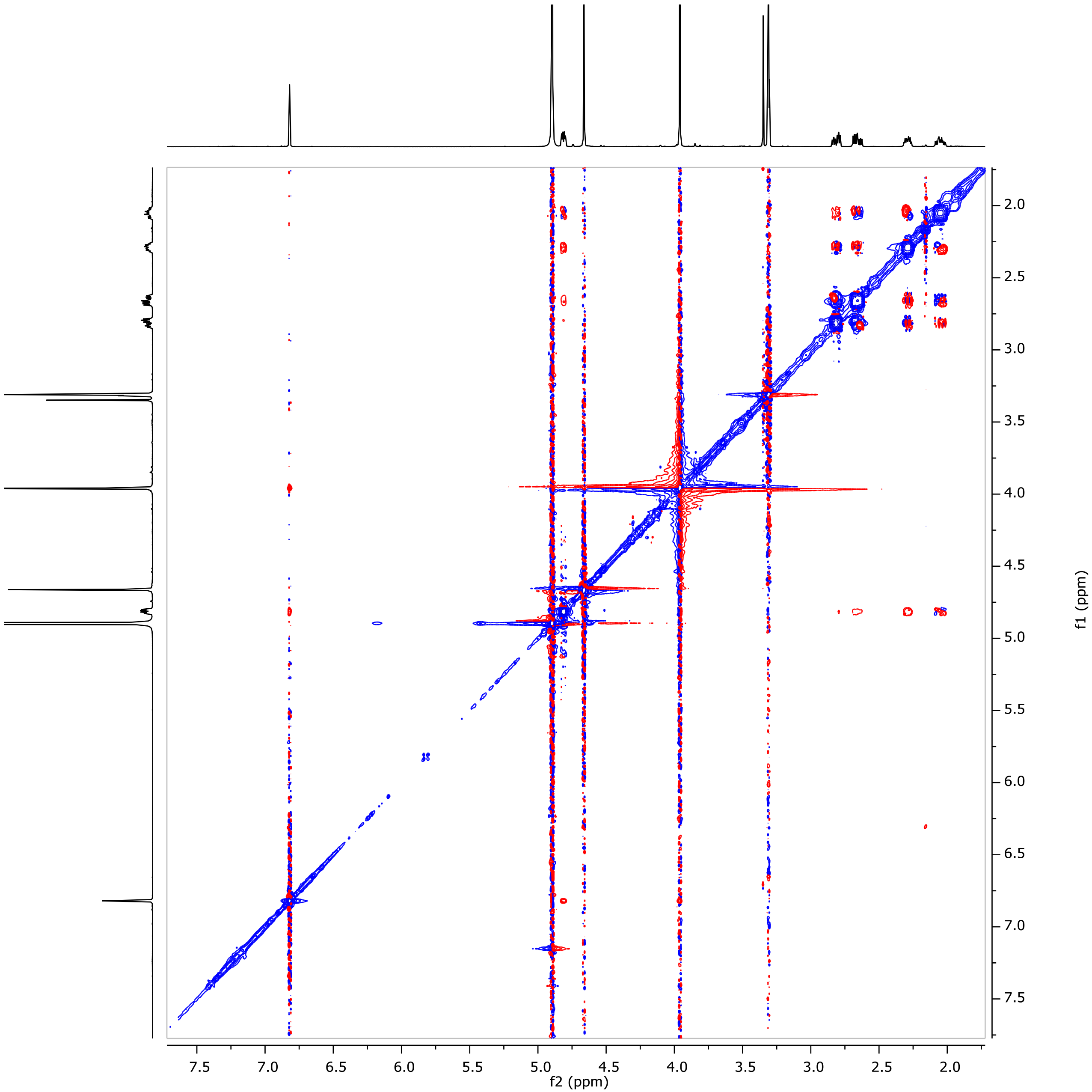

Figure S51. ROESY spectrum of **6** in methanol-d_6 at 500 MHz.

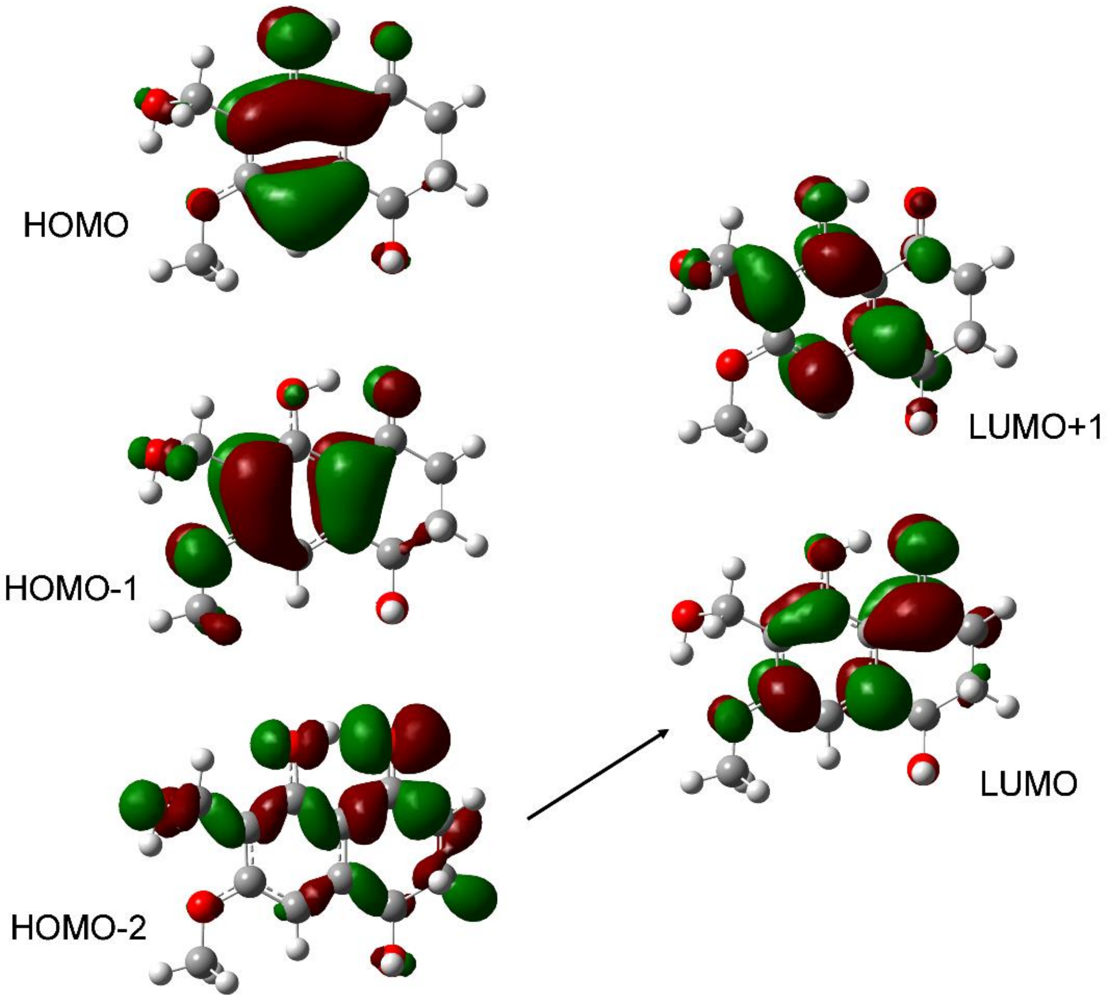

Figure S52. Kohn-Sham orbitals of (*R*)-**6** computed at the CAM-B3LYP/TZVP PCM/MeOH level for the lowest-energy ωB97X/TZVP PCM/MeOH conformer. Major contributors of the first four orbitals: 1. HOMO → LUMO, 2. HOMO-2 → LUMO, 3. HOMO-1 → LUMO, 4. HOMO-1 → LUMO+1. The arrow indicates the second transition corresponding to the n-π* CE of the tetralone chromophore.

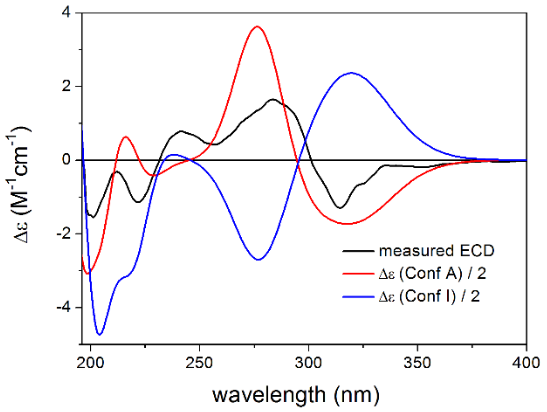

Figure S53. Experimental ECD spectrum of **5** in MeOH compared with the BH&HLYP/TZVP PCM/MeOH ECD spectra of the lowest-energy ωB97X/TZVP PCM/MeOH representatives of the two conformer groups of (*R*)-**5**; red: group A with a total population of 73.7% represented by conformer A (*M* helicity), blue: group B with a total population of 26.3% represented by conformer I (*P* helicity).

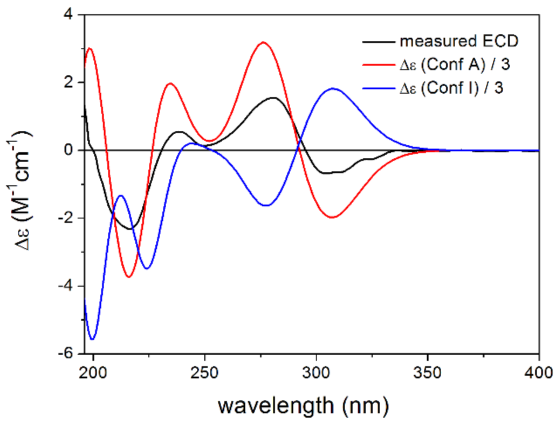

Figure S54. Experimental ECD spectrum of **6** in MeOH compared with the CAM-B3LYP/TZVP PCM/MeOH ECD spectra of the lowest-energy ωB97X/TZVP PCM/MeOH representatives of the two conformer groups of (*R*)-**6**; red: group A with a total population of 65.9% represented by conformer A (*M* helicity), blue: group B with a total population of 30.5% represented by conformer I (*P* helicity).

Table S1. Cytotoxicity (IC_{50}) and antimicrobial activity (MIC) of **1**–**6**.

	Strain number	IC50 (µM)						Positive Control
**Test Cell Line**		**1**	**2**	**3**	**4**	**5**	**6**	**Epothilone B (nM)**
Mouse fibroblast	L929	*	*	n.d.	49.02	**	*	0.65
Human endocervival adenocarcinoma	KB3.1	*	33.31	n.d.	63.02	76.01	*	0.17
**Test Microorganism**		**MIC (µg/mL)**						**Positive Control (µg/mL)**
Bacillus subtilis	DSM 10	-	-	n.d.	-	-	-	16.6^{O}
Mycobacterium smegmatis	ATCC 700084	n.d	-	n.d.	-	-	-	1.70^{K}
Staphylococcus aureus	DSM 346	-	66.6	n.d.	-	-	-	0.21^{G}
Acinetobacter baumanii	DSM 30008	n.d.	-	n.d.	-	-	-	0.52^{C}
Chromobacterium violaceum	DSM 30191	n.d	-	n.d.	-	-	-	1.70^{G}
Escherichia coli	DSM 1116	n.d.	-	n.d.	-	-	-	0.42^{G}
Pseudomonas aeruginosa	DSM 19882	n.d	n.i	n.d.	-	-	-	0.21^{G}
Aspergillus fumigatus	ATCC 204305	0.26	0.52	8.325	n.d.	n.d.	n.d.	0.312^{A}
A. fumigatus	CCF3522	n.d	0.52	n.d	n.d	n.d	n.d	0.312^{A}
A. fumigatus	CCUG75301	n.d	n.d	-	n.d	n.d	n.d	1.25^{A}
A. fumigatus (azole-resistant)	CCF 6651	-	2.08	0.52	n.d	n.d	n.d	0.156^{A}
A. fumigatus (azole-resistant)	CCF 6674	16.6	0.52	0.52	n.d	n.d	n.d	2.5^{A}
Candida albicans	DSM 1665	-	-	n.d.	-	-	-	8.3^{N}
C. albicans	CCM8215	-	-	8.3	n.d.	n.d.	n.d.	1.25^{A}
Cryptococcus neoformans	CCF1081	-	4.15	4.15	n.d.	n.d.	n.d.	2.5^{A}
Mucor hiemails	DSM 2656	-	-	n.d.	-	-	-	8.30^{N}
Mucor plumbeus	CCF2612	-	-	1.04	n.d.	n.d.	n.d.	0.312^{A}
Rhodotorula glutinis	DSM 10134	-	8.3	n.d.	-	-	-	4.20^{N}
Schizosaccharomyces pombe	DSM 70572	-	-	n.d.	-	-	-	8.30^{N}
Wickerhamomyces anomalus	DSM 6766	-	-	n.d.	-	-	-	16.6^{N}

(*): Slight inhibition of cell proliferation, (**): no cytotoxic activity observed. (-): no inhibition up to 67 µg/mL. n.d.: not determined. G: gentamycin; O: oxytetracycline; N: nystatin; C: ciprofloxacin; K: kanymycin; A: amphotericin

Table S2. Nematicidal activity of **1**, **2**, **4**–**6**.

Test Organism	Strain number	Conc. (µg mL^{-1})	Corrected Mortality (%)					Positive Control
			**1**	**2**	**4**	**5**	**6**	**Ivermectin**
Caenorhabtidis elegans	N2	100	7.1 ± 3.3	< 0	0.8 ± 2.1	< 0	2.0 ± 2.1	n.d.
		50	2.1 ± 2.4	< 0	1.1 ± 2.6	< 0	5.5 ± 3.7	
		10	4.5 ± 1.7	< 0	1.6 ± 0.6	< 0	3.5 ± 1.9	
		1	n.d.	n.d.	n.d.	n.d.	n.d.	97.0 ± 2.0

n.d.: not determined. < 0: less active then the corresponding negative control.
